# Mice depleted for Exchange Proteins Directly Activated by cAMP (Epac) exhibit irregular liver regeneration in response to partial hepatectomy

**DOI:** 10.1038/s41598-019-50219-8

**Published:** 2019-09-24

**Authors:** Kathrine Sivertsen Åsrud, Line Pedersen, Reidun Aesoy, Haruna Muwonge, Elise Aasebø, Ina Katrine Nitschke Pettersen, Lars Herfindal, Ross Dobie, Stephen Jenkins, Rolf Kristian Berge, Neil Cowan Henderson, Frode Selheim, Stein Ove Døskeland, Marit Bakke

**Affiliations:** 1 Department of Biomedicine, The University of Bergen, Bergen, Norway; 2 Department of Clinical Science, The University of Bergen, Bergen, Norway; 3 Department of Biomedicine, The Proteomic Unit at The University of Bergen (PROBE), University of Bergen, 5009 Bergen, Norway; 4 Centre for Inflammation Research, The Queen’s Medical Research Institute, The University of Edinburgh, Edinburgh, UK; 5 Department of Heart Disease, Haukeland University Hospital, Bergen, Norway

**Keywords:** Proteomics, Proteomics, Non-alcoholic fatty liver disease, Molecular medicine, Molecular medicine

## Abstract

The exchange proteins directly activated by cAMP 1 and 2 (Epac1 and Epac2) are expressed in a cell specific manner in the liver, but their biological functions in this tissue are poorly understood. The current study was undertaken to begin to determine the potential roles of Epac1 and Epac2 in liver physiology and disease. Male C57BL/6J mice in which expression of Epac1 and/or Epac2 are deleted, were subjected to partial hepatectomy and the regenerating liver was analyzed with regard to lipid accumulation, cell replication and protein expression. In response to partial hepatectomy, deletion of Epac1 and/or Epac2 led to increased hepatocyte proliferation 36 h post surgery, and the transient steatosis observed in wild type mice was virtually absent in mice lacking both Epac1 and Epac2. The expression of the protein cytochrome P4504a14, which is implicated in hepatic steatosis and fibrosis, was substantially reduced upon deletion of Epac1/2, while a number of factors involved in lipid metabolism were significantly decreased. Moreover, the number of Küpffer cells was affected, and Epac2 expression was increased in the liver of wild type mice in response to partial hepatectomy, further supporting a role for these proteins in liver function. This study establishes hepatic phenotypic abnormalities in mice deleted for Epac1/2 for the first time, and introduces Epac1/2 as regulators of hepatocyte proliferation and lipid accumulation in the regenerative process.

## Introduction

Diseases related to hepatic steatosis constitute an increasing health problem worldwide. A better understanding of the underlying molecular mechanisms in healthy and diseased liver tissue is required for understanding of etiology, and for the development of efficient treatment strategies. The second-messenger cAMP is a critical regulator of hepatic metabolic activity, and was in fact first described due to its effects on glycogenolysis in response to glucagon and epinephrine^1^. cAMP is also a central player in the control of liver lipogenesis^2^, and has been implicated in the transcriptional signaling responses that orchestrate liver regeneration in mammals in response to partial hepatectomy (PH)^3^. In general, the effects of cAMP in the liver have been ascribed to activation of the cAMP dependent protein kinase A (PKA), but numerous observations also link the exchange proteins directly activated by cAMP (Epac; also known as cAMP-guanine nucleotide exchange factor, cAMP-GEF) to hepatic cellular processes^4^.

The Epac proteins, Epac1 and Epac2 (hereafter collectively referred to as Epac1/2) are multi-domain proteins that function as cAMP-activated exchange factors for the small G-proteins Rap1 and Rap2^5,6^. Epac1/2 are implicated in a wide range of biological processes including glucose stimulated insulin secretion, leptin signaling, neuronal growth and differentiation, and memory and learning (reviewed in^7^). Epac1 and Epac2 are encoded by *Rapgef3* and *Rapgef4*, respectively, and differential splicing and alternative promoter usage in combination with epigenetic regulation of *Rapgef4*, give rise to three different Epac2 isoforms, Epac2A, Epac2B and Epac2C^8–10^. Whereas Epac1 is nearly ubiquitously expressed, Epac2 isoforms exhibit a strict tissue specific expression pattern. Of particular interest for the current study is that expression of the shortest isoform, Epac2C, is confined to hepatocytes^10^. Epac2C lacks the N-terminal cyclic nucleotide-binding domain (cNBD-A) found in Epac2A, which is required for insulin secretion^11^, and the Dishwelled-Egl-10-pleckstrin (DEP) domain found in Epac2A, Epac2B and in Epac1^12^, that is essential for membrane association^11^. Overall, the Epac2C isoform has been sparsely studied. It is known that hepatic stellate cells (HSC) express Epac1^13^, and cholangiocytes, both Epac1 and Epac2A^14^, and *in vitro* studies link Epac1/2 to hepatic cellular processes, such as formation of canalicular networks^15^, proliferation of cholangiocytes^14^ and glucagon-dependent secretion of the fibroblast growth factor 21 (Fgf21) from hepatocytes^16^. In HSC, Epac1 has been implicated in transforming growth factor β1 (TGFβ1) induced signaling^13^, which is believed to integrate pro- and anti-fibrotic signals^13,17,18^.

However, the functional roles of Epc1/2 in liver have not yet been studied in intact animals, and moreover, many of the *in vitro* studies undertaken to investigate the roles of Epac1/2 in hepatic cells have monitored the effects of the cAMP analogue 8-pCPT-2‘-O-Me-cAMP (8-pCPT). Although first described as a specific Epac1/2 agonist^19^, it is now clear that 8-pCPT and its derivatives have off target effects^20–25^, and that it is a rather poor activator of Epac2^26^. Therefore, to delineate the roles of Epac1/2 in liver physiology we employed mouse models that are deleted for Epac1 (Epac1^−/−^), Epac2 (Epac2^−/−^; all isoforms), or a combined knockout model of both factors (Epac1/2^−/−^). The mice were subjected to partial hepatectomy (PH), which is widely used to gain insights into the mechanisms that control signaling, proliferation and growth in the liver^27^. We report here that PH provokes phenotypes in Epac1/2^−/−^ mice that are related to cell proliferation and lipid turnover in the regenerating liver. Moreover, we found that Epac2C expression (mRNA and protein) were increased in response to PH, and comparison of the proteome of wild type (wt) and Epac1/2^−/−^ mice demonstrated altered expression profiles of proteins related to hepatic lipid metabolism and disease development.

## Results

### Liver histology is normal in Epac1^−/−^, Epac2^−/−^ and Epac1/2^−/−^ mice

**Figure 1:**
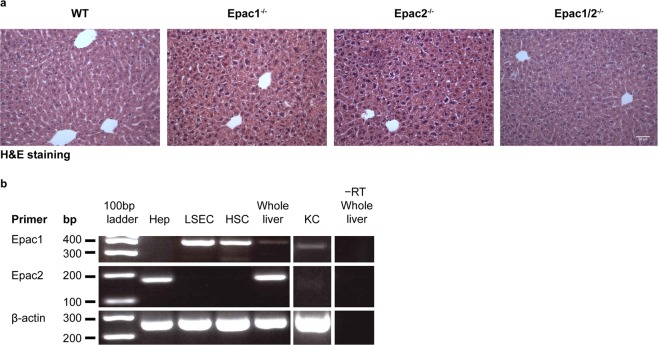
Expression of Epac1/2 in liver cells. (**a)** H&E (20x) staining of liver sections from wt, Epac1^−/−^, Epac2^−/−^ and Epac1/2^−/−^ mice. Bar: 100 μm. (**b**) RT-PCR of Epac1 and Epac2 mRNA in hepatocytes (Hep), liver sinusoidal endothelial cells (LSEC), hepatic stellate cells (HSC), Küpffer cells (KC) and whole liver. −RT: without reverse transcriptase.

Histological analysis revealed no apparent abnormalities in liver morphology in Epac1^−/−^, Epac2^−/−^ or Epac1/2^−/−^ mice (Fig. 1a). Moreover, no differences in liver mass were observed in adult mice of the different genotypes, indicating that deletion of Epac1/2 does not affect post-natal liver growth (Supplementary Fig. S1). Since Epac1/2 signaling has been implicated in bile acid stimulated hepatocyte polarization^15^, we performed immunohistochemistry (IHC) analyses against multidrug resistance protein 1B (ABCB1) to label the apical hepatocyte membrane. The distribution of ABCB1 in wt and Epac1/2^−/−^ mice was indistinguishable (Supplementary Fig. S2a), and canalicular length per cell was unaffected by Epac1/2 deletion both pre- and post-PH (Supplementary Fig. S2b). RT-PCR was performed to determine the expression of Epac1/2 in different hepatic cells (Fig. 1b). The primer pair used in the present study to amplify Epac2 transcripts recognizes all Epac2 isoforms, but we have demonstrated previously, both at mRNA and protein level, that the mouse liver only expresses the Epac2C isoform^8^. Epac1 transcripts were identified in sinusoidal endothelial cells, Küpffer (KC) cells and HSC, whereas Epac2C was found solely in hepatocytes (Fig. 1b). Thus, none of the cell types analyzed were found to co-express Epac1 and Epac2.

### Deletion of Epac1/2 accelerates DNA replication after PH

**Figure 2:**
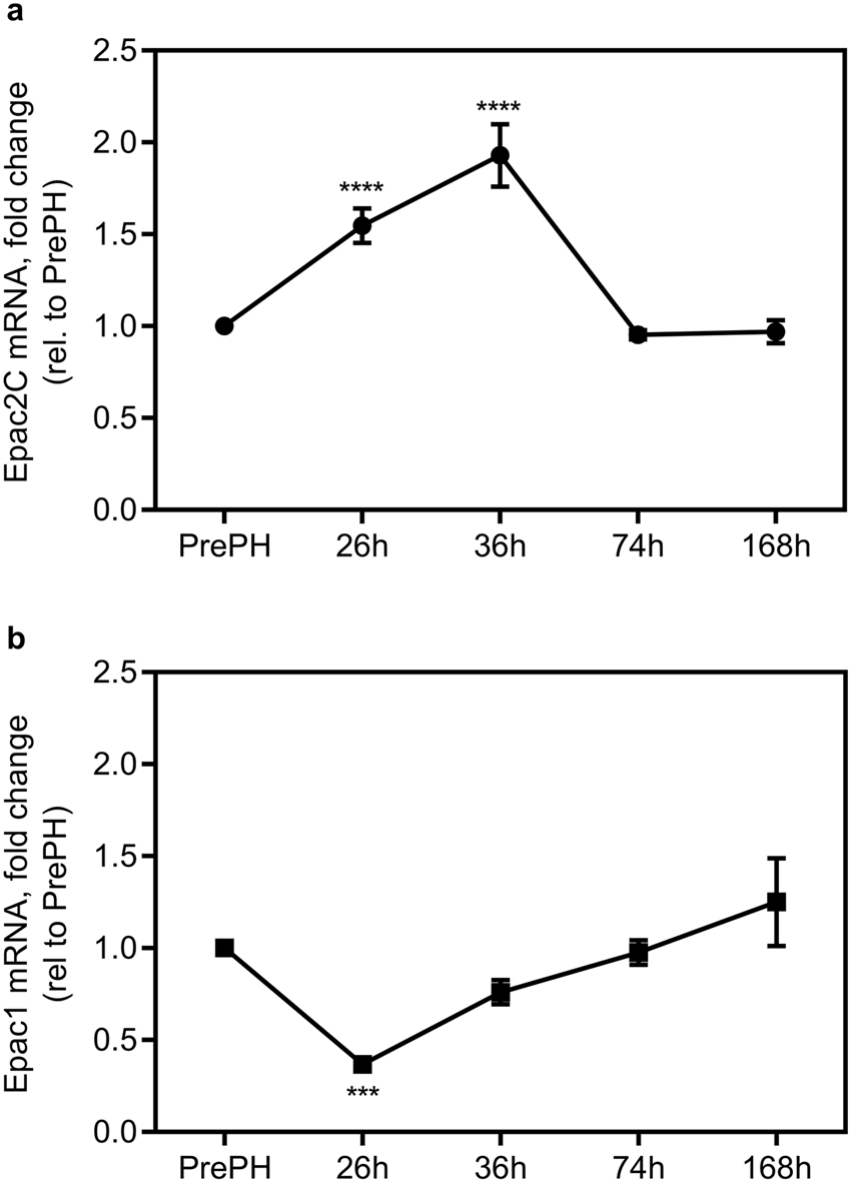
The expression of Epac2C mRNA is increased in liver after PH. qPCR analysis of (**a**) Epac2C and (**b**) Epac1 mRNA in liver pre-PH or 26 h, 36 h, 74 h and 168 h post-PH in wt mice. The CT values for resected tissue (4.39 ± 0.12 for Epac2C and 12.29 ± 0.38 for Epac1) were set to 1. Data are expressed as mean ± SD of three separate experiments performed in triplicates. One-way ANOVA with Dunnett’s adjustment for multiple comparisons was used to determine statistical differences. ***p < 0.001 and ****p < 0.0001: mRNA levels in wt livers pre-PH compared to mRNA levels in wt livers at the different postoperative time points (26 h, 36 h, 74 h or 168 h post-PH). F-statistics (**a**): F(4,9) = 75.57, p < 0.0001 and (**b**): F(4,10) = 24.50, p < 0.0001.

To gain insight into the functional roles of Epac1/2 in the liver, wt, Epac1^−/−^, Epac2^−/−^ and Epac1/2^−/−^ mice were subjected to PH and euthanized at four post-operative time points (26 h, 36 h, 74 h and 168 h). Mice depleted for Epac1/2 displayed no adverse phenotypes in response to PH compared to wt mice (*i.e*. no change in post-operative hepatocellular necrosis or mortality), and the resected liver- to- body weight ratio was similar in all genotypes (Supplementary Fig. S3a), as was the restitution of hepatic index (Supplementary Fig. S3b). qPCR was performed to determine whether PH affects the expression of Epac1/2 in wt mice. As shown in Fig. 2a, Epac2c mRNA expression in total liver was increased at 26 h and 36 h post-PH, but then returned to basal levels. This finding correlates with increased Epac2C protein expression as determined by proteomic analyses (Supplementary file 2). In contrast, Epac1 mRNA levels decreased at 26 h post-PH (Fig. 2b). However, confounding the relevance of this finding is the very low level of Epac1 mRNA in total liver (see legend to Fig. 2 for ct-values), as also evident from undetectable levels of Epac1 in proteomic analyses of total liver (Supplementary file 2).

**Figure 3:**
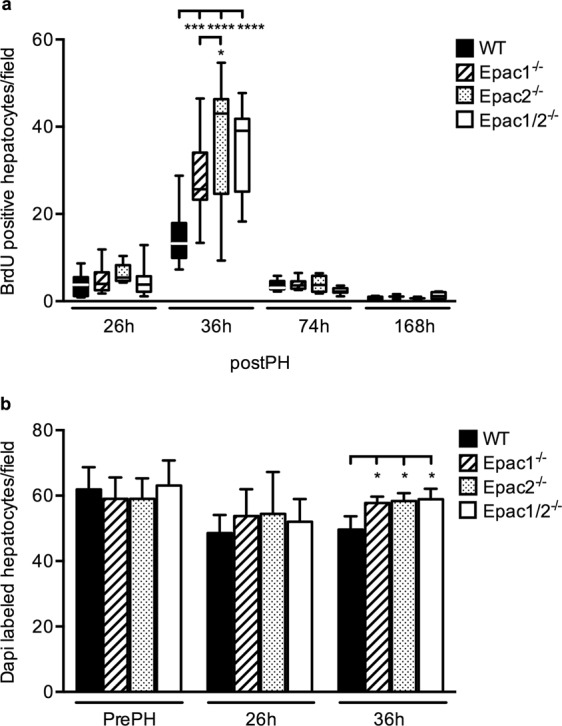
Increased BrdU-incorporation 36 h post-PH in Epac1^*−/−*^, Epac2^*−/−*^ and Epac1/2^*−/−*^ mice. **(a**) BrdU incorporation was analyzed in wt, Epac1^−/−^, Epac2^−/−^ and Epac1/2^−/−^  mice post-PH for the indicated time points. Data is shown as box and whiskers with median and min to max whiskers, n = 2–13 mice/group (6–13 mice/group at 26 h and 36 h). (**b**) Quantification of DAPI staining pre-PH, 26 h and 36 h post-PH. Data is shown as mean ± SD, n = 3–9 mice/group. Two-way ANOVA with Bonferroni’s adjustment for multiple comparisons was used to determine statistical differences between genotypes within each time point. *p < 0.05, ***p < 0.001 and ****p < 0.0001. F-statistics; (**a**) F(9,104) = 5.331, p < 0.0001 and (**b**) F(6,55) = 1.436, p = 0.2176. (**a**) 36 h post-PH (mean ± SD): wt: 15.04 ± 6.61; Epac1^−/−^: 27.97 ± 10.22; Epac2^−/−^: 36.57± 15.32; Epac1/2^−/−^: 34.86 ± 9.37. (**b**): 36 h post-PH (mean ± SD): wt: 49.6 ± 4.1; Epac1^−/−^: 57.8 ± 1.9; Epac2^−/−^: 58.4 ± 2.4; Epac1/2^−/−^: 58.9 ± 3.2.

To determine whether the proliferative capacity after PH was affected as a consequence of deleting Epac1/2, BrdU incorporation in hepatocytes was analyzed to quantify DNA synthesis. In wt mice, BrdU-positive hepatocytes were detectable at 26 h, peaked at 36 h and returned to basal levels 74 h after PH, similar to previously reported^28^. Interestingly, the number of BrdU-positive hepatocytes was substantially increased in Epac1^−/−^, Epac2^−/−^ and Epac1/2^−/−^ mice compared to wt mice 36 h post-PH (Fig. 3a), demonstrating that more hepatocytes had entered the S-phase. The increased BrdU incorporation was accompanied by an increased number of DAPI stained nuclei per area 36 h post-PH (Fig. 3b). Since the hepatocyte size was similar in wt and Epac1/2^−/−^ mice (Supplementary Fig. S3c), these results indicate increased hepatocyte proliferation in Epac1^−/−^, Epac2^−/−^ and Epac1/2^−/−^ mice, and not increased hepatocyte hypertrophy, which is often observed after PH^29^. The increased proliferation of hepatocytes in Epac1/2^−/−^ mice was a result of PH, as DAPI staining revealed similar numbers of hepatocytes in resected tissue sections of all genotypes before PH, and because BrdU incorporation was equal in response to sham operation in all genotypes (Supplementary Fig. S3d).

Proteomic analyses indicated no changes in the expression of cyclin dependent kinases (Cdks) in Epac1/2^−/−^ mice compared to wt mice (Supplementary dataset file 2). We were unable to detect other major cell cycle factors by this method, and therefore performed immunoblotting experiments. The results demonstrated similar levels of expression of Cyclin D1 and Cyclin E1 in the different genotypes (Supplementary Fig. S4a–l). In contrast, the expression of Interleukin 1 (IL-1), a suppressor of hepatocyte proliferation^30^, was moderately decreased in Epac1^−/−^ mice 26 h post-PH (Supplementary Fig. S4a–d,m–p). Furthermore, the levels of Tumor Necrosis Factor alpha (TNFα), a potent pro inflammatory cytokine^31^, was increased in both Epac1^−/−^ and Epac2^−/−^ mice compared to wt mice pre-PH, with a more pronounced increase in Epac1^−/−^ mice (Fig. S4a–d,q–p), and down-regulated in Epac1/2^−/−^ mice 36 h post-PH (Fig. S4a–d,q–p) (see Supplementary Fig. S5 for the full- length blots).

### The regenerating liver of Epac1/2^−/−^ mice display early loss of fat vacuoles

**Figure 4:**
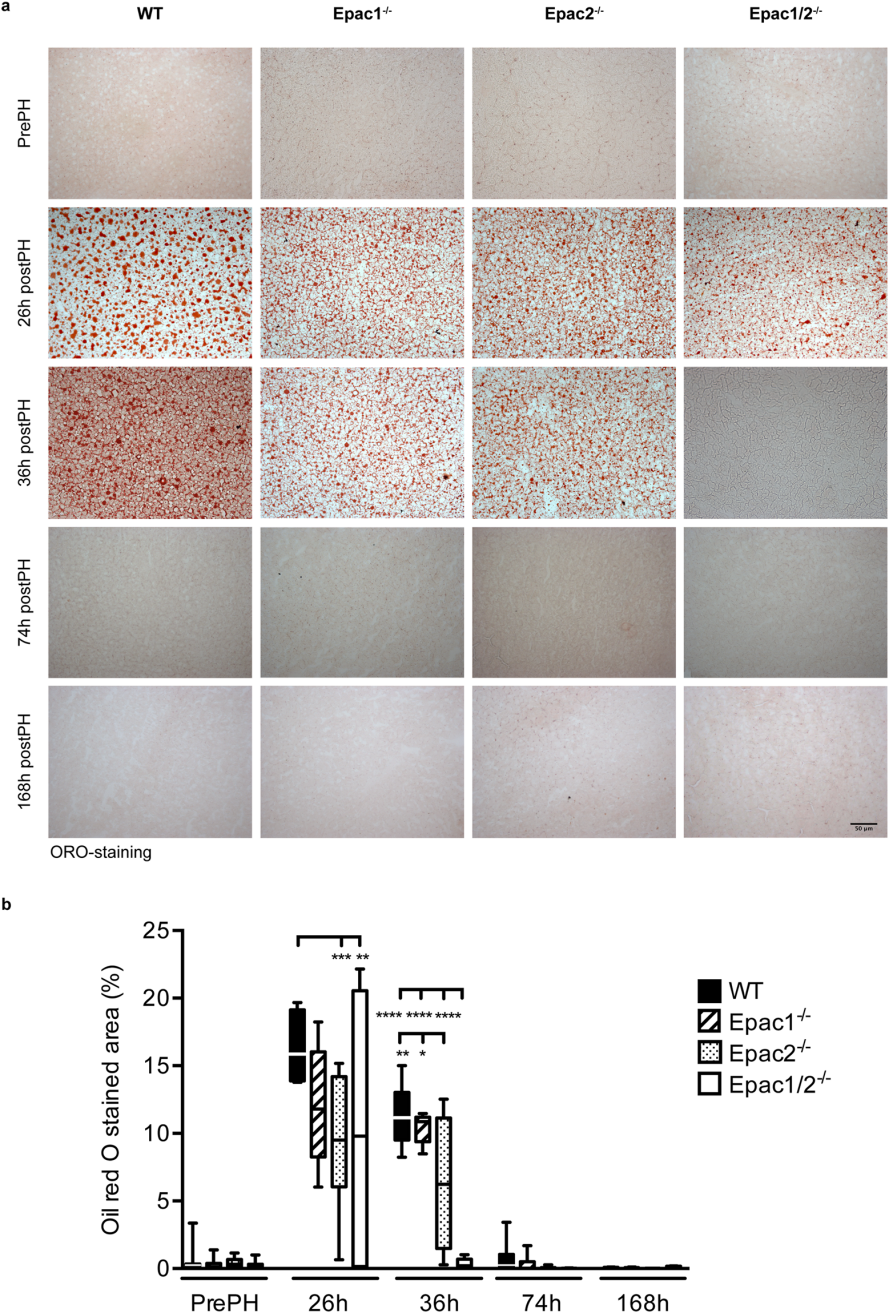
Epac1/2^*−/−*^ mice are devoid of  fat vacuoles 36 h post-PH. ORO staining of sections from livers from wt, Epac1^−/−^, Epac2^−/−^ and Epac1/2^−/−^ mice pre- and post-PH as indicated (20x). (**a**) Representative images of ORO staining. (**b**) Quantification of ORO-staining. The median and left lateral lobes resected during surgery were used as control tissue in these experiments. Data are shown as box and whiskers with median and min to max whiskers, n = 4–8 mice/group post-PH and n = 17–23 mice/group pre-PH. Two-way ANOVA with Bonferroni’s adjustment for multiple comparisons was used to determine statistical differences between genotypes within each time point. *p < 0.05, **p < 0.01, ***p < 0.001 and ****p < 0.0001. F-statistics: F(12,149) = 5.152, p < 0.0001.

Mice accumulate hepatic lipid droplets in response to PH, and this condition of steatosis is particularly evident 20–40 h post-PH^32^. Oil Red O (ORO) staining of the right and caudate lobes that remained after PH revealed extensive lipid accumulation in wt mice at 26 h and 36 h post-PH, followed by a return to basal levels at later time points (Fig. 4). Lipid droplets were also observed in Epac1^−/−^, Epac2^−/−^ and Epac1/2^−/−^ mice 26 h post-PH. The size, shape and distribution of the microvesicular fat vacuoles were comparable in all genotypes at this time point, although quantitative determination of the ORO staining revealed notable individual variations between animals within the same experimental group (Fig. 4b). This was especially evident in Epac1/2^−/−^ mice where some individuals exhibited moderate steatosis 26 h post-PH, while others presented with a phenotype completely devoid of fat vacuoles (Fig. 4b). Of note, all Epac1/2^−/−^ mice presented with a striking absence of fat vacuoles 36 h post-PH (Fig. 4a). Reduced fat accumulation was also evident in Epac2^−/−^ mice at both time points (26 h and 36 h post-PH) compared to wt mice (Fig. 4).

### Altered lipid profiles and protein expression in the absence of Epac1/2

Plasma lipid profiling revealed decreased basal (pre-PH) concentrations of circulating total cholesterol in Epac1^−/−^, Epac2^−/−^ and Epac1/2^−/−^ mice compared to wt mice (Table 1), as previously described in Epac1^−/−^ mice^33^. The reduction in total cholesterol correlated with reduced levels of high-density lipoprotein (HDL) cholesterol, as neither low-density lipoprotein (LDL) cholesterol nor unesterified cholesterol levels were changed (Table 1). In accordance with the reduced level of HDL cholesterol, circulating phospholipid was also reduced in all Epac1/2 knockout models prior to PH. Moreover, the basal levels of triglycerides (TG) was elevated in plasma of Epac1/2^−/−^ mice (Table 1), but the potential significance of this increase is currently unclear. The Epac1/2 knockout models exhibited reduced plasma levels of total cholesterol, HDL cholesterol, phospholipids (PL) and TGs, and elevated LDL cholesterol levels 36 h post-PH, compared to pre-PH conditions. The wt mice showed a similar profile, although the increase in LDL cholesterol was not significant (Table 1). Of note, Epac1/2^−/−^ mice had a significantly higher level of plasma LDL cholesterol than wt and Epac2^−/−^ mice post-PH, indicating increased LDL cholesterol secretion or decreased LDL cholesterol uptake in peripheral tissues. The concentration of circulating free fatty acids (FFA) was similar before and 36 h after PH in all genotypes (Table 1), demonstrating that the concentration of circulating FFA that peaks around 20 h post-PH in mice^34^, returns to normal also in the absence of Epac1/2.

**Table 1:** Altered lipid profile in Epac^−/−^ mice.

		WT	Epac1^−/−^	Epac2^−/−^	Epac1/2^−/−^
Control					
Plasma, mmol/l	C	2.75 ± 0.09	2.26 ± 0.21^aa^	2.25 ± 0.13^aa^	2.37 ± 0.21^aa^
	HDL-C	2.38 ± 0.03	1.91 ± 0.37^aaa^	1.99 ± 0.18^aa^	1.90 ± 0.16^aaaa^
	LDL-C	0.20 ± 0.05	0.21 ± 0.03	0.12 ± 0.04	0.23 ± 0.10
	UC	0.80 ± 0.08	0.75 ± 0.06	0.74 ± 0.04	0.78 ± 0.05
	TG	1.19 ± 0.33	1.34 ± 0.14	1.44 ± 0.20	1.54 ± 0.31^a^
	NEFA	0.23 ± 0.14	0.017 ± 0.04	0.10 ± 0.13	0.11 ± 0.01
	PL	2.99 ± 0.12	2.60 ± 0.03^a^	2.56 ± 0.16^aa^	2.69 ± 0.16^a^
Liver, μmol/g	C	6.35 ± 0.43	7.77 ± 2.09	6.35 ± 0.51	6.69 ± 0.34
	TG	5.25 ± 0.91	5.73 ± 0.46	8.86 ± 3.07	5.01 ± 0.50
	NEFA	0.61 ± 0.10	0.55 ± 0.08	0.55 ± 0.05	0.55 ± 0.06
	PL	21.36 ± 0.76	20.58 ± 0.86^dd^	21.47 ± 0.69	22.54 ± 0.54
36 h post PH					
Plasma, mmol/l	C	1.62 ± 0.17^bbbb^	1.68 ± 0.17^bbb^	1.58 ± 0.23^bbbb^	1.76 ± 0.22^bbbb^
	HDL-C	1.21 ± 0.19^bbbb^	1.25 ± 0.13^bbbb^	1.17 ± 0.16^bbbb^	1.22 ± 0.18^bbbb^
	LDL-C	0.29 ± 0.04^ccc^	0.41 ± 0.09^bb^	0.32 ± 0.13^bbb, cccc^	0.54 ± 0.02^bbbb^
	UC	0.75 ± 0.08	0.70 ± 0.05	0.73 ± 0.08	0.75 ± 0.09
	TG	0.68 ± 0.08^bbbb^	0.65 ± 0.05^bbbb^	0.57 ± 0.14^bbbb^	0.54 ± 0.16^bbbb^
	NEFA	0.16 ± 0.11	0.27 ± 0.15	0.23 ± 0.18	0.14 ± 0.07
	PL	1.77 ± 0.19^bbbb^	1.74 ± 0.16^bbbb^	1.68 ± 0.23^bbbb^	1.74 ± 0.24^bbbb^
Liver, μmol/g	C	9.22 ± 0.82^bbbb^	9.95 ± 0.90^bb, c^	8.91 ± 1.01^bbb^	7.81 ± 1.49
	TG	78.78 ± 45.62^bb^	39.47 ± 20.90	56.04 ± 46.97	7.68 ± 1.65
	NEFA	1.22 ± 0.41	0.94 ± 0.22	0.98 ± 0.41	0.66 ± 0.10
	PL	19.80 ± 1.10^bb^	19.47 ± 0.91	20.38 ± 0.91	20.24 ± 0.80^bbb^

Enzymatic calorimetric lipid profiling of plasma and liver tissue isolated from wild type (wt), Epac1^−/−^, Epac2^−/−^ and Epac1/2^−/−^ control mice and PH operated mice 36 h post surgery. Data are presented as mean ± SD, n = 3–10 mice/group. Two-way ANOVA with Bonferroni’s adjustment for multiple comparisons or the non-parametric Kruskal-Wallis test with Dunn’s multiple comparison test was used to determine differences between genotypes and treatment groups for each factor. ^a^p < 0.05, ^aa^p < 0.01, ^aaa^p < 0.001 and ^aaaa^p < 0.0001: control wt mice compared to control Epac1^−/−^, Epac2^−/−^ and Epac1/2^−/−^ mice. ^bb^p < 0.01, ^bbb^p < 0.001 and ^bbbb^p^ < ^0.0001: control mice compared to PH operated mice 36 h post surgery, same genotype. ^c^p < 0.05, ^ccc^p < 0.001 and ^cccc^p < 0.0001: PH operated Epac1/2^−/−^ mice 36 h post surgery compared to PH operated wt, Epac1^−/−^ and Epac2^−/−^ mice 36 h post surgery. ^dd^p < 0.01: control Epac1/2^−/−^ mice compared to control wt, Epac1^−/−^ and Epac2^−/−^ mice. F-statistics for lipid profiling in plasma; C; F(3,42) = 5.597, p = 0.0025, HDL-C; F(3,41) = 5.777, p = 0.0022, LDL-C; F(3,40) = 4.116, p = 0.0123, UC; F(3,42) = 0.2445, p = 0.8647, TG; F(3,42) = 3.995, p = 0.0137, NEFA; F(3,40) = 3.423, p = 0.0261 and PL; F(3,41) = 2.368, p = 0.0847. F-statistics for lipid profiling in liver; C; F(3,42) = 1.583, p = 0.2078, TG; approximate p < 0.0001, Kruskal-Wallis statistic; 31.21, NEFA; approximate p = 0.0018, Kruskal-Wallis statistic; 22.85 and PL; F(3,42) = 1.210, p = 0.3177. **C**: Cholesterol, **HDL-C**: High-density lipoprotein-cholesterol, **LDL-C**: Low-density lipoprotein-cholesterol, **UC**: unesterified cholesterol, **TG**: Triglycerides, **NEFA**: Non-esterified fatty acids and **PL**: phospholipids.

The hepatic lipid profiles were nearly identical in the different genotypes in mice not subjected to PH (Table 1). The only variation found was that Epac1^−/−^ mice had lower levels of phospholipids compared to Epac1/2^−/−^. However, taken together, these results indicate that under resting conditions, lipid metabolism is adequately maintained in the absence of Epac1/2. In response to PH, increased hepatic levels of TGs and cholesterol were observed in wt mice as reported^35^. Similarly, cholesterol was significantly increased in Epac1^−/−^ ad Epac2^−/−^ mice (Table 1). TG levels were also elevated in these genotypes, but due to large variations between individuals within the same genotype (Epac1^−/−^ and Epac2^−/−^), the differences where not statistically significant. As also evident from the ORO staining (Fig. 4), Epac1/2^−/−^ mice exhibited lower levels of TGs in the livers post-PH, but the large individual variations within each genotype resulted in non-significant statistical results. In contrast to the other genotypes, hepatic cholesterol was not increased in Epac1/2^−/−^ mice after PH, also contributing to an overall reduced mass of neutral lipids in this group.

**Figure 5:**
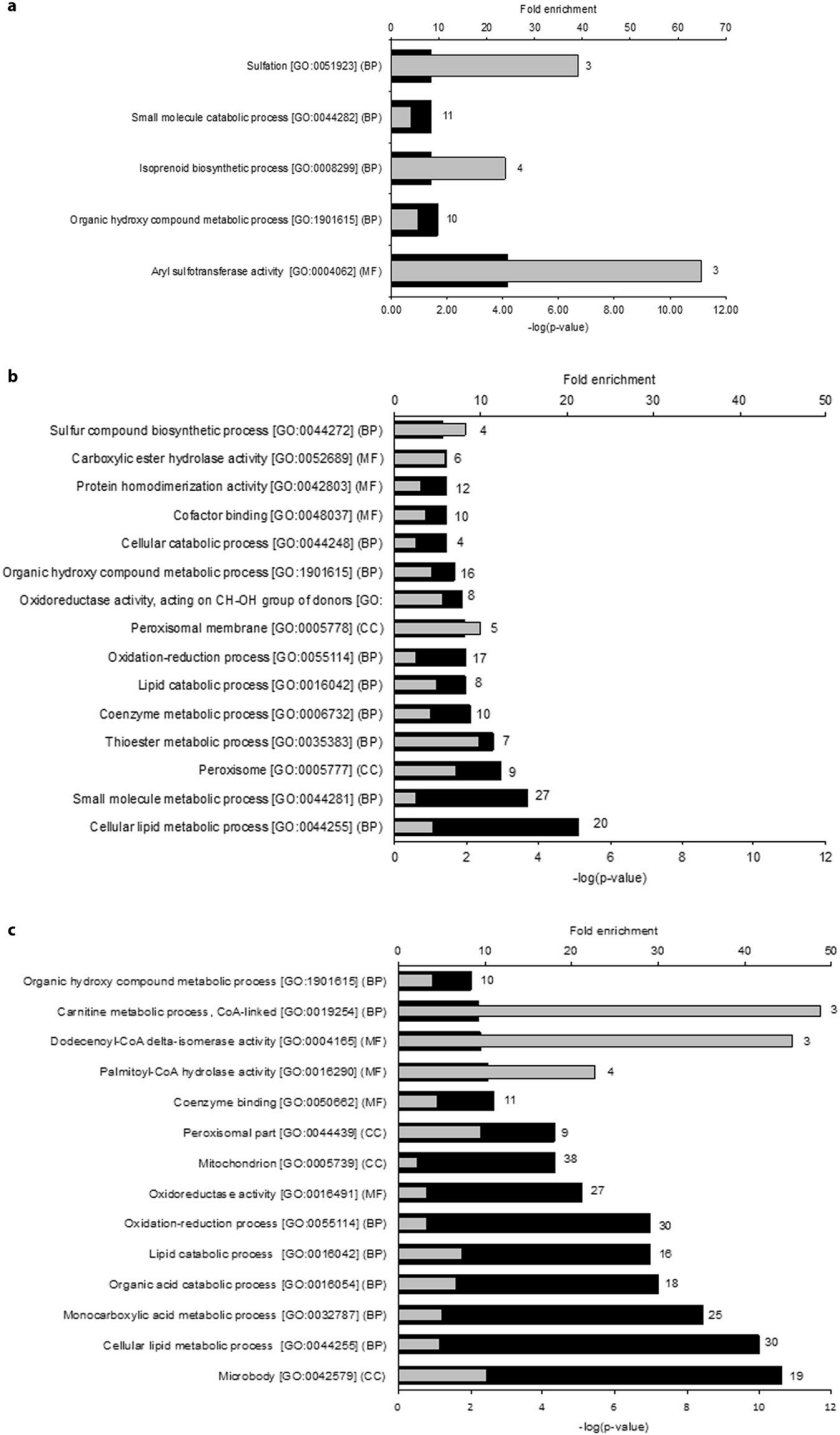
Functional annotation of differentially expressed proteins. GO enrichment analyses on proteins that were differentially expressed in wt and Epac1/2^−/−^ mice (**a/b**) pre- or (**c**) 36 h post-PH. (**a)** Functional annotation of proteins with significantly higher expression pre-PH in Epac1/2^−/−^ mice compared to wt. (**b**) Functional annotation of proteins with significantly lower expression pre-PH in Epac1/2^−/−^ mice compared to wt. (**c**) Functional annotation of proteins with significantly lower expression post-PH in Epac1/2^−/−^ mice compared to wt. Black bars indicate the respective p-values and grey bars indicate fold enrichment for the corresponding GO-term. The number of proteins annotated with each GO-term is shown behind each bar. BP: biological processes; MF: molecular function (MF); CC: cellular compartment.

The dramatic effect on lipid accumulation in Epac1/2^−/−^ mice 36 h after PH, but milder effects in mice deleted for either Epac1 or Epac2 (Fig. 4 and Table 1), provoked further analyses of the double knockout model. Proteomic analyses of liver tissue harvested pre-PH identified 113 proteins that were differentially expressed in Epac1/2^−/−^ mice compared to wt mice (54 increased and 59 decreased). Gene ontology (GO) enrichment analyses of the proteins with increased expression pre-PH in Epac1/2^−/−^ mice relative to wt mice indicated over-representation of proteins connected to sulfation and sulfotransferase activity, and biosynthetic processes related to, among other molecules, cholesterol biosynthesis and steroid metabolism (Fig. 5a; see Supplementary Table 1a for an overview of the proteins identified for each GO-term). The proteins with reduced expression in Epac1/2^−/−^ mice compared to wt mice pre-PH were mainly associated with lipid metabolism, peroxisome biology and oxidation-reduction processes (Fig. 5b, and Supplementary Table 1b). Post-PH, again 113 proteins were differentially expressed in Epac1/2^−/−^ mice compared to wt mice (38 increased and 75 decreased). GO enrichment analyses of the proteins with decreased expression post-PH predicted enrichment of similar or related pathways and processes as before PH, but interestingly, the statistical prediction was considerably strengthened for the terms related to peroxisome biology (Fig. 5c; see Supplementary Table 2 for an overview of the proteins identified for each GO-term). There were no significantly enriched GO-terms for the proteins that were increased in Epac1/2^−/−^ mice compared to wt mice post-PH. In accordance with previous studies^36^, PH altered the hepatic protein composition in wt mice, resulting in enrichment of GO terms related to metabolic processes, mainly lipid metabolism, the inflammatory response and regeneration (Supplementary Fig. S6). The expression of proteins linked to lipid metabolism was also changed in Epac1/2^−/−^ post-PH, and interestingly, the GO analyses predicted enrichment for the terms “Negative regulation of gluconeogenesis” and “Very-low density lipoprotein particle” in the absence of Epac1/2 (Supplementary Fig. S6).

**Figure 6:**
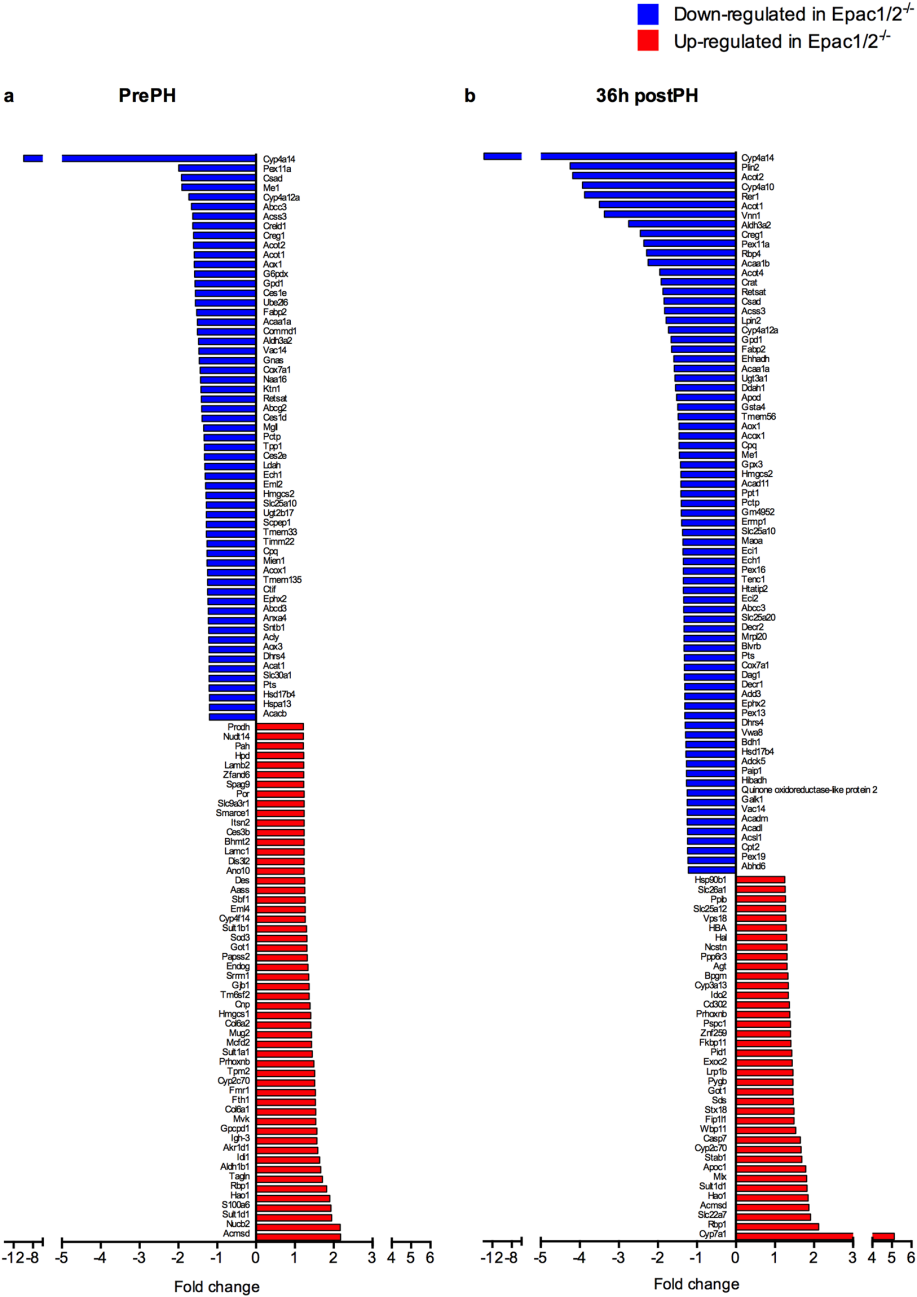
Differentially expressed proteins in Epac1/2^*−/−*^ mice compared to wt. The proteins that were differently expressed in Epac1/2^−/−^ mice compared to wt mice (**a**) pre- and (**b**) post-PH are listed. Blue bars indicate proteins that were decreased in Epac1/2^−/−^ mice compared to wt mice, and red bars indicate proteins that were increased in Epac1/2^−/−^ mice compared to wt mice. Statistics: two-tailed student’s two-sample *t*-test and Z-statistics for FC, only when p < 0.05 for both it was considered significant.

**Figure 7:**
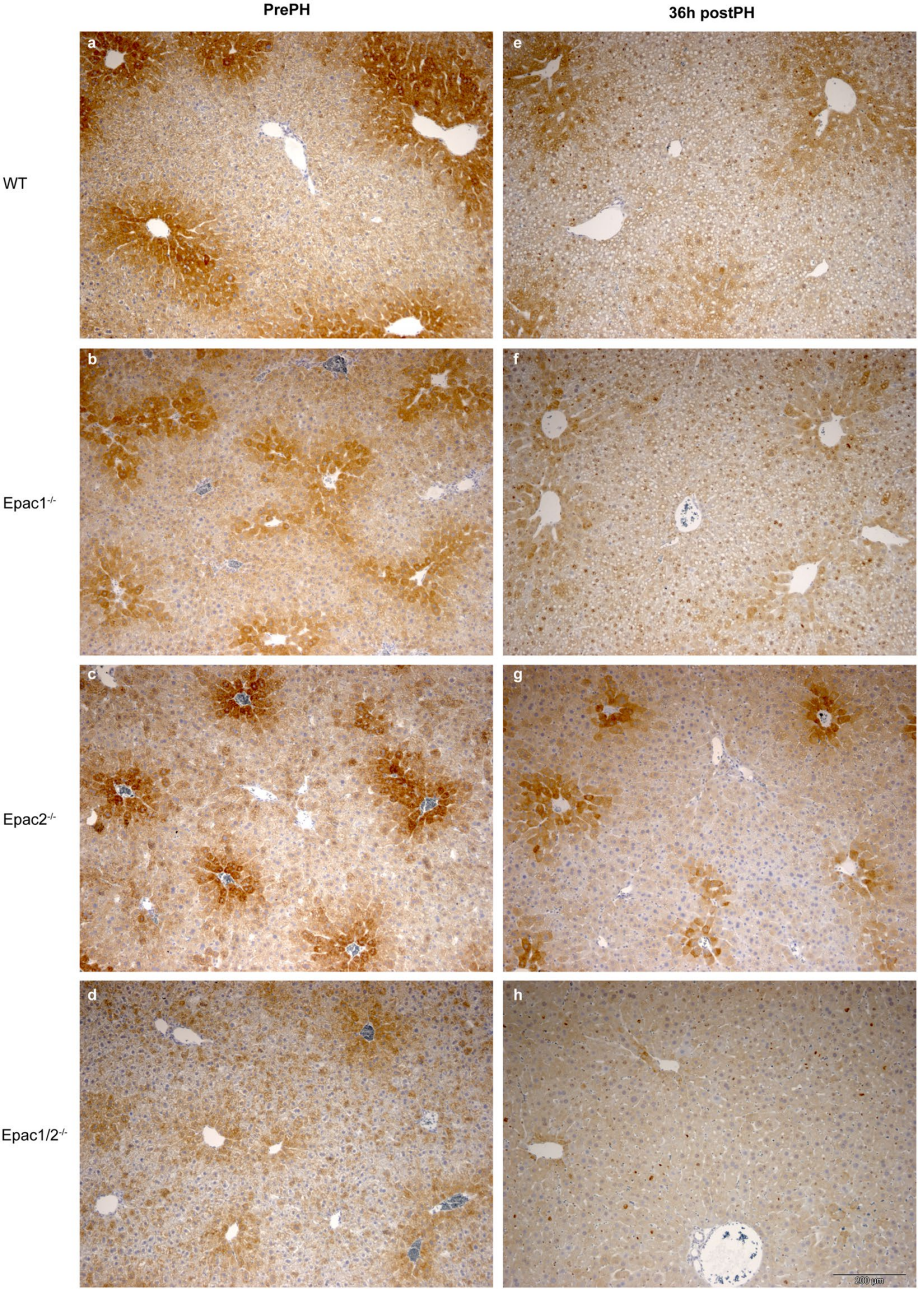
Decreased expression of Cyp4A14 in Epac1/2^*−/−*^ deficient mice. IHC analyses (DAB staining) to determine Cyp4a14 expression in liver sections from wt, Epac1^−/−^, Epac2^−/−^ and Epac1/2^−/−^ mice pre-PH **(a–d)** and 36 h post-PH **(e–h)**. The antibody used also detects Cyp4a10 and Cyp4a12. 4–6 mice in each experimental group were analyzed (3 sections from each animal). Representative images are shown. Bar: 200 μm.

Of particular interest is that the expression of cytochrome P4504A14 hydroxylase (Cyp4a14) was severely affected in Epac1/2^−/−^ mice. This enzyme catalyzes omega-hydroxylation of medium-chain fatty acids, and is implicated in hepatic steatosis and fibrosis^37^. The proteomic analyses demonstrated a 10 and 14 fold repression of Cyp4a14 in Epac1/2^−/−^ mice compared to wt mice before and after PH, respectively (Fig. 6a,b). IHC and immunoblotting analyses verified the decreased expression of Cyp4A14 in mice deleted for Epac1, Epac2 or Epac1/2 (Fig. 7 and Supplementary Fig. S7; see Supplementary Fig. S8 for full-length blots). The antibody used in IHC and immunoblotting recognizes Cyp4a10 and Cyp4a12, in addition to Cyp4a14. Thus, the IHC and immunoblotting results do not perfectly mimic the reduction in Cyp4A14 expression observed in the proteomic analyses. Cyp4a12 was downregulated 1.7 fold in Epac1/2^−/−^ mice both before and after hepatectomy (Fig. 6), and Cyp4a10 was downregulated 2 and 4 fold in Epac1/2^−/−^ mice pre- and post-PH, respectively (Fig. 6). The IHC analyses demonstrated that in wt mice, the highest expression of Cyp4A14 was in the centrilobular region^38^. Epac1^−/−^, Epac2^−/−^ and Epac1/2^−/−^ mice exhibited a distribution pattern with fewer centrilobular Cyp4A14 positive cells, a weaker staining intensity and a localization that included Cyp4A14 positive cells in the transition- and periportal regions pre-PH (Fig. 7).

Cyp4a14 is a target gene for peroxisome proliferator-activated receptor alpha (PPARα)^39^, and it is notable that a considerable number of the proteins that were differentially expressed in Epac1/2^−/−^ mice compared to wt mice (both pre- and post-PH) are known PPARα target genes. In particular, enzymes involved in fatty acid degradation were generally decreased in Epac1/2^−/−^ mice as opposed to wt mice (Table 2). However, immunoblotting analyses demonstrated no effect on PPARα expression as a consequence of Epac1/2 deletion (Supplementary Fig. S9; see Supplementary Fig. S10 for full-length blots) and the proteomic analysis did not provide insight into a potential effect of PPARα expression in Epac1/2^−/−^ mice, as we were unable to detect PPARα in this analysis. Cytochrome P4507α hydroxylase (Cyp7α1), was the protein found to be most significantly increased in Epac1/2^−/−^ mice post-PH compared to before surgery (5-fold) (Fig. 6b). This was in sharp contrast to wt mice where the expression of CYP7α1 was decreased in response to PH, in accordance with the literature^40^ (Supplementary dataset file 2). Cyp7α1 is also a PPARα target gene^39,41^, and catalyzes the first reaction in the bile acid biosynthetic pathway^42^. The expression of other enzymes involved in this process (*i.e*. Akr1d1: Aldo-keto reductase family 1 member D1 and HSD17b4: Hydroxysteroid 17-beta dehydrogenase 4) was also altered in Epac1/2^−/−^ mice. Of note is that the expression levels of the different PKA subunits (i.e. Prkar1a Prkar2a, Prkaca and Prkacb) were not changed in Epac1/2^−/−^ mice (Supplementary dataset file 2), and neither were the expression of the different isoforms of the Epac1/2 downstream targets Ras-related protein Rap1/2 (Rap1a, Rap1b, Rap2a:Rap2c;Rap2b). Recently, several studied have shown that Epac1/2 is localized to the mitochondria^43–46^. In this regard, it is interesting to note that a substantial number of the proteins with decreased expression in Epac1/2^−/−^ mice that are associated with lipid metabolism and oxidation-reduction processes, are mitochondrial proteins (Supplementary Table S3).

**Table 2:** List of PPARα target genes that are less expressed in Epac1/2^−/−^ mice.

ID	Gene symbol	Protein name	prePH FC	postPH FC
P55096	Abcd3	ATP-binding cassette sub-family D member 3	−1.23	−1.19, ns
Q921H8	Acaa1a	3-ketoacyl-CoA thiolase A, peroxisomal	−1.51	−1.58
Q8VCH0	Acaa1b	3-ketoacyl-CoA thiolase B, peroxisomal	−1.64, ns	−2.25
P51174	Acadl	Long-chain specific acyl-CoA dehydrogenase, mitochondrial	−1.11, ns	−1.24
P45952	Acadm	Medium-chain specific acyl-CoA dehydrogenase, mitochondrial	−1.08, ns	−1.25
Q9QYR9	Acot2	Acyl-coenzyme A thioesterase 2, mitochondrial	−1.61	−4.18
Q8BWN8	Acot4	Acyl-coenzyme A thioesterase 4	−1.78, ns	−1.95
Q9R0H0	Acox1	Peroxisomal acyl-coenzyme A oxidase 1	−1.25	−1.46
P47740	Aldh3a2	Fatty aldehyde dehydrogenase	−1.48, ns	−2.75
P52825	Cpt2	Carnitine O-palmitoyltransferase 2, mitochondrial	−1.02, ns	−1.24
P47934	Crat	Carnitine O-acetyltransferase	−1.59, ns	−1.92
O88833	Cyp4a10	Cytochrome P450 4A10	−2.01, ns	−3.93
Q91WL5	Cyp4a12a	Cytochrome P450 4A12A	−1.73	−1.73
O35728	Cyp4a14	Cytochrome P450 4A14	−9.89	−13.73
Q9CQ62	Decr1	2,4-dienoyl-CoA reductase, mitochondrial	−1.19, ns	−1.31
O35459	Ech1	Delta(3,5)-Delta(2,4)-dienoyl-CoA isomerase, mitochondrial	−1.31	−1.35
Q9DBM2	Ehhadh	Peroxisomal bifunctional enzyme; Enoyl-CoA hydratase/3,2-trans-enoyl-CoA isomerase; 3-hydroxyacyl-CoA dehydrogenase	−1.18, ns	−1.59
P51660	Hsd17b4	Peroxisomal multifunctional enzyme type 2;(3 R)-hydroxyacyl-CoA dehydrogenase; Enoyl-CoA hydratase 2	−1.20	−1.28
Q9Z211	Pex11a	Peroxisomal membrane protein 11A	−1.99	−2.36
Q9Z2Z6	Slc25a20	Mitochondrial carnitine/acylcarnitine carrier protein	−1.01, ns	−1.33

PrePH (WTprePH *vs*. Epac1/2^−/−^prePH), postPH (WTpostPH *vs*. Epac1/2^−/−^postPH), negative value: decreased protein expression in Epac1/2^−/−^ mice relative to wt mice. ns: not significant. FC: fold change. Please see the Supplementary M&M section for details regarding statistical analysis.

### The number of KCs is affected in Epac1^−/−^ and Epac2^−/−^ mice

**Figure 8:**
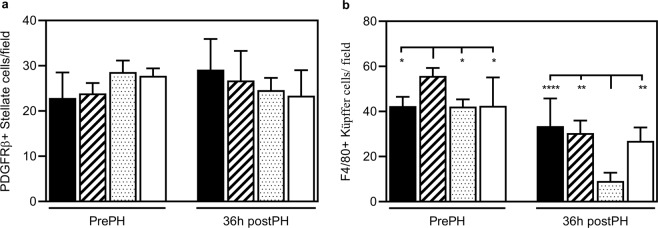
Altered KC number in Epac1^*−/−*^ and Epac2^*−/−*^ mice. The number of (**a)** PDGFβ-positive stellate cells and (**b)** F4/80-positive KCs was determined in the livers of wt, Epac1^−/−^, Epac2^−/−^ and Epac1/2^−/−^ mice pre- and 36 h post-PH. Two-way ANOVA with Tukey’s adjustment for multiple comparisons was used to determine statistical differences between genotypes within each time point. n = 4–6 mice per group, *p < 0.05, **p < 0.01, and ****p < 0.0001. F-statistics: (**a**) F(3,16) = 2.670, p = 0.0836 and (**b**) F(3,15) = 6.630, p = 0.0045.

It is intriguing that deletion of either Epac1 or Epac2 alone causes phenotypes in response to PH, and that some of the effects are reinforced when both factors are deleted. These findings suggest that also Epac1 expressing cells, and not only hepatocytes, are affected in the livers of the knockout models. To gain further insights into the potential effects on nonparenchymal cells, we quantified the relative numbers of KCs and HSCs by IHC. Both cell types are known to exert stimulatory and inhibitory effects on hepatocyte proliferation following PH^47,48^. We observed no significant differences between genotypes with regard to the number of platelet derived growth factor beta (PDGFβ) - positive HSCs (Fig. 8a). However, the number of F4/80-positive KCs was increased in Epac1^−/−^ mice pre-PH, and decreased in Epac2^−/−^ mice 36 h post-PH (Fig. 8b).

## Discussion

The mammalian response to PH has been extensively studied, but the details of how the signaling cascades that control liver regeneration integrate, remain incompletely understood. It is known that cAMP and PKA are involved, and the production of cAMP is tightly controlled during the process^49–52^. Moreover, a delay in regeneration is observed in mice deleted for the cAMP-regulated transcription factor CREM (cAMP responsive element modulator)^3^. In the present study, we demonstrate for the first time that the cAMP binding proteins Epac1/2 are involved in regulating liver regeneration in an *in vivo* model. Deletion of Epac1 or Epac2, or both factors together, caused phenotypes manifested in increased lipid turnover and DNA synthesis early in the regenerative process. Moreover, we found that expression of Epac2C was upregulated in the regenerating liver, that the number of KCs cells was altered and that the liver proteome of Epac1/2^−/−^ mice differed from wt mice, a disparity that was substantiated in response to PH. Taken together, it appears that loss of Epac1/2 is compatible with normal liver functions in mice living in a protected environment, but that these factors become important in the regulation of cellular responses in stressful situations. We used global knockout models in this study, and cannot disregard possible indirect effects of Epac1/2 deletion on liver regeneration, and thus, future analyses to decipher the molecular roles of Epac1 and Epac2 in different hepatic cell lines should include cell-specific knockout models. An example of a possible indirect effect is via blood platelets. It is clear that platelets and platelet-derived factors have central roles in liver regeneration after PH^53–55^, and in this regard it is interesting to note that we previously demonstrated that Epac1^−/−^ mice (but not Epac2^−/−^ mice) display a bleeding phenotype that is partly caused by altered platelet maturation and function^56^.

The fact that some of the observed phenotypic traits were substantiated in Epac1/2^−/−^ mice compared to single knockouts, indicates that these proteins have distinct, but convergent roles in the regenerative process, and that lack of one factor (and both) causes defective cell communication and integration of signals. This notion is supported by the cell specific and mutual exclusive expression of Epac1 and Epac2 in the liver, and by the finding that the number of Epac1 expressing KCs is altered not only in Epac1^−/−^ mice, but also in Epac2^−/−^ mice post-PH. Epac1 exerts anti-inflammatory effects in different biological systems^57^, and at present, it is unclear whether the increased number of F4/80-positive KCs observed in Epac1^−/−^ mice results from increased recruitment of circulating macrophages due to an increased inflammatory state in the absence of Epac1, or caused by intrinsic effects of the liver. Interestingly, the pronounced increase in TNFα that we observe in Epac1^−/−^ mice before PH is in agreement with an enhanced recruitment of circulating monocyte-derived macrophages, which is known to secrete pro-inflammatory cytokines, whereas the pro-regenerative liver resident KCs secrete anti-inflammatory cytokines^58^.

The discovery that the number of KCs is changed in Epac2^−/−^ mice 36 h post-PH might imply that the interaction between hepatocytes and KCs is disturbed. We have limited knowledge of how hepatocyte mediated signaling affects KC replenishment post-PH. However, it has been suggested that dysregulated secretion of granulocyte-macrophage colony-stimulating factor (GM-CSF) from hepatocytes interferes with, or delays KC replenishment post-PH^59,60^. This is interesting considering the suggested role for GM-CSF in hepatic lipid homeostasis^61^, and growing evidence demonstrating that KC renewal is affected by the metabolic state of the liver^62^. It is yet to be determined whether GM-CSF mediated signaling is affected in the absence of Epac2C.

Rap1 GTPase, the main downstream Epac1/2 effector, plays an important role in regulating cell proliferation. However, the roles of Epac1/2 in cell proliferation have remained elusive, as both pro-mitotic and anti-mitotic effects have been ascribed to these proteins depending on cellular context^63^. The increased BrdU incorporation observed in Epac1^−/−^, Epac2^−/−^ and Epac1/2^−/−^ mice at 36 h post-PH in the present study, indicates that the absence of Epac1/2 causes an accelerated first round of hepatocyte proliferation. In mice, coordinated waves of hepatocyte proliferation occur in response to PH, with the first round being the most robust^64^. The expression of cell cycle regulators are expressed within hours after PH, and interestingly, it has been demonstrated that the relative expression of the different cell cycle components is rather consistent throughout the liver regrowth, despite fluctuations in DNA synthesis during the process^65^. In line with this, although we detected increased proliferation at 36 h post-PH, we did not observe increased expression of Cyclin D1, Cyclin E1or cyclin dependent kinases (Cdks) in mice deleted for Epac1/2. However, we can not rule out that the levels of these factors differed between wt and Epac1/2 knockout models at time points earlier than 26 h and therefore escaped detection in our experimental set-up. We found that the expression of TNFα is lower in Epac1/2^−/−^ mice, and partly in Epac2^−/−^ mice compared to wt mice at the peak of DNA-synthesis (36 h post-PH). TNFα triggers the priming phase in hepatocyte proliferation after PH, whereas this factor is suppressed at later stages^66^. Thus, the observed decreased expression of TNFα in Epac1/2^−/−^ mice substantiates the idea that hepatocyte proliferation occurs more rapidly in mice lacking Epac1/2. Taken together, we suggest that the increased number of BrdU-positive hepatocytes in Epac1^−/−^, Epac2^−/−^ and Epac1/2^−/−^ mice 36 h post-PH, demonstrate that the Epac proteins restrain hepatic cell proliferation under normal conditions.

A hallmark of the early stages of liver regeneration in response to PH is the accumulation of lipids, which contributes a greater portion of the energy substrates necessary to complete regrowth^67^. The finding that Epac1/2^−/−^ mice are completely devoid of lipid droplets 36 h post-PH indicates regulatory roles for Epac1/2 in this process in wt mice. Despite large individual differences, the ORO-staining at 26 h points to the fact that Epac1/2^−/−^ mice were capable of accumulating lipids at early time points after PH. We propose that ablation of Epac1/2 causes a shift in the regeneration process with a more rapid lipid turnover accompanied with increased hepatocyte proliferation. These irregularities did affect neither the overall regeneration process nor the survival of the animal, indicating that the role of Epac1/2 is to balance the regulatory mechanisms that control the process. This is consistent with several publications demonstrating that normal lipid accumulation in response to PH is not a prerequisite for normal regeneration^34,68^.

The proteomic analyses presented in this study disclosed that the expression of Cyp4A14 is dramatically decreased in Epac1/2^−/−^ mice, both pre- and post-PH. This enzyme, and the human homolog (CYP4A hydroxylase), catalyze omega-hydroxylation of medium chain fatty acids and arachidonic acids^69^. Interestingly, Cyp4A14 expression is increased in murine models of spontaneous nonalcoholic fatty liver disease (NAFLD)^70–72^, as well as in livers of patients with NAFLD^37^. Moreover, overexpression of, or genetic ablation of Cyp4A14 in mice correlates with increased and reduced hepatic fat accumulation, respectively, in response to a high fat diet^37^. The result that Cyp4A14 is strongly repressed in livers from Epac1/2^−/−^ mice both before and after hepatectomy provides a link between Epac1/2, Cyp4a14 and the regulation of hepatic lipid turnover. NAFLD is displayed as a range of diseases that can develop to non-alcoholic steatohepatitis and further to fibrosis^73^. Cyp4a14 also plays a role in the development of fibrosis, as diet induced portal and perisinusoidal fibrosis is attenuated in mice depleted for Cyp4A14 expression, as is the expression of fibrosis-related genes in HSC (activation of HSC is central in the development of hepatic fibrosis)^37^. In this context it is of interest that Epac1 has been proposed to exhibit anti-fibrotic functions, and that several studies point to Epac1 as an integrator of pro- and anti-fibrotic signals in HSC^13,17,18^. Whereas the pro-fibrogenic factor TGFβ1 decreases Epac1 expression in HSC, overexpression of Epac1 inhibits TGFβ1-induced production of collagen^13^. Expression of Epac1 is also repressed in fibrotic livers of mouse and human, and stimulation of Epac1 expression in HSC partly restores carbon tetrachloride induced fibrosis in mice^17^. The effects of high fat feeding in mice deleted for Epac1/2 is not yet clear, as conflicting results are obtained from different Epac knockout models^23,33,74^. A recent study describes increased obesity and fat accumulation in adipose tissue and liver in male mice deleted for Epac2A^74^. However, since the model used in this study^74^ expresses normal levels of Epac2C in hepatocytes, it does not provide information about the intrinsic roles for Epac2C in the liver. On the other hand, it does imply that Epac2A, acting outside of the liver, affects hepatic lipid accumulation indirectly, prompting future studies on hepatocyte specific Epac2C knockouts^69^.

Several mouse models that lack expression of Epac1/2 have been developed^23,33,75–78^. A common feature of these models is that deletion of either Epac1 or Epac2, or both, does not produce gross anatomical or physiological abnormalities, but that exposure to stress provokes phenotypes in a diversity of physiological processes, as we describe herein for the liver^23,33,56,75,76,78–83^. Collectively, these studies suggest that Epac1/2 may have evolved to balance cellular responses in challenging situations, and implicate Epac1/2 in a number of diseases. Epac1/2 are considered promising drug targets, and Epac2 has been suggested to be the target for both incretin-related drugs and sulfonylureas, which are widely used to treat type 2 diabetes^84^. Currently, considerable effort is invested in developing compounds that specifically act on either Epac1 or Epac2 to tailor treatment and reduce side effects^26,85^. The roles of Epac1/2 in liver physiology disclosed in the present study hold promise for that compounds acting via Epac1/2 may be used in the future to treat liver diseases.

## Materials and Methods

### Animals

Male mice (C57BL/6J background, 8–12 weeks) were used in this study. Epac1^−/−^ and Epac2^−/−^ mice were generated using the Cre-*Lox*P targeting strategy described elsewhere^56,81–83^. Of note, the targeting strategy used ablates the expression of all Epac2 isoforms (i.e. Epac2A, Epc2B and Epac2C). Mice deleted for Epac1 and Epac2 (Epac1/2^−/−^) were generated by crossing of Epac1^−/−^ and Epac2^−/−^ mice. Wt mice used in the study (originally from Taconic, Denmark) were littermates of Epac1^−/−^ and Epac2^−/−^ mice or, mice bred in the same room. The origin of the C57BL/*6*JBomTac mice is described at https://www.taconic.com/mouse-model/b6jbom. The mice were housed in groups of three to five and bred at the animal facility at Haukeland University Hospital and maintained under standard conditions with a temperature of 21 °C ± 0.5 °C, humidity of 55% ± 5%, lighting of 150 lux and 12 hours light/dark cycle (7 am/7 pm). Animals were housed in IVC II cages with food (RMI (E)) and water *ab libtium*. All animal protocols were approved by the Norwegian Animal Research Authority and performed according to the European Convention for the Protection of Vertebrates Used for Scientific Purposes. The Animal Care and Use Programs at University of Bergen are accredited by AAALAC international.

### Hepatectomy

2/3 partial hepatectomy (PH) or sham laparotomy was performed as described^86^. At the selected post-operative endpoints (26 h, 36 h, 74 h and 168 h), the mice were euthanized by CO_2_- suffocation, with administration of BrdU (50 mg/kg IP) 2 h before sacrifice. Tissue removed at time of surgery (left lateral and median lobes) and regenerated tissue (right lateral and caudate lobes) removed at selected endpoints were flash-frozen on liquid nitrogen, or fixed in 4% paraformaldehyde (PFA; 10x w/v) for 18–24 h, embedded in paraffin and sectioned (4 μm). Trunk blood was obtained by cardiac puncture, collected into prechilled EDTA-tubes and blood plasma prepared. See Supplementary S1 Materials & Methods, for details on H&E, ORO staining, hepatocyte size quantification, lipid profiling, immunohistochemistry and immunoblotting.

### BrDU analyses

Hepatocyte proliferation was assessed by monitoring BrdU-incorporation on paraffin sections according to manufacturer’s protocol (Anti-BrdU antibody (BU1/75 (ICR1), ab6326, Abcam)), with some modifications: Briefly; paraffin sections were dewaxed and DNA denaturized in HCl (2 N) for 30 min at 37 °C followed by neutralization in borate buffer (0.1 M) for 2 × 5 min at RT. Next, sections were rinsed 3 × 5 min in TBS, incubated in blocking buffer (10% normal donkey serum/2xTBS-T) for 60 min at RT and incubated with Anti-BrdU antibody (1:300 in 3% normal donkey serum/TBS-T) at 4 °C o/n with gentle agitation. Parallel sections used as negative controls were incubated with Rat IgG2a Isotype Control (1:300 in TBS-T) or in blocking buffer o/n at 4 °C. Next, sections were rinsed for 4 × 10 min in 2xTBS and incubated with Alexa Fluor^®^ 488 Donkey Anti-Rat IgG (1:200 in 3% normal donkey serum/TBS-T) for 1.5 h at RT. Lastly, sections were rinsed 4 × 10 min in TBS, counterstained and mounted with Prolong® Gold Antifade Reagent with DAPI and coverslipped. DAPI-stained sections were not blocked, but directly counterstained and mounted. The stained sections were visualized with Zeiss Axioplan2 microscope and AxioVision version 4.5 Program (Zeiss), hepatocyte proliferation was determined by manually counting positive BrdU nuclear staining. For each mouse, ten 40x microscopic fields (in an area of 35.2 mm^2^) in three different sections were counted blindfolded by two different researchers. Likewise, DAPI staining was counted manually in ten 40x microscopic fields in one section from each mouse.

### Real time (RT) and quantitative (q) PCR

#### Primary cell isolation and fluorescence-activated cell sorting

Hepatocytes and HSC were isolated as described previously^8,87^. Liver resident macrophages (KCs) were extracted as described previously^88^. In brief, mouse liver was perfused through the inferior vena cava with PBS. Livers were excised, minced and digested in solution I (0.8 mg/ml collagenase V, 0.625 mg/ml collagenase D, 1 mg/ml Dispase and 30 μg/ml DNase). The suspension was shaken on an orbital shaker at 37 °C for 25 min, with additional vigorous manual shaking every 5 min. Digest was strained through a 100 μm cell strainer. Following 2 × 5 min centrifugation at 300 g RBC lysis buffer was added for 3 min to lyse red blood cells. Cells were then centrifuged at 300 g for 5 min, resuspended in FACS buffer (2% FBS (Gibco) and 2 mM EDTA in Dulbecco’s phosphate-buffered saline no calcium, no magnesium (Gibco)) and filtered through a 35 μm strainer. Cells were blocked with 10% mouse serum (Sigma) containing 1% Purified anti-mouse CD16/32 (Biolegend) for 10 min. Cells were then incubated with fluorescent conjugated antibodies (FITC anti-mouse CD45.2, PE/Cy7 anti-mouse F4/80, APC/Cy7 anti-mouse/human CD11b, PE anti-mouse CD3, PE anti-mouse CD19, PE anti-mouse Ly-6G, APC anti-mouse CD31 and PE Rat Anti-Mouse Siglec-F) or isotype control at 4 °C for 30 min in the dark. Cells were washed, resuspended in FACS buffer and sorted using a FACS Aria II (BD Biosciences, USA). Following a positive CD45.2 gate and dump of CD3, CD19, Ly-6G, Siglec-F, and CD31 positive KCs were defined as the F4/80 high, CD11b intermediate population.

To isolate liver sinusoidal endothelial cells (LSEC), mouse liver was perfused through the inferior vena cava with PBS. Livers were excised, minced and digested in a solution containing 5 mg/ml collagenase I and 11μg/ml DNase. This cell suspension was shaken at 37 °C for 20 min. Digest was strained through a 70 μm cell strainer. Following 7 min centrifugation at 400 g, RBC Lysis buffer was added for 5 min. Cells were then centrifuged at 400 g for 7 min, resuspended in FACS buffer and filtered through a 35 μm strainer. Cells were blocked with 10% mouse serum and 1% CD16/32 for 10 min. Cells were then incubated with fluorescent conjugated antibodies (PE/Cy7 anti-mouse CD45 and APC anti-mouse CD31 clone. 390) or isotype control at 4 °C for 30 min in the dark. Cells were washed, resuspended in FACS buffer and sorted using a FACS Aria II. Endothelial cells were sorted from the CD31 positive, CD45 negative population.

RNA was extracted from the isolated cells using the RNeasy Micro kit (74004, Qiagen, Germany), and converted to cDNA using the SuperScript® III First-Strand Synthesis SuperMix for qRT-PCR kit (Invitrogen, Waltham, MA, USA). The RT-PCR reaction was carried out as described in^8^. For qPCR analyses, RNA was prepared from total liver and converted to cDNA using the GenElute Mammalian Total RNA miniprep Kit, and the iScript™ cDNA Synthesis kit (BioRad). qPCR was carried out on a LightCycler 480 II (Roche) using SYBR Green Supermix (BioRad) with following amendments in the thermal cycling protocol: 1 cycle for 300 sec at 95 °C and 40 cycles for 10 sec at 95 °C, 10 sec at 59 °C and 10 sec at 72 °C followed by a melting curve analysis. All samples were run in triplicates and repeated in three separate experiments. Expression levels of Epac1 and Epac2 Universal were normalized to the geometrical mean of SDHA and Ppib, and relative gene expression determined using the 2^−ΔΔCt^-method^89–91^. Primer sequences: Epac1 fwd: GAAAATGGCTGTGGGAACGTATCT, Epac1 rev: AGCTGCTCAGGGTGTGGGGT, Epac2 Universal fwd: ATCTACGAGACGAGCT CCTTCATATTAAA, Epac2 Universal rev: GACTACATTCACGGATCCTTTCAGA, β-Actin fwd: GGCCCAGAGCAAGAGAGGTATC, β-Actin rev: AGGCATACAGG GACAGCACAGC, Ppib fwd: GGAGATGGCACAGGAGGAAA, Ppib rev: CGTAG TGCTTCAGCTTGAAGTTCT, SDHA fwd: CATGCCAGGGAAGATTACAA and SDHA rev: GCACAGTCAGCCTCATTCAA.

### Proteomic analyses

Frozen liver samples (10–12 µg) harvested pre- and 36 h post-PH were lysed in SDS lysis buffer (4% SDS in 0.1 M Tris-HCl, pH 7.6) and processed for LC-MS analysis according to the filter-aided sample preparation (FASP) procedure^92^, as described^93^. NanoLC mass spectrometry and proteomic data analyses were performed as described in Supplementary Materials & Methods. Proteins with significant differential expression were classified according to their biological processes using Gene ontology (GO) enrichment analysis with the online resource A.GO.TOOL software (University of Copenhagen, Denmark)^94^.

### Statistics

Statistical analysis was performed using GraphPad Prism 6.0. The One-way ANOVA with Dunnett’s or Bonferroni’s adjustment for multiple comparisons (when applicable) or the Two-way ANOVA with Bonferroni’s adjustment for multiple comparisons (when applicable) was used to assess statistical differences between groups. The Brow-Forsynthe test was used to test the normality of the data. If the data did not meet the requirements for normal distribution, the non-parametric Kruskal-Wallis test with Dunn’s multiple comparison was used to assess statistical differences between groups. The data are shown as median with and min to max whiskers for box plots and mean ± SD for others. The degrees of freedom, F- and p-values for the different pair-wise comparisons are also provided in the figure legends. p < 0.05 was considered statistically significant. Proteomics: See Supplementary Materials & Methods.

## Supplementary information

Supplementary Information

Supplementary file 2 proteomic data

## Data Availability

The proteomics raw files and data were uploaded to the ProteomeXchange consortium (version 2.3.0) via the PRIDE partner repository, and with the dataset identifier PXD006165^95,96^. A detailed description of the LC-MS analysis is provided in the Supplementary Material & Methods section.
