# Supplementary material for: Mice depleted for Exchange Proteins Directly Activated by cAMP (Epac) exhibit irregular liver regeneration in response to partial hepatectomy
Source: Sci Rep. 2019 Sep 24;9:13789. doi: 10.1038/s41598-019-50219-8 (PMC6760117; doi:10.1038/s41598-019-50219-8)
Supplement: Supplementary file 1 — Supplementary Information [file 41598_2019_50219_MOESM1_ESM.pdf]

Supplementary information

Title: Mice depleted for Exchange Proteins Directly Activated by cAMP (Epac) exhibit irregular liver regeneration in response to partial hepatectomy.

List of authors: Kathrine Sivertsen Åsrud[*1], Line Pedersen[1], Reidun Aesoy[2], Haruna Muwonge[1], Elise Aasebø[2,3], Ina Katrine Nitschke Pettersen[1], Lars Herfindal[2], Ross Dobie[4], Stephen Jenkins[4], Rolf Kristian Berge[2,5], Neil Cowan Henderson[4], Frode Selheim[1,2], Stein Ove Døskeland[1] and Marit Bakke[1]

Affiliations:

[1]Department of Biomedicine, The University of Bergen, Bergen, Norway

[2]Department of Clinical Science, The University of Bergen, Bergen, Norway

[3]Department of Biomedicine, The Proteomic Unit at The University of Bergen (PROBE), University of Bergen, 5009 Bergen, Norway

[4]Centre for Inflammation Research, The University of Edinburgh, UK

[5]Department of Heart Disease, Haukeland University Hospital, Bergen, Norway

Corresponding author:
Kathrine Sivertsen Åsrud, Department of Biomedicine, University of Bergen, Jonas Lies vei 91, N-5009, Bergen, Norway
E-mail: Kathrine.Asrud@uib.no
Phone: +4755586381

Materials & Methods

Reagents

Alexa Fluor ® 488 Donkey Anti-Rat IgG (H+L) (cat.# A-21208), Alexa Fluor ® 594 Goat Anti-Rabbit IgG (H+L) (cat.# A-11037) and Prolong® Gold Antifade Reagent with DAPI (cat.# P36935) were obtained from Life Technologies (Carlsbad, CA, USA). APC anti-mouse CD31 (cat.# 102509, clone. # MEC13.3), APC anti-mouse CD31 (cat.# 102409, clone.# 390), APC/Cy7 anti-mouse/human CD11b (cat.# 101225, clone.# M1/70), FITC anti-mouse CD45.2 (cat.# 109805), PE anti-mouse CD3 (cat.# 100205, clone.# 17A2), PE anti-mouse CD19 (cat.# 115507, clone.# 6D5), PE anti-mouse Ly-6G (cat.# 127605, clone.# 1A8), PE/Cy7 anti-mouse CD45 (cat.# 103113, clone.# 30-F11), PE/Cy7 anti-mouse F4/80 (cat.# 123113, clone.# BM8) and Purified anti-mouse CD16/32 8 (cat.# 101302, clone.# 93) were from BioLegend (San Diego, CA, USA). Goat IgG Isotype Control (cat.# 02-6202, clone.# 104) and Normal Goat Serum (cat.# PCN5000) were from Invitrogen (Waltham, MA, USA). Mdr Antibody (ABCB1) (cat.# SC-8313, clone.# H-241) was from Santa Cruz Biotechnology (Dallas, Texas, USA). PE Rat Anti-Mouse Siglec-F (cat.# 562068, clone.# E50-2440) was from BD Biosciences (Franklin Lakes, NJ, USA). Rat ANTI-BrdU (cat.# MCA2060, clone.# BU1/75 (ICR1)) was from Abd Serptec (Kidlington, UK). Rat IgG2a Isotype Control (cat.# CTL-4110-100) was from Nordic BioSite AB (Täby, Sweden). GeneRuler 100 bp DNA ladder (cat.# SM0241), Goat anti-Rabbit IgG (H+L) Highly Cross-Adsorbed Secondary Antibody HRP (cat.# A16110), Shandon™ Instant Hematoxylin (cat.# 6765015), SuperSignal® West Pico Chemiluminescent Substrate, PPAR alpha Polyclonal Antibody (cat.# 42-4600), SuperSignal™ West Femto Maximum Sensitivity Substrate (cat# 34095) and Rhodamine Phalloidin (cat.# R415) was from ThermoFisher Scientific™ (Waltham, MA, USA). Aqua Pertex® (cat.# 00962) was from HistoLab (Gothenburg, Sweden). Tissue-Tek® O.C.T. Compound (cat.# 4583) was from Sakura® Finetek (The Netherlands). Chloroform licrosolv (cat.# 1.02444.2500), Methanol licrosolv (cat.# 1.06018.2500), Water licrosolv (cat.# 1.15333.1000) and Isopropanol (cat.# 1.01040.1000) were from Merck (Darmstadt, Germany). Fetal Bovine Serum (cat.# 10437010), DPBS (cat.# 14190094) and Dispase II (cat.# 17105041) were from Gibco (Carlsbad, CA, USA). SYBR Green Supermix (cat.# 170-8882) was from BioRad (Hercules, CA, USA). DNase (cat.# 11284932001) and cOMplete™ Mini Protease Inhibitor Cocktail (cat.# 11836153001) were from Roche (GmbH, Germany). Anti-Cytochrome P450 4A (cat.# ab3573), Anti-Cyclin D1 (cat.# ab134175), Anti-Cyclin E1 (cat.# ab71535), and Anti-TNFα (cat.# ab6671) was from Abcam (Cambridge, UK). Dako Liquid DAB+ Substrate Chromogen System (cat.# K3468) was from Dako (Carpinteria, CA, USA). Anti-β-actin Clone AC-15 (cat# A5441) was from Sigma Aldrich. Anti-IL 1 (cat.# 50794) was from Cell Signaling Technology (Danvers, MA, USA). If not otherwise stated, chemicals were purchased from Sigma- Aldrich.

Hematoxylin and Eosin staining

Formalin-fixed, paraffin-embedded liver sections were stained with hematoxylin and eosin (H&E) as described in [1]. Liver histology was visualized by light microscopy (Leica DMLB) and analysis Pro 3.2 software (Leica). Images, 20x-40x was taken using a Leica DC300 camera.

Oil red O staining

Oil red O staining (ORO) was carried out on cryosections (12μm). Imaging and quantifying of intracellular lipid droplets was performed as described in [2]. A minimum of 10 fields per mouse was quantified using ImageJ64 software (National Institutes of Health, Bethesda, MD), using images obtained by light microscopy (20x; Leica DMLB, DC300 camera). In

Fig.4a, the white balance in the representative images for Epac1$^{-/-}$ and Epac2$^{-/-}$ 26h post-PH has been adjusted after the image was obtained.

Hepatocyte size quantification

Hepatocyte size was quantified with TRITC-conjugated Rhodamine Phalloidin (Thermofisher) labeling of F-actin on cryosections (4µm) according to manufacturer's protocol, but with the following modification: Sections were incubated with Rhodamine Phalloidin (1:40 in PBS with 1 % BSA) for 2h at RT. The size of approximately 2000 hepatocytes per mouse was monitored using a Zeiss Axioplan2 microscope and AxioVision version 4.5 program, 20x. The Gen5 3.0 software (BioTek) was used to calculate the area per cell. Object size was set to 50-150 µm in average diameter in order to avoid any contamination by other cell types.

Lipid profiling

Lipid profiling was performed on plasma (250 µl) and liver tissue (150 mg) from control animals or from mice subjected to PH using enzymatic calorimetry based on the Trinder reaction on a Hitachi 917 automated instrument (Roche) using commercially available kits: Cholesterol, HDL-Cholesterol, LDL-Cholesterol, triglycerides and glucose (Roche), NEFA FS, free cholesterol FS and phospholipids FS (Diasys). Liver lipids were extracted according to Bligh and Dyer [3].

Immunohistochemistry (IHC)

Paraffin sections were deparaffinized in xylene, rehydrated using graded alcohol series (xylene for 2x5 min, 100 % EtOH for 2x3 min, 96 % EtOH for 2x3 min), and washed in distilled water. Antigen retrieval was performed in Tris-EDTA buffer (10 mM Tris Base, 1 mM EDTA solution, 0.05 % Tween, pH= 9.0) at 98° C for 20 min followed by cooling in running tap water for 10 min. The sections were then incubated in TBS-T for 30 min at RT followed by blocking buffer [10% normal goat serum in 1 % BSA/TBS) for 2h at RT. Next, the sections were incubated with primary antibody Anti-ABCB1 (1:50 in 1 % BSA/TBS) o/n at 4° C on an orbital shaker. Parallel sections were used as negative controls and incubated in goat IgG Isotype Control or in 1 % BSA/TBS. Subsequently, sections were rinsed for 3x5 min in TBS-T, and incubated in Alexa Fluor® 594 Goat Anti-Rabbit IgG, (1:200 in 1 % BSA /TBS) for 1h at RT. Finally, sections were rinsed in running tap water for 5 min, counterstained, and mounted with Prolong(R) Gold Antifade Reagent with DAPI and coverslipped (Menzel Gläzer). ABCB1 distribution was visualized using a Zeiss Axioplan2 microscope and AxioVision version 4.5 program (Zeiss). For each mouse, the canalicular length per cell was measured in three 20x microscopic fields (141.9 mm^2) in three different sections. Images were analyzed using ImageJ64 (National Institutes of Health, Bethesda, MD). For each image, a grid function with a horizontal grid type with an area per point of 0.5 inches2 (equals 10 lines per image) was applied. The number of canaliculi (ABCB1) and the number of DAPI stained nucleus crossing each line was counted manually. For each mouse, the canalicular length per cell was calculated by dividing the total number of canaliculi by the total number of DAPI stained nucleus. IHC staining of PDGFβ-positive stellate cells and F4/80-positive Küpffer cells was performed as described in[4].

CYP4A immunolocalization

For CYP4A immunolocalization on paraffin-embedded sections, the same IHC procedure was performed with the following alternations: The antigen retrieval step was performed in sodium citrate buffer (10 mM sodium citrate, pH 6.0), and the sections incubated with

primary antibody (anti-CYP4A, 1:100 in 1% BSA/PBS) o/n. Following 3x5 min wash in PBS, the sections were blocked in 0.3 % H_2O_2/PBS in 15 min, RT. Next, sections were rinsed for 3x5 min in PBS and incubated with secondary antibody (Goat anti-rabbit, HRP 1:500 1% BSA/PBS) in 2h at RT, followed by colorimetric detection with a Dako Liquid DAB+ Substrate Chromogen System for 15 min. Finally, the sections were rinsed in PBS (5 min) and ddH_2O (5 min) before counterstained with hematoxylin (1 min), rinsed 3x5 min in ddH_2O and mounted with Aqua Pertex.

Immunoblotting

To determine the expression levels of CYP4A (10, 12 and 14), Cyclin D1, Cyclin E1, IL-1, TNFα and PPARα in the liver, 40 µg of whole liver extracts in RIPA buffer containing cOMplete™ Mini Protease Inhibitor Cocktail was separated by 12 % (w/vol) SDS/PAGE and transferred to Hybond™ P 0.45µm PVDF membrane (GE-Healthcare) using either anti-CYP4A (1:1000 in PBS-T o/n), anti-Cyclin D1 (1:10 000 in TBS-T o/n), anti- Cyclin E1 (1: 2000 in TBS-T o/n), Anti-IL1 (1:1000 in 5% BSA), Anti-TNFα (1:500 in 1% BSA/TBS-T(0.1% o/n) or anti-PPARα (1:125 in PBS-T o/n) as primary antibodies, and HRP-conjugated anti-Rabbit IgG (H+L) (1:10 000 in PBS-T, 2h at RT) as secondary antibody. Blots were developed with ECL using SuperSignal® West Pico Chemiluminescent Substrate and evaluated with the Luminescent Image Analyzer LAS-3000 (Fujifilm). Relative intensity of the bands was quantified and normalized to a loading control (β-actin, 1:10 000 o/n) using ImageJ64 as described in http://lukemiller.org/index.php/2010/11/analyzing-gels-and-western-blots-with-image-j/.

Proteomic analyses

Sample preparation for proteomics

Frozen liver tissue (10-12 µg) harvested pre- and 36h post-PH was lysed in sodium dodecyl sulfate (SDS) lysis buffer (4% SDS in 0.1 M Tris-HCl, pH 7.6) and processed for liquid chromatography mass spectrometry (LC-MS) analysis according to the filter-aided sample preparation (FASP) procedure [5], as described step by step elsewhere [6]. The peptides were desalted as described in [6], and lyophilized in a vacuum concentrator (Centrivap with a Cold trap, Labconco, MO) at 30 ºC.

NanoLC-MS

The peptides were resolved in 1 % aqueous formic acid (FA) / 2% acetonitrile (ACN), and 1 µg of each sample was injected into an Ultimate 3000 RSLC system (Thermo Scientific, Sunnyvale, California, USA) coupled online with positive electrospray ionization on a Q-Exactive HF mass spectrometer (Thermo Scientific, Bremen, Germany). Trapping and desalting of peptides were performed on a pre-column (Acclaim PepMap 100, 2cm x 75µm i.d. nanoViper column, packed with 3µm C18 beads), and separated on a 50 cm analytical column (Acclaim PepMap100 nanoViper column, 75 µm i.d. × 50 cm, packed with 3 µm C18 beads) at a flow rate of 200 nl/min. The peptides were eluted with a biphasic ACN gradient with 0.1% FA (solvent A) and 100% ACN (solvent B) for 240 min (5% B during trapping for 5 min followed by 5-8% B over 0.5 min, 8-24% B over 149.5 min, 24–35% B over 30 min, then to 90% B over 10 min, hold at 90% B for 20 min, then sloped to 5% B over 5 min and hold at 5 % B for 20 min).

DDA, Q-Exactive HF

The Q-Exactive HF was set automatically to switch between full scan MS and MS/MS acquisition in data-dependent-acquisition-mode (DDA). Full MS spectra were acquired in the scan range 375-1500 m/z with resolution R=120,000 and automatic gain control (AGC)

target of 3e6 with a maximum injection time (IT) of 100 ms. The 15 most intense peptides with an intensity threshold of 5e4, charge states 2 or more, were isolated to a target AGC value of 1e5, resolution R=30,000, IT of 45 ms and 28 % normalized collision energy. The isolation window was 1.6 m/z with an isolation offset of 0.3 m/z and a dynamic exclusion of 30 seconds.

Label-free protein quantification and data analysis

The raw MS files were searched against the reviewed SwissProt *Mus musculus* database (downloaded 06.01.2016, containing 24 797 canonical and isoform sequences) using the MaxQuant module (version 1.5.1.2) with the integrated Andromeda search engine [7]. MaxQuant´s settings for identification and label-free protein quantification were as described previously[8]. Briefly, carbamidomethylation of cysteine was set as fixed modification and acetylation of protein N-terminal and oxidation of methionine were set as variable modifications. The label-free quantification (LFQ) algorithm [9] was used for quantification with LFQ min. ratio count set to 1, and the match-between-runs option was enabled.

The protein output from the MaxQuant analysis was processed, filtered and log_2-transformed using the Perseus (version 1.5.1.6) platform [10]. Reverse hits and proteins only identified by site were removed from the dataset. The samples were grouped into the following four groups 1) WTprePH 2) WTpostPH 3) Epac1/2$^{-/-}$prePH 4) Epac1/2$^{-/-}$postPH, and at least three valid values were required for statistical evaluation of significant differences between two groups.

Statistical analysis

The statistical significance was calculated in excel, using a two-tailed paired *t*-test for statistical evaluation of the same mice (wt or Epac1/2$^{-/-}$) before and after regeneration. A two-tailed student's two-sample *t*-test (assuming unequal variance) was applied for statistical analysis between wt and Epac1/2$^{-/-}$ mice (before or after regeneration). A p-value < 0.05 was considered as significantly different. The fold changes (FC) between the different groups were found by subtracting the log_2 median of one group from the other. To capture the most biologically interesting differences, we applied Z-statistics to evaluate the significance of the FC-values according to [11]. Here, significance is based on the upper and lower standard deviations of the normal distribution of the FC's, and a p-value < 0.05 was considered as a *significant FC*.

Gene ontology (GO) and UniProt-keyword enrichment analyses of the significantly differentially expressed proteins (p-value < 0.05 with both *t*-test and Z-statistics) returned from the four comparisons were performed with the online resource A.GO.TOOL [12]. The default parameters were used, except that the abundance correction was unselected and the GO-terms were enriched individually. The "filter hierarchy" setting was applied on the results to reduce the number of similar GO-terms. The probability that a set of proteins corresponds to a certain biological function was calculated using Fisher exact test, followed by Benjamini Hochberg multiple hypothesis testing of the uncorrected p-values. The FDR corrected p-values were used for further interpretation.

Supplementary Figures

Fig.S1

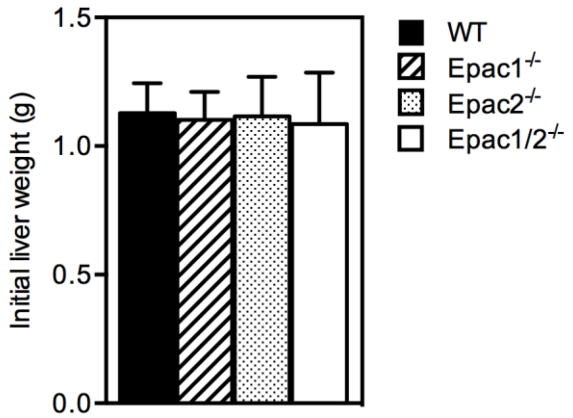

Fig. S1. No differences in initial liver weight between genotypes. Wt, Epac1$^{-/-}$, Epac2$^{-/-}$ and Epac1/2$^{-/-}$ male mice (8-12 weeks old) were subjected to 2/3 PH. The initial total liver weight was calculated as follows: (resected liver weight (g)/ 68 (%) x 100 (%)), n= 26-37 mice/group. Data is presented as mean ± SD. One-way ANOVA with Bonferroni`s adjustment for multiple comparisons was used to determine potential significant differences between genotypes, not significant (ns); F-statistics: $F(3, 122)= 0.5402$, $p=0.6557$.

Supplementary Figures

Fig. S2

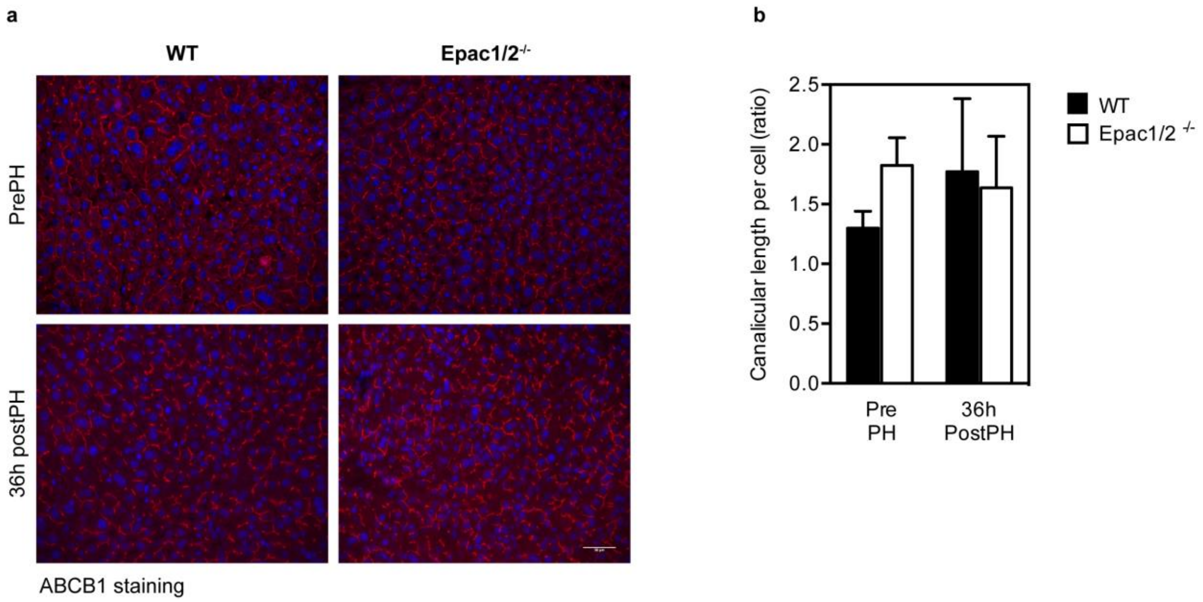

Fig. S2. No alteration in ABCB1 distribution in Epac1/2$^{-/-}$ mice. a) Shown are representative images of IHC-staining of ABCB1 (red) and DAPI (blue), in whole liver tissue harvested from wt mice and Epac1/2$^{-/-}$ mice pre- and 36h post-PH, 20x. **b)** Quantification of canalicular length per cell in wt (black bars) and Epac1/2$^{-/-}$ (white bars) mice pre- and 36h post-PH. For each mouse, the canalicular length per cell was measured in three 20x microscopic fields (141.9 mm^2) in three different sections. Please see the IHC-section in supplementary M&M for further details. Values represent mean ± SD, n=3-4 mice/group. Two-way ANOVA was used to determine differences between genotypes, no significant differences, F-statistics: $F(1, 10)= 2.248$, $p=0.1647$.

Supplementary Figures

Fig.S3

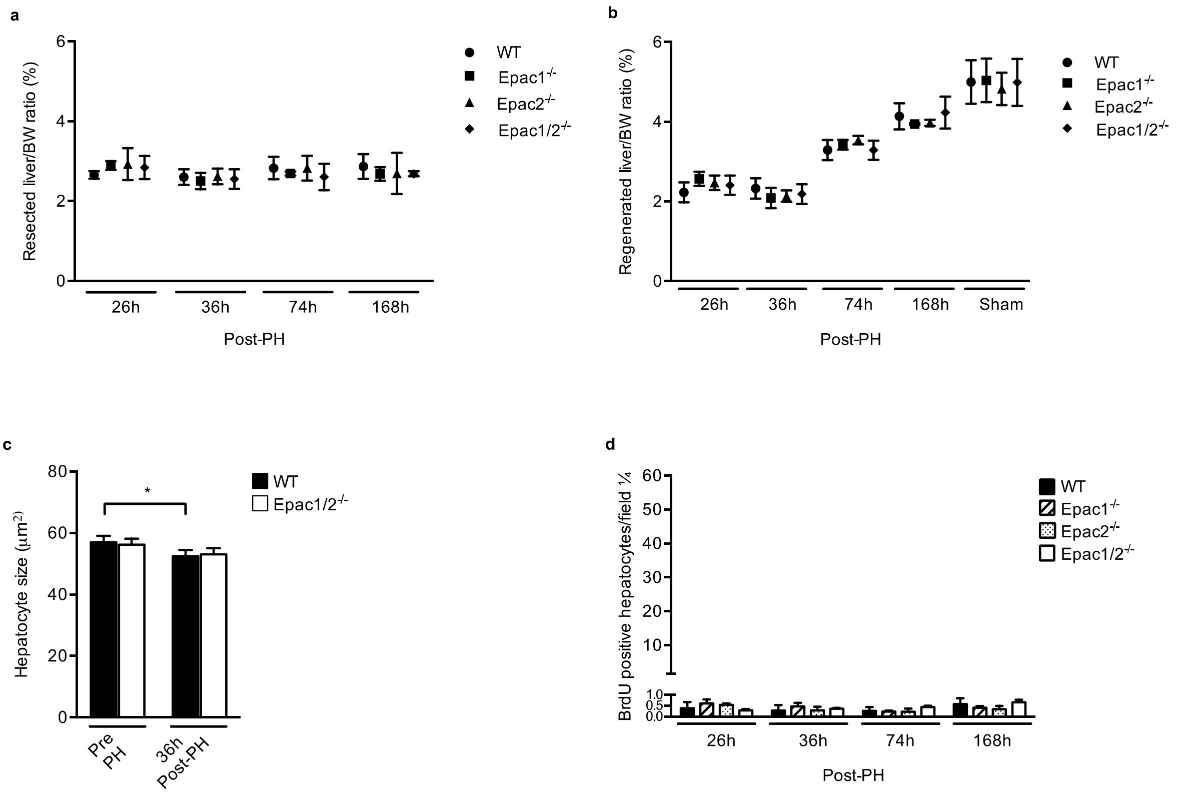

Fig. S3. Mice depleted for Epac1/2 display no adverse phenotypes in response to PH compared to wt mice. Wt, Epac1$^{-/-}$, Epac2$^{-/-}$ and Epac1/2$^{-/-}$ male mice (8-12 weeks old) were subjected to 2/3 PH or laparotomy (sham), injected with BrdU 2h prior sacrifice and culled at four selected post- operative time points; 26h, 36h, 74h and 168h. Data is presented as mean ± SD. Two-way ANOVA with Bonferroni`s adjustment for multiple comparisons when applicable was used to determine potential significant differences between genotypes in each treatment group. **a)** Equal resected liver/BW ratio (%) between genotypes at all time points**.** 26h/36h (n=6-13), 74h (n=4-10) and for 168h (n=2-6), not significant (ns); F-statistics: F(9,106)= 1.188, p=0.3104. **b)** Normal restitution of hepatic index in mice deleted for Epac1/2 after 2/3 PH. There was no difference in mean regenerated liver/BW ratios between genotypes at any time points. 26h/36h (n=6-13), 74h (n=4-10), 168h (n=2-6) and for sham operated animals (n=11-12), not significant (ns); F-statistics: F(12,147)= 1.054, p=0.4037. **c)** Quantification of hepatocyte size in wt and Epac1/2$^{-/-}$ mice pre- and 36h post-PH. For each mouse, three sections were prepared and approximately 2000 F-actin labeled hepatocytes were counted, 20x. n=2-3 mice/group, F-statistics: F(1,5)= 0.6236, p=0.4655. **d)** BrdU incorporation in sham-operated mice**.** n= 3 mice/group, not significant (ns); F-statistics: F(9,32)= 1.968, p=0.0770.

Supplementary Figures

Fig.S4

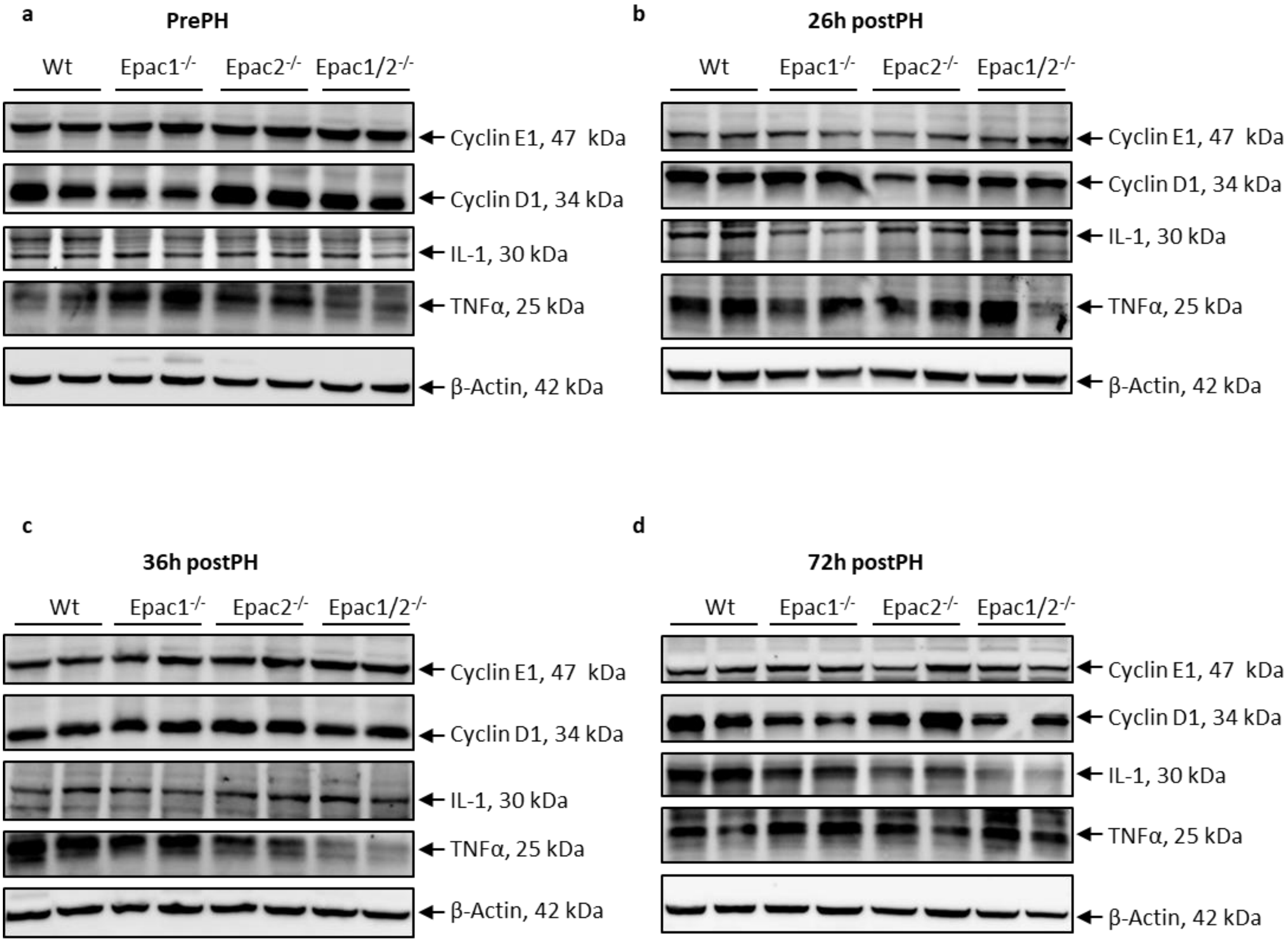

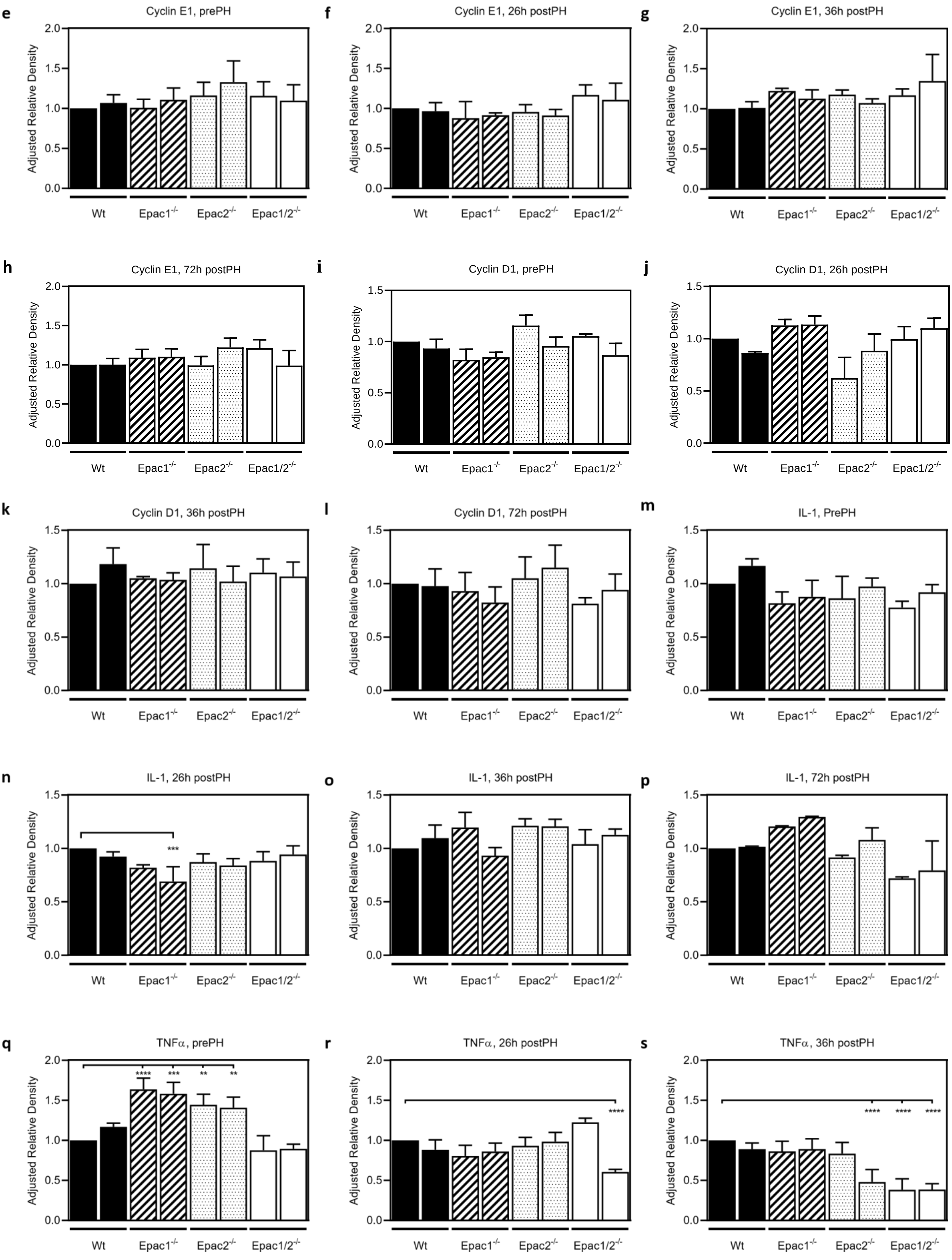
e
Cyclin E1, prePH
f
Cyclin E1, 26h postPH
g
Cyclin E1, 36h postPH
h
Cyclin E1, 72h postPH
i
Cyclin D1, prePH
j
Cyclin D1, 26h postPH
k
Cyclin D1, 36h postPH
l
Cyclin D1, 72h postPH
m
IL-1, PrePH
n
IL-1, 26h postPH
***
o
IL-1, 36h postPH
p
IL-1, 72h postPH
q
TNFα, prePH
**** *** ** **
r
TNFα, 26h postPH
****
s
TNFα, 36h postPH
**** **** ****
Adjusted Relative Density
Wt
Epac1-/-
Epac2-/-
Epac1/2-/-

t

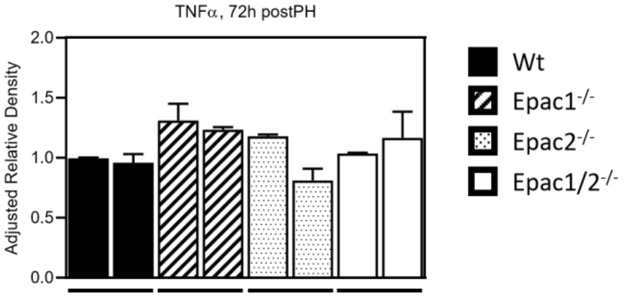

Fig. S4. Immunoblotting of cell cycle markers.
The expression levels of Cyclin E1, Cyclin D1, IL-1 and TNFα in wt, Epac1$^{-/-}$, Epac2$^{-/-}$ and Epac1/2$^{-/-}$ mice pre- and post-PH was determined by western blotting (WB). The data is shown as mean(SD), with n=2 mice of each genotype per time point. **a-d)** Representative WB of Cyclin D1, Cyclin E1, IL-1 and TNFα expression in wt, Epac1$^{-/-}$, Epac2$^{-/-}$ and Epac1/2$^{-/-}$ mice **a)** pre-, **b)** 26h-, **c)** 36h- and **d)** 72h post-PH. **e-t)** Quantification: The WB in a-d) was repeated 3-5 times, and the densiometrically determined (Cyclin E1/Cyclin D1/Il-1/TNFα)/β-actin ratios were normalized to the value of one wt individual (black bar to the very left) in each blot. The Cyclin E1, Cyclin D1 and TNFα and β-actin blots displayed in a) are cropped from the same Hybond^{TM} P 0.45μm PVDF membrane, whereas IL-1 is cropped from another membrane. The Cyclin E1, Cyclin D1, TNFα and β-actin blots displayed in b) and c) respectively, are also cropped from the same membrane, whereas the IL-1 blots in b-d) and TNFα blot in d) are cropped from different membranes (see Supplementary Figure S5 for the full- length blots).

e-h) Quantification of Cyclin E1.
e) Quantification of Cyclin E1 PrePH; Quantification of Cyclin D1 26h postPH; 4 WB, One-way ANOVA with Dunnett`s multiple comparison test, F(7,24)=1.629, p=0.1725, Brown-Forsythe test, p=0.2649. **f)** Quantification of Cyclin E1 26h postPH; of 5 WB, One-way ANOVA with Dunnett`s multiple comparison test, F(7,32)=3.239, *p=0.0103, Brown-Forsythe test, p=0.1335. **g)** Quantification of Cyclin E1 36h postPH; 3 WB, One-way ANOVA with Dunnett`s multiple comparison test, F(7,16)=2.261, p=0.0838, Brown-Forsythe test, p=0.3373.**h)** Quantification of Cyclin E1 72h postPH; 4 WB, Kruskal-Wallis with Dunn`s multiple comparison test; Kruskal-Wallis statistic: 14.45 Approximate *p=0.0427.

i-l) Quantification of Cyclin D1.
i) Quantification of Cyclin D1 PrePH; 3 WB, One-way ANOVA with Dunnett`s multiple comparison test, F(7,16)=5.822, $^{**}$p=0.0017, Brown-Forsythe test, p=0.8177. **j)** Quantification of Cyclin D1 26h postPH; 4 WB, Kruskal-Wallis with Dunn`s multiple comparison test; Kruskal-Wallis statistic: 20.68, Approximate **p=0.0043. **k)** Quantification of Cyclin D1 36h postPH; 3 WB, One-way ANOVA with Dunnett`s multiple comparison test, F(7,16)=0.7209, p=0.6566, Brown-Forsythe test, p=0.6755. **l)** Quantification of Cyclin D1 72h postPH; 3 WB, One-way ANOVA with Dunnett`s multiple comparison test, F(7,16)=1.596, p=0.2071, Brown-Forsythe test, p=0.8396.

m-p) Quantification of IL-1.
m) Quantification of IL-1 PrePH; 3 WB One-way ANOVA with Dunnett`s multiple comparison test, F(7,16)=3,729, $^{*}$p=0.0139, Brown-Forsythe test, p=0.7187.**n)** Quantification of IL-1 26h postPH; 3 WB, One-way ANOVA with Dunnett`s multiple comparison test,

F(7,16)=0.4.550, **p=0.0058, Brown-Forsythe test, p=0.8411. **o)** Quantification of IL-1 36h postPH; 3 WB, One-way ANOVA with Dunnett`s multiple comparison test, F(7,16)=3.679, *p=0.0147, Brown-Forsythe test, p=0.7765. **p)** Quantification of IL-1 72h postPH; 2 WB, Kruskal-Wallis with Dunn`s multiple comparison test; Kruskal-Wallis statistic: 13.98, Approximate p=0.0516

q-t) Quantification of TNFα.
q) Quantification of TNFα PrePH; 3 WB, One-way ANOVA with Dunnett`s multiple comparison test, F(7,16)=20.11, p<0.0001, Brown-Forsythe test, p=0.7297**. r)** Quantification of TNFα 26h postPH; 3 WB, One-way ANOVA with Dunnett`s multiple comparison test, F(7,16)=10.26, ****p<0.0001, Brown-Forsythe test, p=0.8205.**s)** Quantification of TNFα 36h postPH; 3 WB, One-way ANOVA with Dunnett`s multiple comparison test, F(7,16)=16.49, ****p<0.0001, Brown-Forsythe test, p=0.8717. **t)** Quantification of TNFα 72h postPH; 2 WB, Kruskal-Wallis with Dunn`s multiple comparison test; Kruskal-Wallis statistic: 12.67, Approximate p=0.0806.

Supplementary Figures

Fig. S5 Full length blots to Supplementary Fig.S4

Full length blots to Supplementary Fig.S4, PrePH

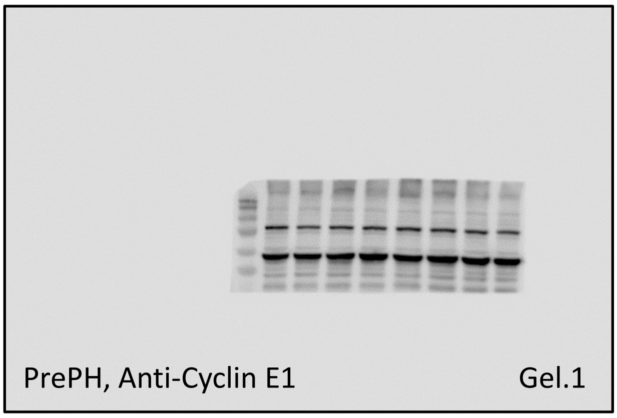
PrePH, Anti-Cyclin E1 Gel.1

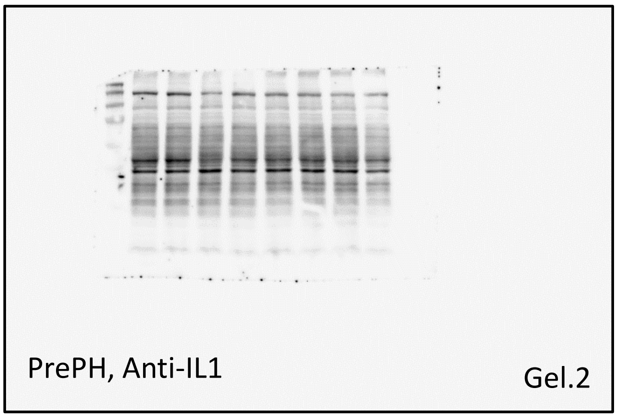
PrePH, Anti-IL1 Gel.2

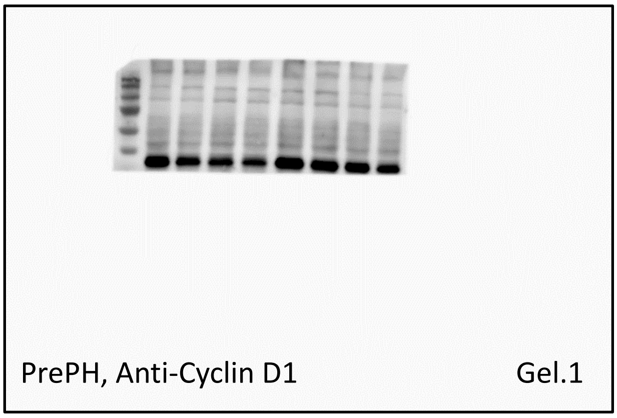
PrePH, Anti-Cyclin D1 Gel.1

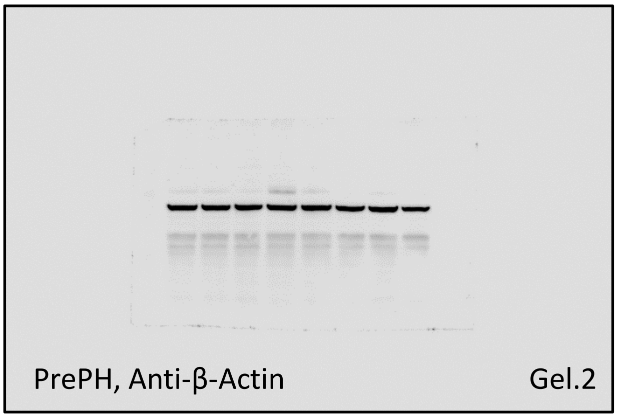
PrePH, Anti-β-Actin Gel.2

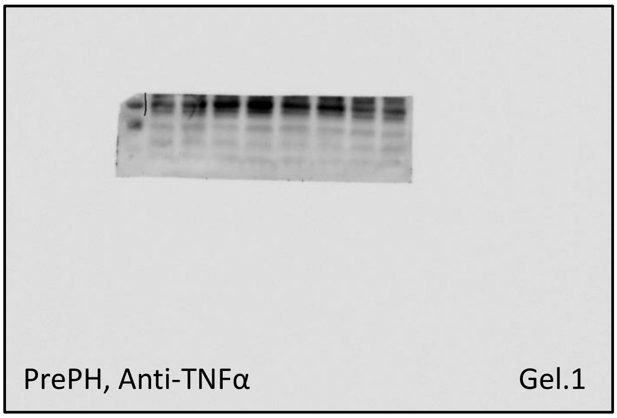
PrePH, Anti-TNFα Gel.1

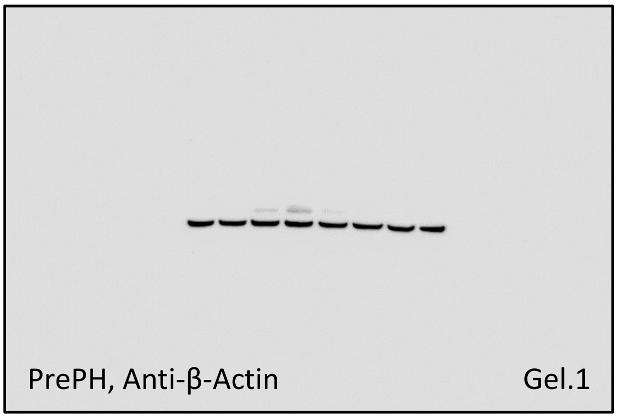
PrePH, Anti-β-Actin Gel.1

Full length blots to Supplementary Fig.S4, 26h postPH

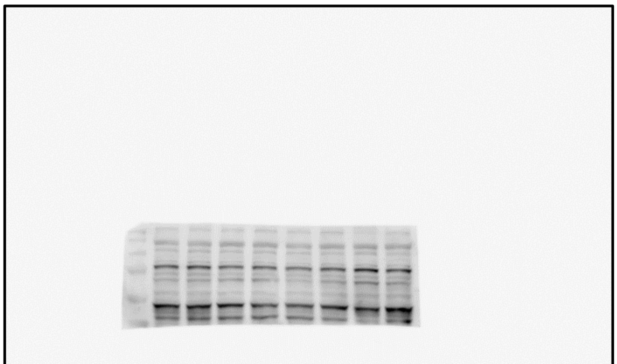

26h postPH, Anti-Cyclin E1 Gel.1

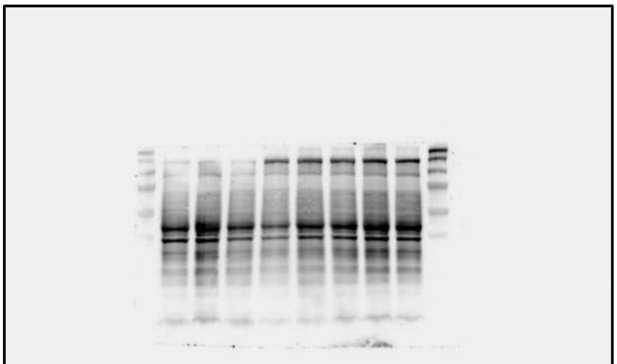

26h postPH, Anti-IL1 Gel.2

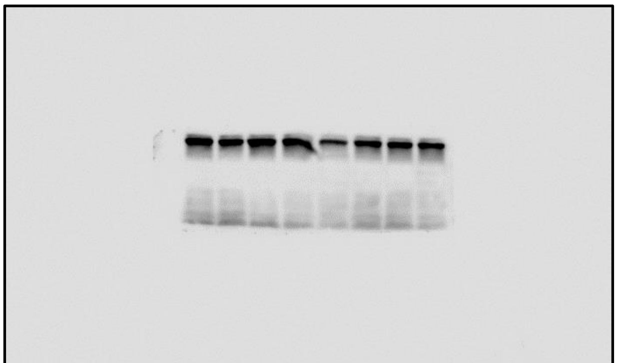

26h postPH, Anti-Cyclin D1 Gel.1

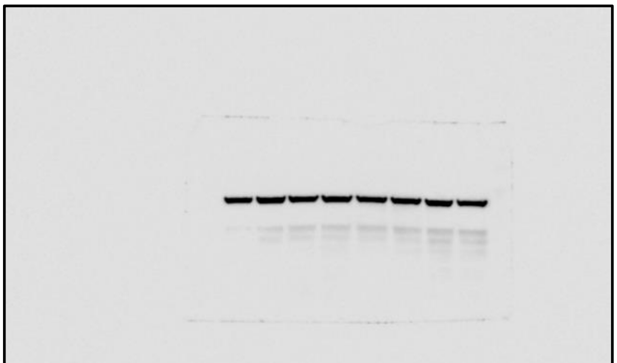

26h postPH, Anti-β-Actin Gel.2

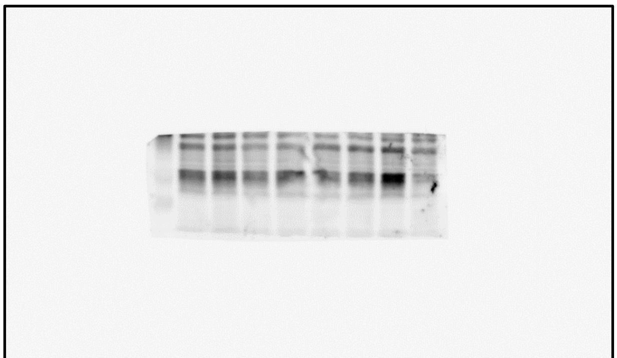

26h postPH, Anti-TNFα Gel.1

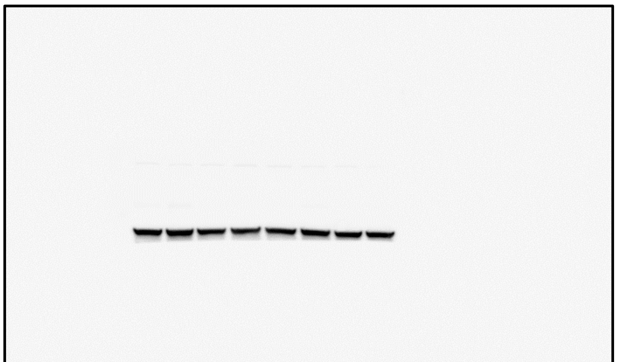

26h postPH, Anti-β-Actin Gel.1

Full length blots to Supplementary Fig.S4, 36h postPH

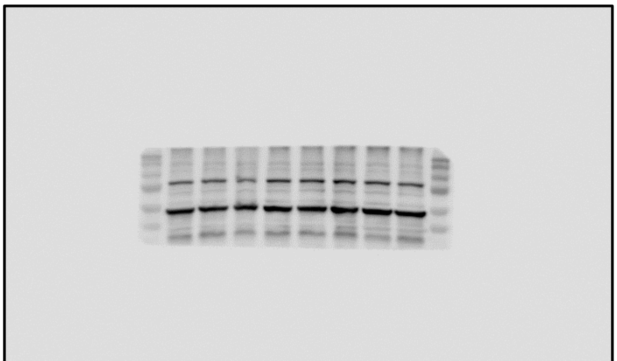

36h postPH, Anti-Cyclin E1 Gel.1

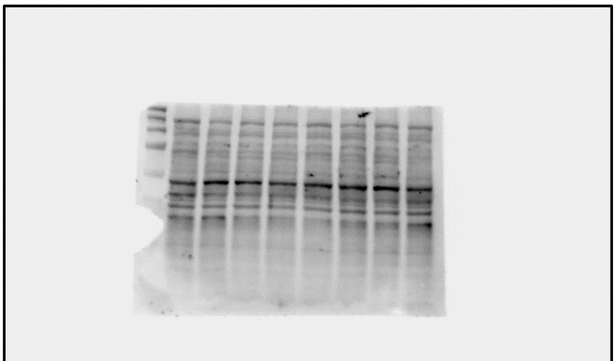

36h postPH, Anti-IL1 Gel.2

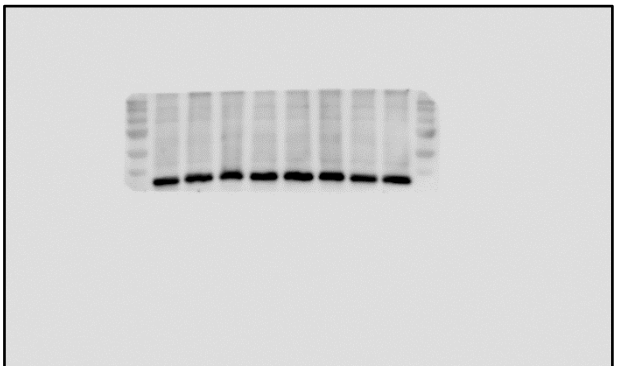

36h postPH, Anti-Cyclin D1 Gel.1

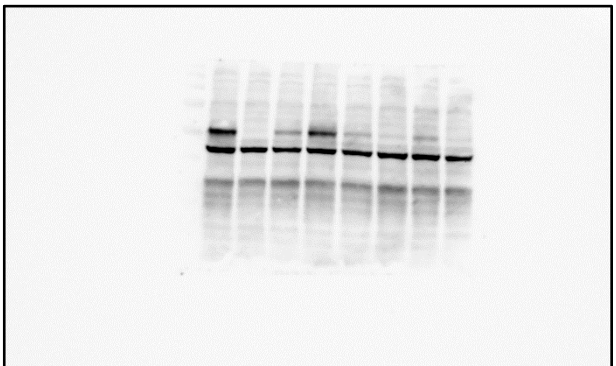

36h postPH, Anti-β-Actin Gel.2

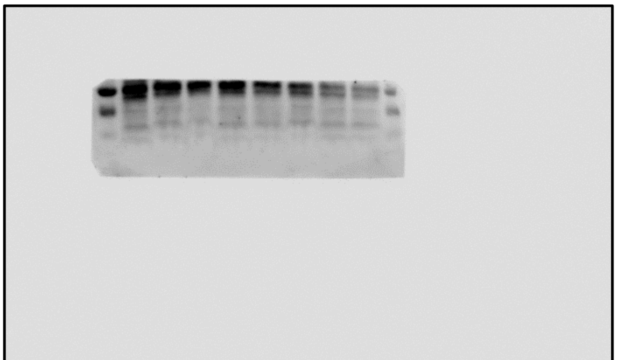

36h postPH, Anti-TNFα Gel.1

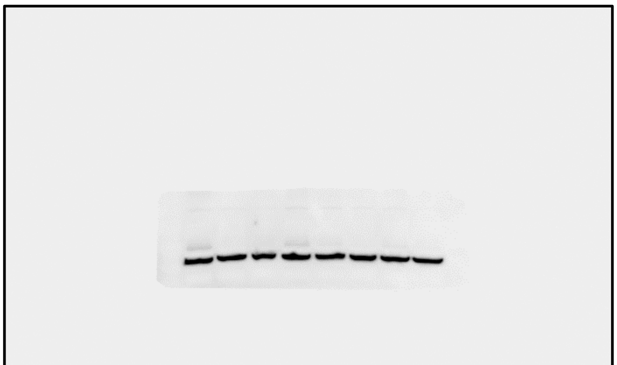

36h postPH, Anti-β-Actin Gel.1

Full length blots to Supplementary Fig.S4, 72h postPH

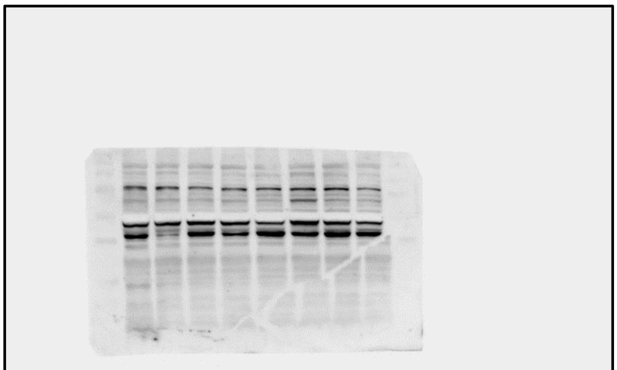

72h postPH, Anti-Cyclin E1 Gel.1

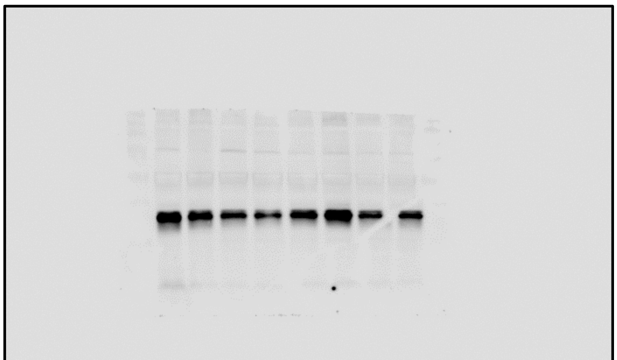

72h postPH, Anti-Cyclin D1 Gel.1

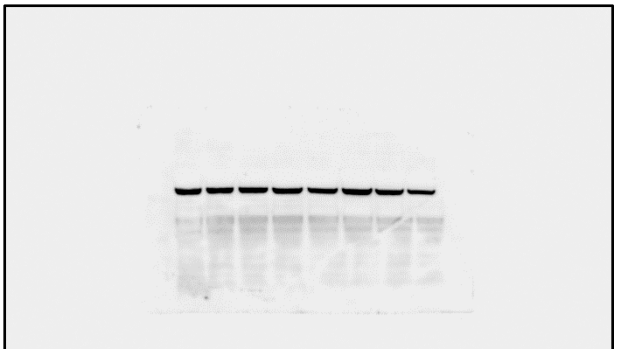

72h postPH, Anti-β-Actin Gel.1

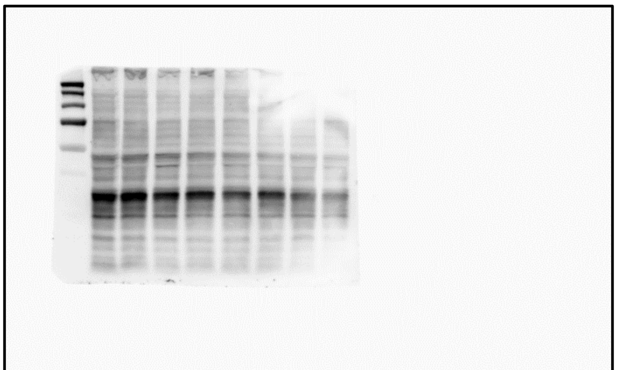

72h postPH, Anti-IL1 Gel.2

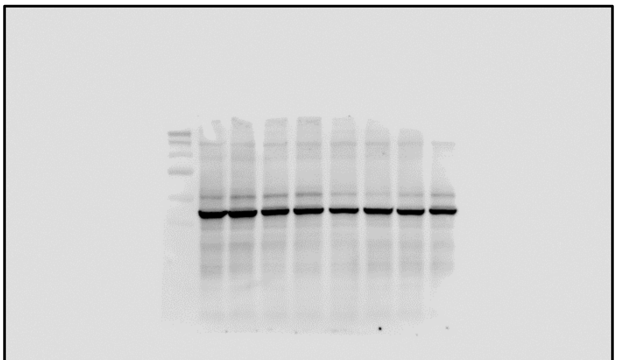

72h postPH, Anti-β-Actin Gel.2

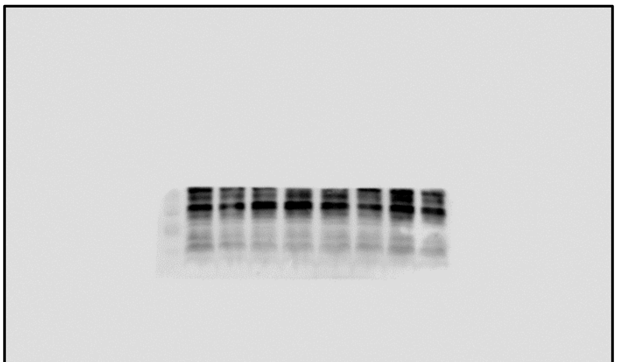

72h postPH, Anti-TNFα Gel.3

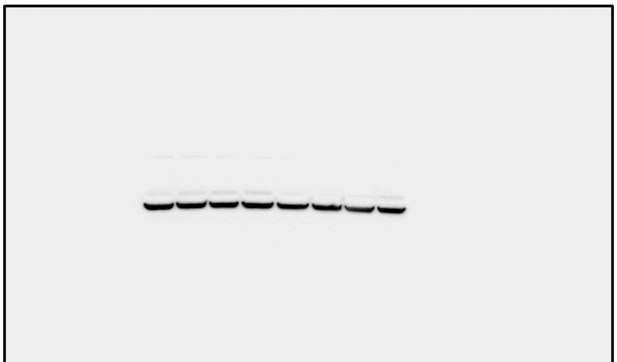

72h postPH, Anti-β-Actin Gel.3

Supplementary Figures

Fig.S6a

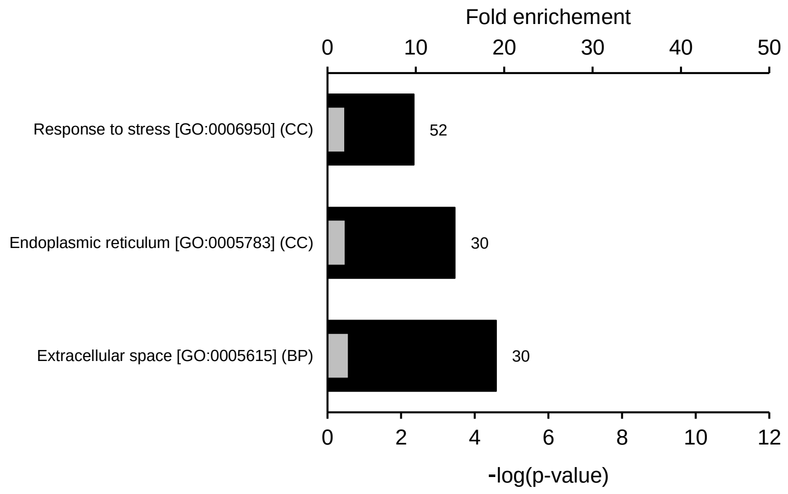

Supplementary Figures

Fig.S6b

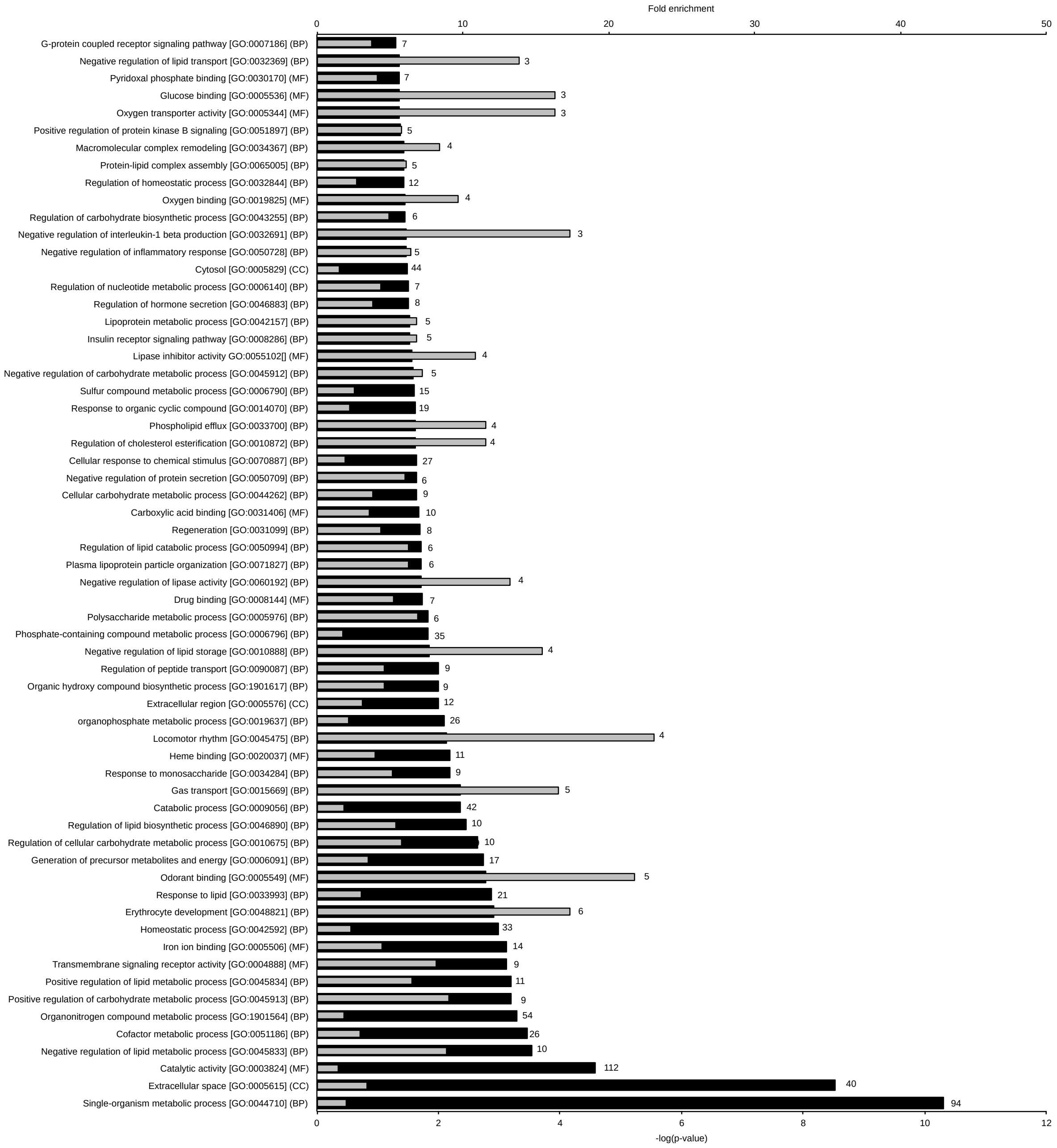

Supplementary Figures

Fig.S6c

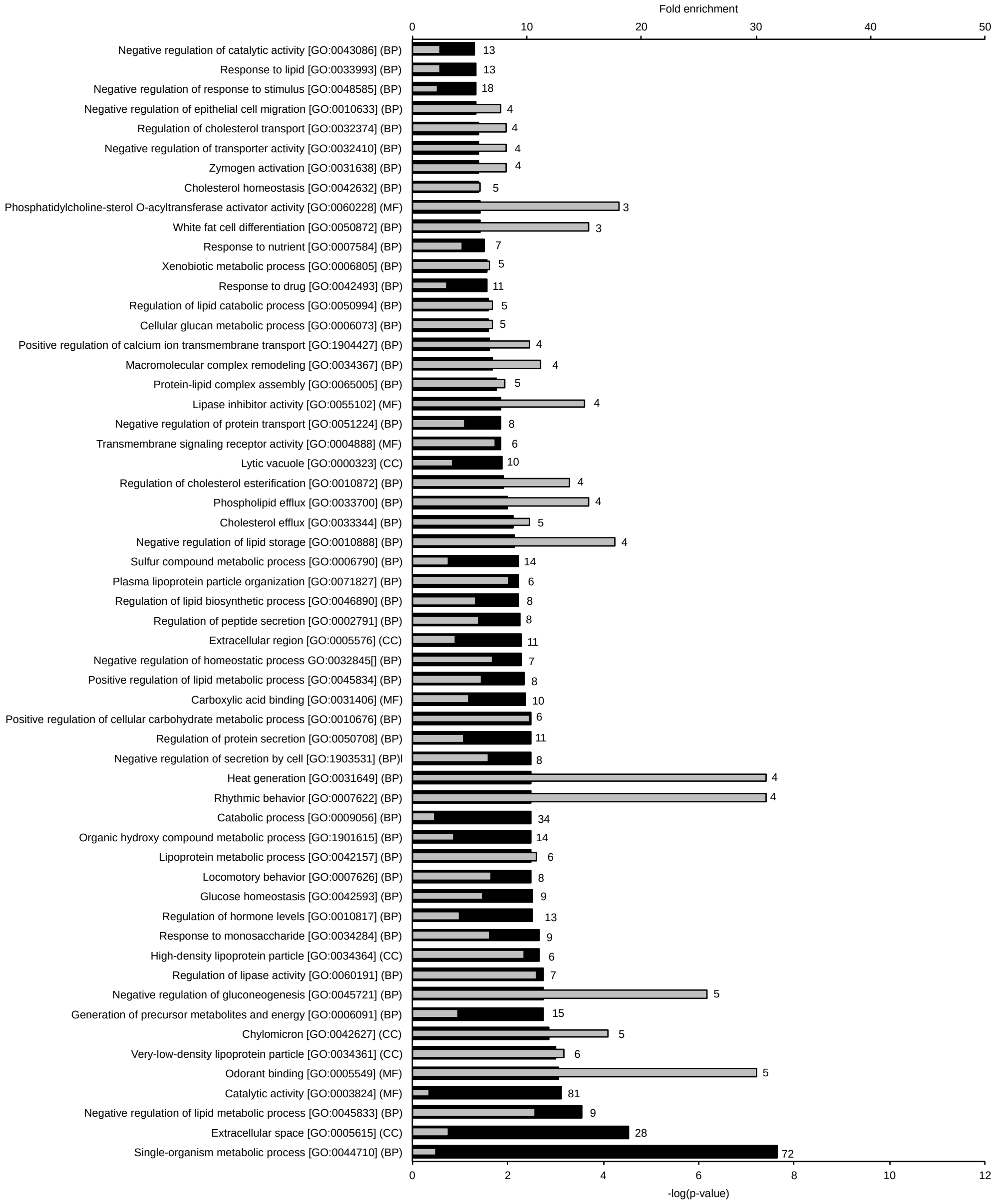

Fig. S6. GO-enrichment analyses of proteins that were differentially expressed pre- and 36h post-PH in wt and in Epac1/2$^{-/-}$ mice. a) GO analyses based on proteins that are significantly increased post-PH compared to pre-PH in wt mice. **b)** GO analyses based on proteins that are significantly decreased post-PH compared to pre-PH in wt mice. **c)** GO analyses based on proteins that are significantly decreased post-PH compared to pre-PH in Epac1/2$^{-/-}$ mice. In wt mice, a total of 3670 proteins were quantified in resected (wt-prePH) and regenerated (wt-postPH) tissue. Of these proteins, 331 had significantly changed abundance post-PH. In Epac1/2$^{-/-}$ mice, out of a total of 3694 proteins identified, 267 proteins were identified as differentially expressed when comparing Epac1/2$^{-/-}$ mice pre- and post-PH. Black bars indicate the respective FDR corrected p-values of the GO-terms. Grey bars indicate the fold enrichment. The number of proteins annotated with the corresponding function is shown behind each bar. BP: Biological processes; MF: Molecular function; CC: Cellular compartment.

Supplementary Figures

Fig. S7

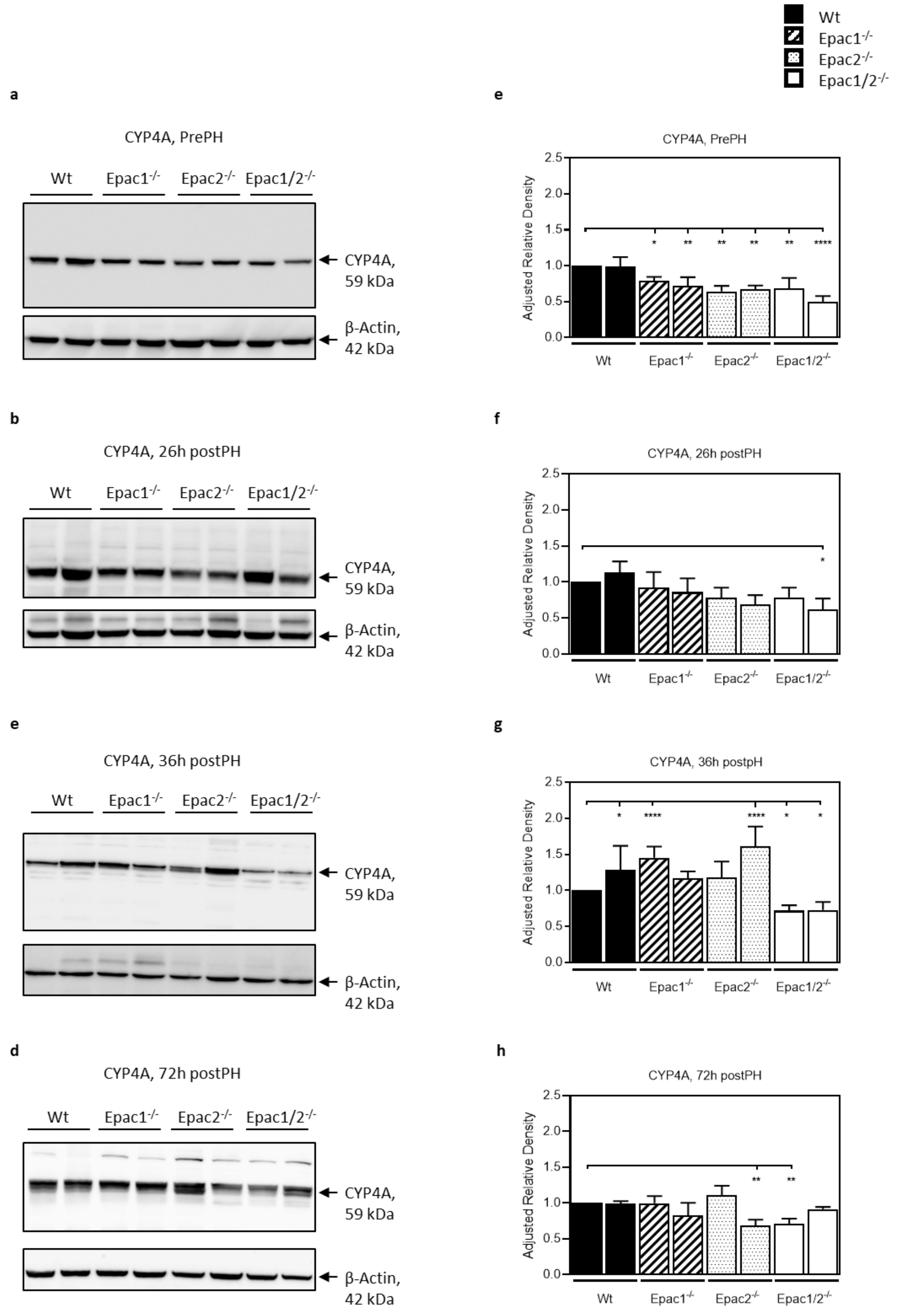

Fig. S7. Decreased expression of CYP4A in mice deleted for Epac1/2.
The expression levels of CYP4A in wt, Epac1$^{-/-}$, Epac2$^{-/-}$ and Epac1/2$^{-/-}$ mice pre- and post-PH was determined by western blotting (WB). The data is shown as mean(SD), with n=2 mice of each genotype per time point. **a-d)** Representative WB of CYP4A expression in wt, Epac1$^{-/-}$, Epac2$^{-/-}$ and Epac1/2$^{-/-}$ mice **a)** pre-, **b)** 26h-, **c)** 36h- and **d)** 72h post-PH. **e-h)** Quantification: The WB in a-d) was repeated 3-4 times, and the densiometrically determined CYP4A/β-actin ratios were normalized to the value of one wt individual (black bar to the very left) in each blot. **e)** Quantification of **a** PrePH; 3 WB, One-way ANOVA with Dunnett`s multiple comparison test, $F(7,16)=9.74$, $^{****}p<0.0001$, Brown-Forsythe test, $p=0.5993$. **f)** Quantification of **b** 26h postPH; 3 WB, One-way ANOVA with Dunnett`s multiple comparison test, $F(7,16)=3.667$, $^{*}p=0.0149$, Brown-Forsythe test, $p=0.8555$.**g)** Quantification of **c** 36h postPH; 4 WB, One-way ANOVA with Dunnett`s multiple comparison test, $F(7,32)=18.77$, $^{****}p<0.0001$, Brown-Forsythe test, $p=0.1108$. **h)** Quantification of **d** 72h postPH; 3 WB, One-way ANOVA with Dunnett`s multiple comparison test, $F(7,16)=7.563$, $^{***}p=0.0004$, Brown-Forsythe test, $p=0.4598$. The CYP4A and β-actin blots displayed in **a)** are cropped from different parts of the same Hybond^{TM} P 0.45μm PVDF membrane, the same setup applies for CYP4A and β-actin bands displayed in b-d) (see Supplementary Figure S8 the full- length blots).

Supplementary Figures

Fig. S8 Full length blots to Supplementary Fig.S7, CYP4A and β-Actin

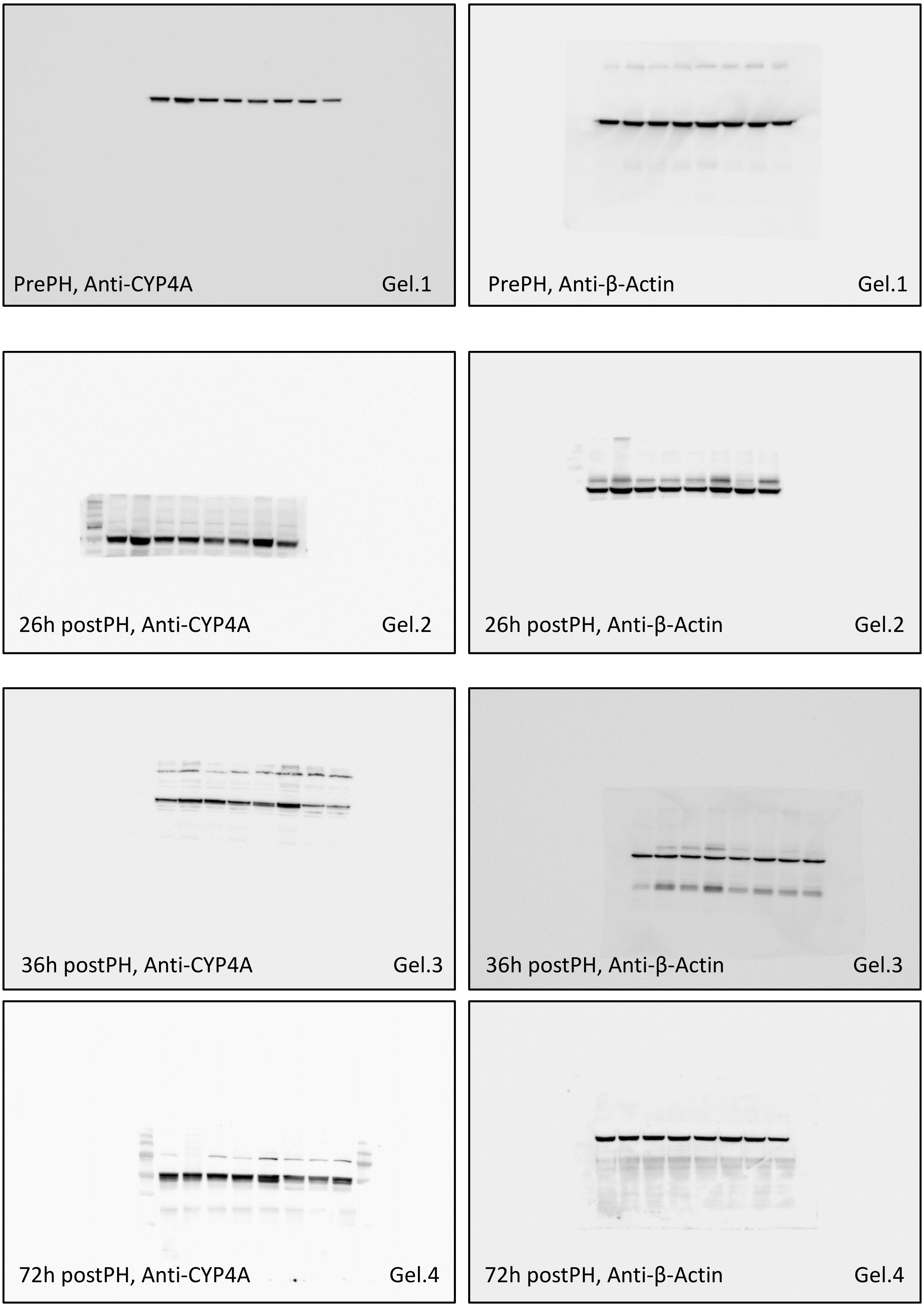

Supplementary Figures

Fig. S9

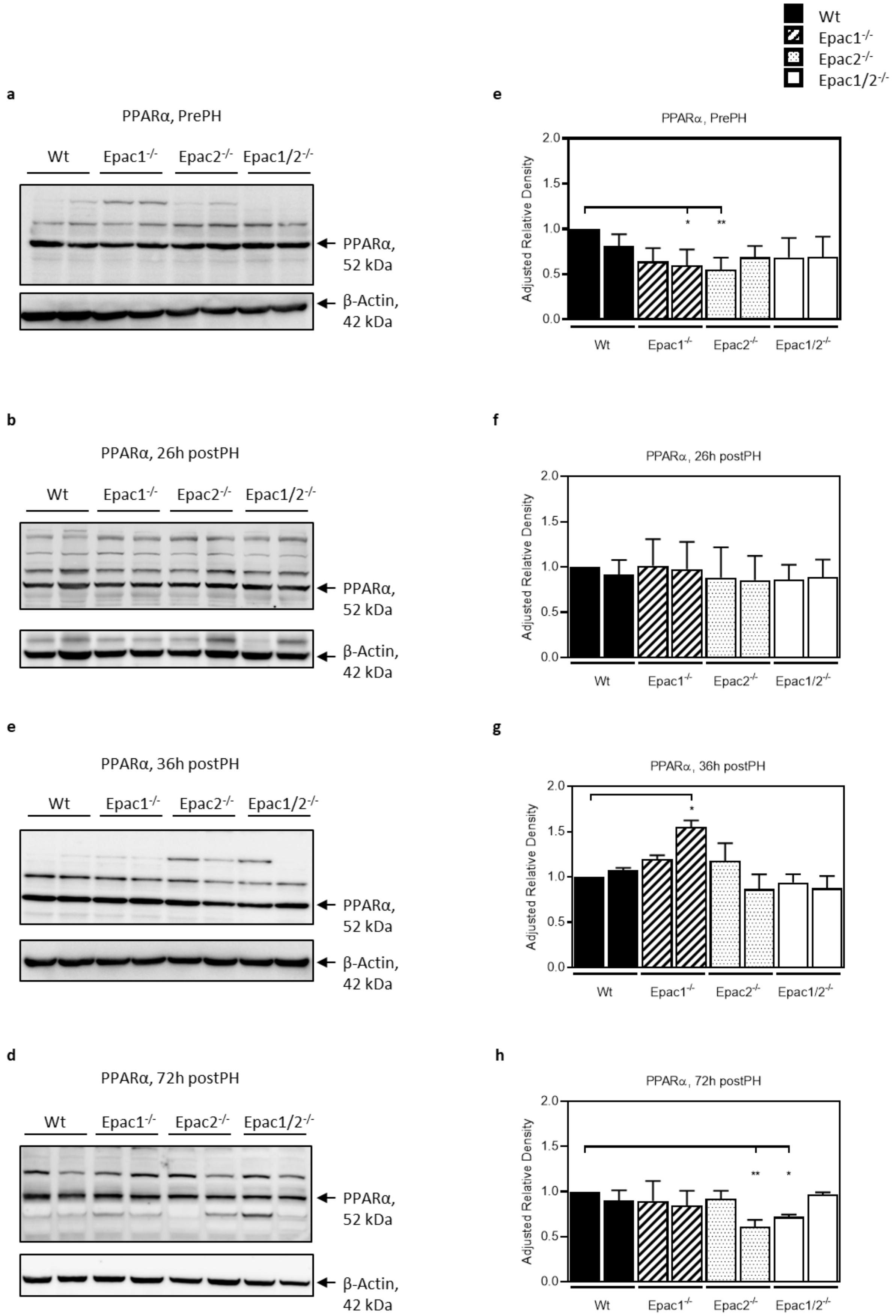

Fig. S9. No alteration in PPARα expression as a consequence of Epac1/2 deficiency. The expression levels of PPARα in wt, Epac1$^{-/-}$, Epac2$^{-/-}$ and Epac1/2$^{-/-}$ mice pre- and post-PH was determined by western blotting (WB). The data is shown as mean(SD), with n=2 mice of each genotype per timepoint. **a-d)** Representative WB of PPARα expression in wt, Epac1$^{-/-}$, Epac2$^{-/-}$ and Epac1/2$^{-/-}$ mice **a)** pre-, **b)** 26h-, **c)** 36h- and **d)** 72h post-PH. **e-h)** Quantification: The WB in a-d) was repeated 3-4 times, and the densiometrically determined PPARα/β-actin ratios were normalized to the value of one wt individual (black bar to the very left) in each blot. **e)** Quantification of **a** PrePH; 4 WB, Kruskal-Wallis with Dunn`s multiple comparison test; Kruskal-Wallis statistic: 14.52, Approximate $^{*}$p=0.0426. **f)** Quantification of **b** 26h postPH; 3 WB, One-way ANOVA with Dunnett`s multiple comparison test, F(7,16)=0.2146, p=0,9767, Brown-Forsythe test, p=0.8564. **g)** Quantification of **c** 36h postPH; 4 WB, Kruskal-Wallis with Dunn`s multiple comparison test; Kruskal-Wallis statistic: 24.20, Approximate $^{**}$p=0.0010. **h)** Quantification of **d** 72h postPH; 3 WB, One-way ANOVA with Dunnett`s multiple comparison test, F(7,16)=3.978, *p=0.0105, Brown-Forsythe test, p=0.2428. The PPARα and β-actin blots displayed in a) are cropped from different parts of the same Hybond™ P 0.45μm PVDF membrane, the same setup applies for PPARα and β-actin bands displayed in b-d) (see Supplementary Fig. S10 the full- length blots).

Supplementary Figures

Fig. S10 Full length blots to Supplementary Fig.S9, PPARα and β-Actin

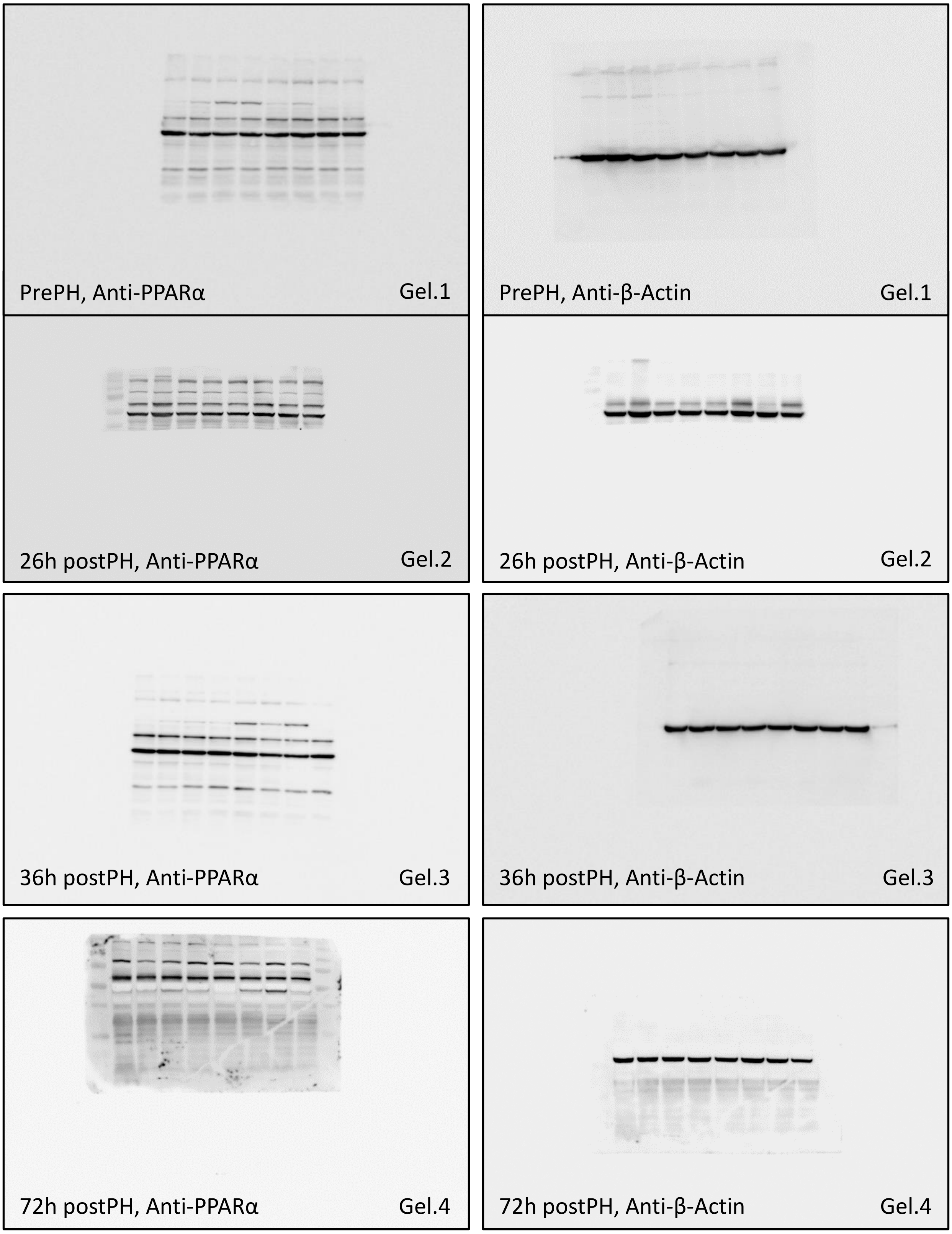

Supplementary Tables

Table 1A

ID	Gene symbol	Protein name
**Sulfation [GO:0051923] (BP)**		
P52840	Sult1a1	Sulfotransferase 1A1
Q9QWG7	Sult1b1	Sulfotransferase family cytosolic 1B member 1
Q3UZZ6	Sult1d1	Sulfotransferase 1 family member D1
**Small molecule catabolic process [GO:0044282] (BP)**		
Q99K67	Aass	Alpha-aminoadipic semialdehyde synthase, mitochondrial; Lysine ketoglutarate reductase; Saccharopine dehydrogenase
Q8R519	Acmsd	2-amino-3-carboxymuconate-6-semialdehyde decarboxylase
Q8VCX1	Akr1d1	3-oxo-5-beta-steroid 4-dehydrogenase
Q9CZS1	Aldh1b1	Aldehyde dehydrogenase X, mitochondrial
Q9EP75	Cyp4f14	Leukotriene-B4 omega-hydroxylase 3
P05201	Got1	Aspartate aminotransferase, cytoplasmic
Q9WU19	Hao1	Hydroxyacid oxidase 1
P49429	Hpd	4-hydroxyphenylpyruvate dioxygenase
P16331	Pah	Phenylalanine-4-hydroxylase
Q9WU79	Prodh	Proline dehydrogenase 1, mitochondrial
Q283N4	Urad	2-oxo-4-hydroxy-4-carboxy-5-ureidoimidazoline decarboxylase
**Isoprenoid biosynthetic process [GO:0008299] (BP)**		
Q8JZK9	Hmgcs1	Hydroxymethylglutaryl-CoA
P58044	Idi1	Isopentenyl-diphosphate Delta-isomerase 1
Q9R008	Mvk	Mevalonate kinase
Q00915	Rbp1	Retinol-binding protein 1
**Organic hydroxy compound metabolic process [GO: 1901615] (BP)**		
Q8VCX1	Akr1d1	3-oxo-5-beta-steroid 4-dehydrogenase
Q9CZS1	Aldh1b1	Aldehyde dehydrogenase X, mitochondrial
Q9EP75	Cyp4f14	Leukotriene-B4 omega-hydroxylase
P05201	Got1	Aspartate aminotransferase
Q8JZK9	Hmgcs1	Hydroxymethylglutaryl-CoA synthase, cytoplasmic
P58044	Idi1	Isopentenyl-diphosphate Delta-isomerase 1
Q9R008	Mvk	Mevalonate kinase
Q00915	Rbp1	Retinol-binding protein 1
Q9QWG7	Sult1b1	Sulfotransferase family cytosolic 1B member 1
Q3UZZ6	Sult1d1	Sulfotransferase 1 family member D1
**Aryl sulfotransferase activity [GO:0004062] (MF)**		
P52840	Sult1a1	Sulfotransferase 1A1
Q9QWG7	Sult1b1	Sulfotransferase family cytosolic 1B member 1
Q3UZZ6	Sult1d1	Sulfotransferase 1 family member D1

Supplementary Table. 1A. Overview of the proteins identified for each GO term for proteins with increased expression pre-PH in Epac1/2$^{-/-}$ mice compared to wt mice.
BP: Biological processes; MF: Molecular function; CC: Cellular compartment.

Table 1B

ID	Gene symbol	Protein name
**Sulfur compound biosynthetic process [GO:0044272] (BP)**		
E9Q4Z2	Acacb	Acetyl-CoA carboxylase 2
Q8QZT1	Acat1	Acetyl-CoA acetyltransferase, mitochondrial
Q91V92	Acly	ATP-citrate synthase
Q9DBE0	Csad	Cysteine sulfinic acid decarboxylase
**Carboxylic ester hydrolase activity [GO:0052689] (MF)**		
O55137	Acot1	Acyl-coenzyme A thioesterase 1
Q9QYR9	Acot2	Acyl-coenzyme A thioesterase 2, mitochondrial
Q8VCT4	Ces1d	Carboxylesterase 1D
Q64176	Ces1e	Carboxylesterase 1E
Q8BK48	Ces2e	Pyrethroid hydrolase Ces2e
O35678	Mgll	Monoglyceride lipase
**Protein homodimerization activity [GO:0042803] (MF)**		
P55096	Abcd3	ATP-binding cassette sub-family D member 3
Q7TMS5	Abcg2	ATP-binding cassette sub-family G member 2
Q8QZT1	Acat1	Acetyl-CoA acetyltransferase, mitochondrial
Q8K4M5	Commd1	COMM domain-containing protein 1
Q9WVJ3	Cpq	Carboxypeptidase Q
P34914	Ephx2	Bifunctional epoxide hydrolase 2;Cytosolic epoxide hydrolase 2;Lipid-phosphate phosphatase
Q00612	G6pdx	Glucose-6-phosphate 1-dehydrogenase X
P13707	Gpd1	Glycerol-3-phosphate dehydrogenase [NAD(+)], cytoplasmic
P51660	Hsd17b4	Peroxisomal multifunctional enzyme type 2;(3R)-hydroxyacyl-CoA dehydrogenase; Enoyl-CoA hydratase 2
O35678	Mgll	Monoglyceride lipase
Q9Z211	Pex11a	Peroxisomal membrane protein 11A
Q9R1Z7	Pts	6-pyruvoyl tetrahydrobiopterin synthase
**Cofactor binding [GO:0048037] (MF)**		
Q8QZT1	Acat1	Acetyl-CoA acetyltransferase, mitochondrial
Q91V92	Acly	ATP-citrate synthase
Q9R0H0	Acox1	Peroxisomal acyl-coenzyme A oxidase 1
O54754	Aox1	Aldehyde oxidase
G3X982	Aox3	Aldehyde oxidase 3
O88668	Creg1	Protein CREG1
Q9DBE0	Csad	Cysteine sulfinic acid decarboxylase
Q00612	G6pdx	Glucose-6-phosphate 1-dehydrogenase X
P13707	Gpd1	Glycerol-3-phosphate dehydrogenase [NAD(+)], cytoplasmic
P06801	Me1	NADP-dependent malic enzyme
**Cellular catabolic process [GO:0044248] (BP)**		
P55096	Abcd3	ATP-binding cassette sub-family D member 3
Q921H8	Acaa1a	3-ketoacyl-CoA thiolase A, peroxisomal
Q8QZT1	Acat1	Acetyl-CoA acetyltransferase, mitochondrial
Q9R0H0	Acox1	Peroxisomal acyl-coenzyme A oxidase 1

O54754	Aox1	Aldehyde oxidase
G3X982	Aox3	Aldehyde oxidase 3
Q8VCT4	Ces1d	Carboxylesterase 1D
Q9WVJ3	Cpq	Carboxypeptidase Q
Q6PEE2	Ctif	CBP80/20-dependent translation initiation factor
O35459	Ech1	Delta(3,5)-Delta(2,4)-dienoyl-CoA isomerase, mitochondrial
P34914	Ephx2	Bifunctional epoxide hydrolase 2;Cytosolic epoxide hydrolase 2;Lipid-phosphate phosphatase
P51660	Hsd17b4	Peroxisomal multifunctional enzyme type 2;(3R)-hydroxyacyl-CoA dehydrogenase; Enoyl-CoA hydratase 2
O35678	Mgll	Monoglyceride lipase
O89023	Tpp1	Tripeptidyl-peptidase 1
Q9QZU9	Ube2l6	Ubiquitin/ISG15-conjugating enzyme E2 L6
**Organic hydroxy compound metabolic process [GO: 1901615] (BP)**		
P47740	Aldh3a2	Fatty aldehyde dehydrogenase
Q8VCT4	Ces1d	Carboxylesterase 1D
Q9WVJ3	Cpq	Carboxypeptidase Q
Q99LB2	Dhrs4	Dehydrogenase/reductase SDR family member 4
Q00612	G6pdx	Glucose-6-phosphate 1-dehydrogenase X
P54869	Hmgcs2	Hydroxymethylglutaryl-CoA synthase, mitochondrial
Q64FW2	Retsat	All-trans-retinol 13,14-reductase
**Oxidoreductase activity, acting on CH-OH group of donors [GO:0016614] (MF)**		
P47740	Aldh3a2	Fatty aldehyde dehydrogenase
O54754	Aox1	Aldehyde oxidase
G3X982	Aox3	Aldehyde oxidase 3
Q99LB2	Dhrs4	Dehydrogenase/reductase SDR family member 4
Q00612	G6pdx	Glucose-6-phosphate 1-dehydrogenase X
P13707	Gpd1	Glycerol-3-phosphate dehydrogenase [NAD (+)], cytoplasmic
P51660	Hsd17b4	Peroxisomal multifunctional enzyme type 2;(3R)-hydroxyacyl-CoA dehydrogenase; Enoyl-CoA hydratase 2
P06801	Me1	NADP-dependent malic enzyme
**Peroxisomal membrane [GO:0005778] (CC)**		
P55096	Abcd3	ATP-binding cassette sub-family D member 3
Q9R0H0	Acox1	Peroxisomal acyl-coenzyme A oxidase 1
Q99LB2	Dhrs4	Dehydrogenase/reductase SDR family member 4
P51660	Hsd17b4	Peroxisomal multifunctional enzyme type 2;(3R)-hydroxyacyl-CoA hydratase 2
Q9Z211	Pex11a	Peroxisomal membrane protein 11A
**Oxidation-reduction process [GO:0055114] (BP)**		
P55096	Abcd3	ATP-binding cassette sub-family D member 3
Q921H8	Acaa1a	3-ketoacyl-CoA thiolase A, peroxisomal
Q8QZT1	Acat1	Acetyl-CoA acetyltransferase, mitochondrial
Q9R0H0	Acox1	Peroxisomal acyl-coenzyme A oxidase 1
P47740	Aldh3a2	Fatty aldehyde dehydrogenase
O54754	Aox1	Aldehyde oxidase
G3X982	Aox3	Aldehyde oxidase 3

O88668	Creg1	Protein CREG1
Q91WL5	Cyp4a12a	Cytochrome P450 4A12A
Q99LB2	Dhrs4	Dehydrogenase/reductase SDR family member 4
O35459	Ech1	Delta(3,5)-Delta(2,4)-dienoyl-CoA isomerase, mitochondrial
Q00612	G6pdx	Glucose-6-phosphate 1-dehydrogenase X
P13707	Gpd1	Glycerol-3-phosphate dehydrogenase [NAD(+)], cytoplasmic
P51660	Hsd17b4	Peroxisomal multifunctional enzyme type 2;(3R)-hydroxyacyl-CoA hydratase 2
P06801	Me1	NADP-dependent malic enzyme
O35678	Mgll	Monoglyceride lipase
Q64FW2	Retsat	All-trans-retinol 13,14-reductase
**Lipid catabolic process [GO:0016042] (BP)**		
P55096	Abcd3	ATP-binding cassette sub-family D member 3
Q921H8	Acaa1a	3-ketoacyl-CoA thiolase A, peroxisomal
Q8QZT1	Acat1	Acetyl-CoA acetyltransferase, mitochondrial
Q9R0H0	Acox1	Peroxisomal acyl-coenzyme A oxidase 1
Q8VCT4	Ces1d	Carboxylesterase 1D
O35459	Ech1	Delta(3,5)-Delta(2,4)-dienoyl-CoA isomerase, mitochondrial
Q00612	G6pdx	Glucose-6-phosphate 1-dehydrogenase X
O35678	Mgll	Monoglyceride lipase
**Coenzyme metabolic process [GO:0006732] (BP)**		
E9Q4Z2	Acacb	Acetyl-CoA carboxylase 2
Q8QZT1	Acat1	Acetyl-CoA acetyltransferase, mitochondrial
Q91V92	Acly	ATP-citrate synthase
O55137	Acot1	Acyl-coenzyme A thioesterase 1
Q9QYR9	Acot2	Acyl-coenzyme A thioesterase 2, mitochondrial
Q8VCT4	Ces1d	Carboxylesterase 1D
Q00612	G6pdx	Glucose-6-phosphate 1-dehydrogenase X
P13707	Gpd1	Glycerol-3-phosphate dehydrogenase [NAD(+)], cytoplasmic
P51660	Hsd17b4	Peroxisomal multifunctional enzyme type 2;(3R)-hydroxyacyl-CoA hydratase 2
Q9R1Z7	Pts	6-pyruvoyl tetrahydrobiopterin synthase
**Thioester metabolic process [GO:0035383] (BP)**		
E9Q4Z2	Acacb	Acetyl-CoA carboxylase 2
Q8QZT1	Acat1	Acetyl-CoA acetyltransferase, mitochondrial
Q91V92	Acly	ATP-citrate synthase
O55137	Acot1	Acyl-coenzyme A thioesterase 1
Q9QYR9	Acot2	Acyl-coenzyme A thioesterase 2, mitochondrial
Q8VCT4	Ces1d	Carboxylesterase 1D
P51660	Hsd17b4	Peroxisomal multifunctional enzyme type 2;(3R)-hydroxyacyl-CoA hydratase 2
**Peroxisome [GO:0005777] (CC)**		
P55096	Abcd3	ATP-binding cassette sub-family D member 3
Q921H8	Acaa1a	3-ketoacyl-CoA thiolase A, peroxisomal
Q9R0H0	Acox1	Peroxisomal acyl-coenzyme A oxidase 1
Q99LB2	Dhrs4	Dehydrogenase/reductase SDR family member 4

O35459	Ech1	Delta(3,5)-Delta(2,4)-dienoyl-CoA isomerase, mitochondrial
P34914	Ephx2	Bifunctional epoxide hydrolase 2;Cytosolic epoxide hydrolase 2;Lipid-phosphate phosphatase
P51660	Hsd17b4	Peroxisomal multifunctional enzyme type 2;(3R)-hydroxyacyl-CoA hydratase 2
Q9Z211	Pex11a	Peroxisomal membrane protein 11A
Q9CYV5	Tmem135	Transmembrane protein 135
**Small molecule metabolic process [GO:0044281] (BP)**		
P55096	Abcd3	ATP-binding cassette sub-family D member 3
Q7TMS5	Abcg2	ATP-binding cassette sub-family G member 2
Q921H8	Acaa1a	3-ketoacyl-CoA thiolase A, peroxisomal
E9Q4Z2	Acacb	Acetyl-CoA carboxylase 2
Q8QZT1	Acat1	Acetyl-CoA acetyltransferase, mitochondrial
Q91V92	Acly	ATP-citrate synthase
O55137	Acot1	Acyl-coenzyme A thioesterase 1
Q9QYR9	Acot2	Acyl-coenzyme A thioesterase 2, mitochondrial
Q9R0H0	Acox1	Peroxisomal acyl-coenzyme A oxidase 1
P47740	Aldh3a2	Fatty aldehyde dehydrogenase
O54754	Aox1	Aldehyde oxidase
G3X982	Aox3	Aldehyde oxidase 3
Q8VCT4	Ces1d	Carboxylesterase 1D
Q9DBE0	Csad	Cysteine sulfinic acid decarboxylase
Q91WL5	Cyp4a12a	Cytochrome P450 4A12A
Q99LB2	Dhrs4	Dehydrogenase/reductase SDR family member 4
O35459	Ech1	Delta(3,5)-Delta(2,4)-dienoyl-CoA isomerase, mitochondrial
Q00612	G6pdx	Glucose-6-phosphate 1-dehydrogenase X
P54869	Hmgcs2	Hydroxymethylglutaryl-CoA synthase, mitochondrial
P51660	Hsd17b4	Peroxisomal multifunctional enzyme type 2;(3R)-hydroxyacyl-CoA hydratase 2
P06801	Me1	NADP-dependent malic enzyme
O35678	Mgll	Monoglyceride lipase
P53808	Pctp	Phosphatidylcholine transfer protein
Q64FW2	Retsat	All-trans-retinol 13,14-reductase
Q920A5	Scpep1	Retinoid-inducible serine carboxypeptidase
P17717	Ugt2b17	UDP-glucuronosyltransferase 2B17
**Cellular lipid metabolic process [GO:0044255] (BP)**		
P55096	Abcd3	ATP-binding cassette sub-family D member 3
Q921H8	Acaa1a	3-ketoacyl-CoA thiolase A, peroxisomal
E9Q4Z2	Acacb	Acetyl-CoA carboxylase 2
Q8QZT1	Acat1	Acetyl-CoA acetyltransferase, mitochondrial
Q91V92	Acly	ATP-citrate synthase
O55137	Acot1	Acyl-coenzyme A thioesterase 1
Q9QYR9	Acot2	Acyl-coenzyme A thioesterase 2, mitochondrial
Q9R0H0	Acox1	Peroxisomal acyl-coenzyme A oxidase 1
P47740	Aldh3a2	Fatty aldehyde dehydrogenase

Q8VCT4	Ces1d	Carboxylesterase 1D
Q91WL5	Cyp4a12a	Cytochrome P450 4A12A
Q99LB2	Dhrs4	Dehydrogenase/reductase SDR family member 4
O35459	Ech1	Delta(3,5)-Delta(2,4)-dienoyl-CoA isomerase, mitochondrial
P34914	Ephx2	Bifunctional epoxide hydrolase 2;Cytosolic epoxide hydrolase 2;Lipid-phosphate phosphatase
P13707	Gpd1	Glycerol-3-phosphate dehydrogenase [NAD(+)], cytoplasmic
P54869	Hmgcs2	Hydroxymethylglutaryl-CoA synthase, mitochondrial
P51660	Hsd17b4	Peroxisomal multifunctional enzyme type 2;(3R)-hydroxyacyl-CoA hydratase 2
O35678	Mgll	Monoglyceride lipase
Q64FW2	Retsat	All-trans-retinol 13,14-reductase
Q920A5	Scpep1	Retinoid-inducible serine carboxypeptidase

Supplementary Table. 1B Overview of the proteins identified for each GO term for proteins with decreased expression pre-PH in Epac1/2$^{-/-}$ mice compared to wt mice.

Table 2

**ID**	**Gene symbol**	**Protein name**
**Sulfur compound binding [GO: 1901681] (MF)**		
Q80XL6	Acad11	Acyl-CoA dehydrogenase family member 11
P51174	Acadl	Long-chain specific acyl-CoA dehydrogenase, mitochondrial
P45952	Acadm	Medium-chain specific acyl-CoA dehydrogenase, mitochondrial
Q9R0H0	Acox1	Peroxisomal acyl-coenzyme A oxidase 1
Q9WUR2	Eci2	Enoyl-CoA delta isomerase 2, mitochondrial
P24472	Gsta4	Glutathione S-transferase A4
**Electron carrier activity [GO:0009055] (MF)**		
Q80XL6	Acad11	Acyl-CoA dehydrogenase family member 11
P51174	Acadl	Long-chain specific acyl-CoA dehydrogenase, mitochondrial
P45952	Acadm	Medium-chain specific acyl-CoA dehydrogenase, mitochondrial
Q9R0H0	Acox1	Peroxisomal acyl-coenzyme A oxidase 1
O54754	Aox1	Aldehyde oxidase
P56392	Cox7a1	Cytochrome c oxidase subunit 7A1, mitochondrial
**Organic hydroxy compound metabolic process [GO: 1901615] (BP)**		
P47740	Aldh3a2	Fatty aldehyde dehydrogenase
Q9WVJ3	Cpq	Carboxypeptidase Q
O88833	Cyp4a10	Cytochrome P450 4A10
Q99LB2	Dhrs4	Dehydrogenase/reductase SDR family member 4
Q9R0N0	Galk1	Galactokinase
P54869	Hmgcs2	Hydroxymethylglutaryl-CoA synthase, mitochondrial
Q64133	Maoa	Amine oxidase [flavin-containing] A
P53808	Pctp	Phosphatidylcholine transfer protein
Q00724	Rbp4	Retinol-binding protein 4
Q64FW2	Retsat	All-trans-retinol 13,14-reductase
**Carnitine metabolic process, CoA-linked [GO:0019254] (BP)**		
P51174	Acadl	Long-chain specific acyl-CoA dehydrogenase, mitochondrial

P45952	Acadm	Medium-chain specific acyl-CoA dehydrogenase, mitochondrial
P47934	Crat	Carnitine O-acetyltransferase
**Dodecenoyl-CoA delta-isomerase activity [GO:0004165] (MF)**		
P42125	Eci1	Enoyl-CoA delta isomerase 1, mitochondrial
Q9WUR2- [illegible]	Eci2	Enoyl-CoA delta isomerase 2, mitochondrial
Q9DBM2	Ehhadh	Peroxisomal bifunctional enzyme; Enoyl-CoA hydratase/3,2-trans-enoyl-CoA isomerase;3-hydroxyacyl-CoA dehydrogenase
**Palmitoyl-CoA hydrolase activity [GO:0016290] (MF)**		
O55137	Acot1	Acyl-coenzyme A thioesterase 1
Q9QYR9	Acot2	Acyl-coenzyme A thioesterase 2, mitochondrial
Q8BWN8	Acot4	Acyl-coenzyme A thioesterase 4
O88531	Ppt1	Palmitoyl-protein thioesterase 1
**Coenzyme binding [GO:0050662] (MF)**		
Q80XL6	Acad11	Acyl-CoA dehydrogenase family member 11
P51174	Acadl	Long-chain specific acyl-CoA dehydrogenase, mitochondrial
P45952	Acadm	Medium-chain specific acyl-CoA dehydrogenase, mitochondrial
Q9R0H0	Acox1	Peroxisomal acyl-coenzyme A oxidase 1
O54754	Aox1	Aldehyde oxidase
Q9CQ62	Decr1	2,4-dienoyl-CoA reductase, mitochondrial
Q9WUR2	Eci2	Enoyl-CoA delta isomerase 2, mitochondrial
P13707	Gpd1	Glycerol-3-phosphate dehydrogenase [NAD(+)], cytoplasmic
Q99L13	Hibadh	3-hydroxyisobutyrate dehydrogenase, mitochondrial
Q9Z2G9	Htatip2	Oxidoreductase HTATIP2
P06801	Me1	NADP-dependent malic enzyme
**Peroxisomal part [GO:0044439] (CC)**		
Q9R0H0	Acox1	Peroxisomal acyl-coenzyme A oxidase 1
Q99LB2	Dhrs4	Dehydrogenase/reductase SDR family member 4
Q9WUR2	Eci2	Enoyl-CoA delta isomerase 2, mitochondrial
P46412	Gpx3	Glutathione peroxidase 3
P51660	Hsd17b4	Peroxisomal multifunctional enzyme type 2;(3R)-hydroxyacyl-CoA hydratase 2
Q9Z211	Pex11a	Peroxisomal membrane protein 11A
Q9D0K1	Pex13	Peroxisomal membrane protein PEX13
Q91XC9	Pex16	Peroxisomal membrane protein PEX16
Q8VCI5	Pex19	Peroxisomal biogenesis factor 19
**Mitochondrion [GO:0005739] (CC)**		
Q3UNZ8		Quinone oxidoreductase-like protein 2
Q8R2Y0	Abhd6	Monoacylglycerol lipase ABHD6
Q921H8	Acaa1a	3-ketoacyl-CoA thiolase A, peroxisomal
Q8VCH0	Acaa1b	3-ketoacyl-CoA thiolase B, peroxisomal
Q80XL6	Acad11	Acyl-CoA dehydrogenase family member 11
P51174	Acadl	Long-chain specific acyl-CoA dehydrogenase, mitochondrial
P45952	Acadm	Medium-chain specific acyl-CoA dehydrogenase, mitochondrial

O55137	Acot1	Acyl-coenzyme A thioesterase 1
Q9QYR9	Acot2	Acyl-coenzyme A thioesterase 2, mitochondrial
Q9R0H0	Acox1	Peroxisomal acyl-coenzyme A oxidase 1
P41216	Acsl1	Long-chain-fatty-acid--CoA ligase 1
Q14DH7	Acss3	Acyl-CoA synthetase short-chain family member 3, mitochondrial
Q80V03	Adck5	Uncharacterized aarF domain-containing protein kinase 5
P47740	Aldh3a2	Fatty aldehyde dehydrogenase
Q80XN0	Bdh1	D-beta-hydroxybutyrate dehydrogenase, mitochondrial
P56392	Cox7a1	Cytochrome c oxidase subunit 7A1, mitochondrial
P52825	Cpt2	Carnitine O-palmitoyltransferase 2, mitochondrial
P47934	Crat	Carnitine O-acetyltransferase
Q9CWS0	Ddah1	N(G),N(G)-dimethylarginine dimethylaminohydrolase 1
Q9CQ62	Decr1	2,4-dienoyl-CoA reductase, mitochondrial
Q99LB2	Dhrs4	Dehydrogenase/reductase SDR family member 4
O35459	Ech1	Delta(3,5)-Delta(2,4)-dienoyl-CoA isomerase, mitochondrial
P42125	Eci1	Enoyl-CoA delta isomerase 1, mitochondrial
Q9WUR2	Eci2	Enoyl-CoA delta isomerase 2, mitochondrial
Q9DBM2	Ehhadh	Peroxisomal bifunctional enzyme; Enoyl-CoA hydratase/3,2-trans-enoyl-CoA isomerase;3-hydroxyacyl-CoA dehydrogenase
Q5FW57	Gm4952	Glycine N-acyltransferase-like protein
P13707	Gpd1	Glycerol-3-phosphate dehydrogenase [NAD(+)], cytoplasmic
P24472	Gsta4	Glutathione S-transferase A4
Q99L13	Hibadh	3-hydroxyisobutyrate dehydrogenase, mitochondrial
P54869	Hmgcs2	Hydroxymethylglutaryl-CoA synthase, mitochondrial
P51660	Hsd17b4	Peroxisomal multifunctional enzyme type 2;(3R)-hydroxyacyl-CoA dehydrogenase; Enoyl-CoA hydratase 2
Q64133	Maoa	Amine oxidase [flavin-containing] A
P06801	Me1	NADP-dependent malic enzyme
Q9CQL4	Mrpl20	39S ribosomal protein L20, mitochondrial
Q9R1Z7	Pts	6-pyruvoyl tetrahydrobiopterin synthase
Q9QZD8	Slc25a10	Mitochondrial dicarboxylate carrier
Q9Z2Z6	Slc25a20	Mitochondrial carnitine/acylcarnitine carrier protein
Q8CC88	Vwa8	von Willebrand factor A domain-containing protein 8
**Oxidoreductase activity [GO:0016491] (MF)**		
Q3UNZ8		Quinone oxidoreductase-like protein 2
Q921H8	Acaa1a	3-ketoacyl-CoA thiolase A, peroxisomal
Q80XL6	Acad11	Acyl-CoA dehydrogenase family member 11
P51174	Acadl	Long-chain specific acyl-CoA dehydrogenase, mitochondrial
P45952	Acadm	Medium-chain specific acyl-CoA dehydrogenase, mitochondrial
Q9R0H0	Acox1	Peroxisomal acyl-coenzyme A oxidase 1
P47740	Aldh3a2	Fatty aldehyde dehydrogenase
O54754	Aox1	Aldehyde oxidase
Q80XN0	Bdh1	D-beta-hydroxybutyrate dehydrogenase, mitochondrial
P56392	Cox7a1	Cytochrome c oxidase subunit 7A1, mitochondrial
O88668	Creg1	Protein CREG1

O88833	Cyp4a10	Cytochrome P450 4A10
Q91WL5	Cyp4a12a	Cytochrome P450 4A12A
O35728	Cyp4a14	Cytochrome P450 4A14
Q9CQ62	Decr1	2,4-dienoyl-CoA reductase, mitochondrial
Q9WV68	Decr2	Peroxisomal 2,4-dienoyl-CoA reductase
Q99LB2	Dhrs4	Dehydrogenase/reductase SDR family member 4
Q9DBM2	Ehhadh	Peroxisomal bifunctional enzyme; Enoyl-CoA hydratase/3,2-trans-enoyl-CoA isomerase;3-hydroxyacyl-CoA dehydrogenase
P13707	Gpd1	Glycerol-3-phosphate dehydrogenase [NAD(+)], cytoplasmic
P46412	Gpx3	Glutathione peroxidase 3
Q99L13	Hibadh	3-hydroxyisobutyrate dehydrogenase, mitochondrial
P51660	Hsd17b4	Peroxisomal multifunctional enzyme type 2;(3R)-hydroxyacyl-CoA dehydrogenase; Enoyl-CoA hydratase 2
Q9Z2G9	Htatip2	Oxidoreductase HTATIP2
Q64133	Maoa	Amine oxidase [flavin-containing] A
P06801	Me1	NADP-dependent malic enzyme
Q64FW2	Retsat	All-trans-retinol 13,14-reductase
**Oxidation-reduction process [GO:0055114] (BP)**		
Q3UNZ8		Quinone oxidoreductase-like protein 2
Q921H8	Acaa1a	3-ketoacyl-CoA thiolase A, peroxisomal
Q8VCH0	Acaa1b	3-ketoacyl-CoA thiolase B, peroxisomal
Q80XL6	Acad11	Acyl-CoA dehydrogenase family member 11
P51174	Acadl	Long-chain specific acyl-CoA dehydrogenase, mitochondrial
P45952	Acadm	Medium-chain specific acyl-CoA dehydrogenase, mitochondrial
Q9R0H0	Acox1	Peroxisomal acyl-coenzyme A oxidase 1
P47740	Aldh3a2	Fatty aldehyde dehydrogenase
O54754	Aox1	Aldehyde oxidase
Q80XN0	Bdh1	D-beta-hydroxybutyrate dehydrogenase, mitochondrial
Q923D2	Blvrb	Flavin reductase (NADPH)
O88668	Creg1	Protein CREG1
O88833	Cyp4a10	Cytochrome P450 4A10
Q91WL5	Cyp4a12a	Cytochrome P450 4A12A
O35728	Cyp4a14	Cytochrome P450 4A14
Q9CQ62	Decr1	2,4-dienoyl-CoA reductase, mitochondrial
Q9WV68	Decr2	Peroxisomal 2,4-dienoyl-CoA reductase
Q99LB2	Dhrs4	Dehydrogenase/reductase SDR family member 4
O35459	Ech1	Delta(3,5)-Delta(2,4)-dienoyl-CoA isomerase, mitochondrial
P42125	Eci1	Enoyl-CoA delta isomerase 1, mitochondrial
Q9DBM2	Ehhadh	Peroxisomal bifunctional enzyme; Enoyl-CoA hydratase/3,2-trans-enoyl-CoA isomerase;3-hydroxyacyl-CoA dehydrogenase
P13707	Gpd1	Glycerol-3-phosphate dehydrogenase [NAD(+)], cytoplasmic
P46412	Gpx3	Glutathione peroxidase 3
Q99L13	Hibadh	3-hydroxyisobutyrate dehydrogenase, mitochondrial
P51660	Hsd17b4	Peroxisomal multifunctional enzyme type 2;(3R)-hydroxyacyl-CoA dehydrogenase; Enoyl-CoA hydratase 2

Q9Z2G9	Htatip2	Oxidoreductase HTATIP2
Q64133	Maoa	Amine oxidase [flavin-containing] A
P06801	Me1	NADP-dependent malic enzyme
Q9D0K1	Pex13	Peroxisomal membrane protein PEX13
Q64FW2	Retsat	All-trans-retinol 13,14-reductase
**Lipid catabolic process [GO:0016042] (BP)**		
Q8R2Y0-[illegible]	Abhd6	Monoacylglycerol lipase ABHD6
Q921H8	Acaa1a	3-ketoacyl-CoA thiolase A, peroxisomal
Q8VCH0	Acaa1b	3-ketoacyl-CoA thiolase B, peroxisomal
Q80XL6	Acad11	Acyl-CoA dehydrogenase family member 11
P51174	Acadl	Long-chain specific acyl-CoA dehydrogenase, mitochondrial
P45952	Acadm	Medium-chain specific acyl-CoA dehydrogenase, mitochondrial
Q9R0H0	Acox1	Peroxisomal acyl-coenzyme A oxidase 1
O88833	Cyp4a10	Cytochrome P450 4A10
Q9CQ62	Decr1	2,4-dienoyl-CoA reductase, mitochondrial
O35459	Ech1	Delta(3,5)-Delta(2,4)-dienoyl-CoA isomerase, mitochondrial
P42125	Eci1	Enoyl-CoA delta isomerase 1, mitochondrial
Q9DBM2	Ehhadh	Peroxisomal bifunctional enzyme; Enoyl-CoA hydratase/3,2-trans-enoyl-CoA isomerase;3-hydroxyacyl-CoA dehydrogenase
P51660	Hsd17b4	Peroxisomal multifunctional enzyme type 2;(3R)-hydroxyacyl-CoA dehydrogenase; Enoyl-CoA hydratase 2
Q99PI5	Lpin2	Phosphatidate phosphatase LPIN2
Q9D0K1	Pex13	Peroxisomal membrane protein PEX13
O88531	Ppt1	Palmitoyl-protein thioesterase 1
**Organic acid catabolic process [GO:0016054] (BP)**		
Q921H8	Acaa1a	3-ketoacyl-CoA thiolase A, peroxisomal
Q8VCH0	Acaa1b	3-ketoacyl-CoA thiolase B, peroxisomal
Q80XL6	Acad11	Acyl-CoA dehydrogenase family member 11
P51174	Acadl	Long-chain specific acyl-CoA dehydrogenase, mitochondrial
P45952	Acadm	Medium-chain specific acyl-CoA dehydrogenase, mitochondrial
Q8BWN8	Acot4	Acyl-coenzyme A thioesterase 4
Q9R0H0	Acox1	Peroxisomal acyl-coenzyme A oxidase 1
Q9DBE0	Csad	Cysteine sulfinic acid decarboxylase
O88833	Cyp4a10	Cytochrome P450 4A10
Q9CWS0	Ddah1	N(G),N(G)-dimethylarginine dimethylaminohydrolase 1
Q9CQ62	Decr1	2,4-dienoyl-CoA reductase, mitochondrial
O35459	Ech1	Delta(3,5)-Delta(2,4)-dienoyl-CoA isomerase, mitochondrial
P42125	Eci1	Enoyl-CoA delta isomerase 1, mitochondrial
Q9DBM2	Ehhadh	Peroxisomal bifunctional enzyme; Enoyl-CoA hydratase/3,2-trans-enoyl- [illegible]
Q99L13	Hibadh	3-hydroxyisobutyrate dehydrogenase, mitochondrial
P51660	Hsd17b4	Peroxisomal multifunctional enzyme type 2;(3R)-hydroxyacyl-CoA hydratase 2
Q99PI5	Lpin2	Phosphatidate phosphatase LPIN2
Q9D0K1	Pex13	Peroxisomal membrane protein PEX13

**Monocarboxylic acid metabolic process [GO:0032787] (BP)**		
Q921H8	Acaa1a	3-ketoacyl-CoA thiolase A, peroxisomal
Q8VCH0	Acaa1b	3-ketoacyl-CoA thiolase B, peroxisomal
Q80XL6	Acad11	Acyl-CoA dehydrogenase family member 11
P51174	Acadl	Long-chain specific acyl-CoA dehydrogenase, mitochondrial
P45952	Acadm	Medium-chain specific acyl-CoA dehydrogenase, mitochondrial
O55137	Acot1	Acyl-coenzyme A thioesterase 1
Q9QYR9	Acot2	Acyl-coenzyme A thioesterase 2, mitochondrial
Q8BWN8	Acot4	Acyl-coenzyme A thioesterase 4
Q9R0H0	Acox1	Peroxisomal acyl-coenzyme A oxidase 1
P41216	Acsl1	Long-chain-fatty-acid--CoA ligase 1
P52825	Cpt2	Carnitine O-palmitoyltransferase 2, mitochondrial
P47934	Crat	Carnitine O-acetyltransferase
O88833	Cyp4a10	Cytochrome P450 4A10
Q91WL5	Cyp4a12a	Cytochrome P450 4A12A
Q9CQ62	Decr1	2,4-dienoyl-CoA reductase, mitochondrial
Q9WV68	Decr2	Peroxisomal 2,4-dienoyl-CoA reductase
O35459	Ech1	Delta(3,5)-Delta(2,4)-dienoyl-CoA isomerase, mitochondrial
P42125	Eci1	Enoyl-CoA delta isomerase 1, mitochondrial
Q9DBM2	Ehhadh	Peroxisomal bifunctional enzyme; Enoyl-CoA hydratase/3,2-trans-enoyl-CoA isomerase;3-hydroxyacyl-CoA dehydrogenase
Q9R0N0	Galk1	Galactokinase
P51660	Hsd17b4	Peroxisomal multifunctional enzyme type 2;(3R)-hydroxyacyl-CoA hydratase 2
Q99PI5	Lpin2	Phosphatidate phosphatase LPIN2
Q9D0K1	Pex13	Peroxisomal membrane protein PEX13
Q3UP75	Ugt3a1	UDP-glucuronosyltransferase 3A1
Q9Z0K8	Vnn1	Pantetheinase
**Cellular lipid metabolic process [GO:0044255] (BP)**		
Q8R2Y0	Abhd6	Monoacylglycerol lipase ABHD6
Q921H8	Acaa1a	3-ketoacyl-CoA thiolase A, peroxisomal
Q8VCH0	Acaa1b	3-ketoacyl-CoA thiolase B, peroxisomal
Q80XL6	Acad11	Acyl-CoA dehydrogenase family member 11
P51174	Acadl	Long-chain specific acyl-CoA dehydrogenase, mitochondrial
P45952	Acadm	Medium-chain specific acyl-CoA dehydrogenase, mitochondrial
O55137	Acot1	Acyl-coenzyme A thioesterase 1
Q9QYR9	Acot2	Acyl-coenzyme A thioesterase 2, mitochondrial
Q8BWN8	Acot4	Acyl-coenzyme A thioesterase 4
Q9R0H0	Acox1	Peroxisomal acyl-coenzyme A oxidase 1
P41216	Acsl1	Long-chain-fatty-acid--CoA ligase 1
P47740	Aldh3a2	Fatty aldehyde dehydrogenase
P52825	Cpt2	Carnitine O-palmitoyltransferase 2, mitochondrial
P47934	Crat	Carnitine O-acetyltransferase
O88833	Cyp4a10	Cytochrome P450 4A10
Q91WL5	Cyp4a12a	Cytochrome P450 4A12A

Q9CQ62	Decr1	2,4-dienoyl-CoA reductase, mitochondrial
Q9WV68	Decr2	Peroxisomal 2,4-dienoyl-CoA reductase
Q99LB2	Dhrs4	Dehydrogenase/reductase SDR family member 4
O35459	Ech1	Delta(3,5)-Delta(2,4)-dienoyl-CoA isomerase, mitochondrial
P42125	Eci1	Enoyl-CoA delta isomerase 1, mitochondrial
Q9DBM2	Ehhadh	Peroxisomal bifunctional enzyme; Enoyl-CoA hydratase/3,2-trans-enoyl-CoA isomerase;3-hydroxyacyl-CoA dehydrogenase
P34914	Ephx2	Bifunctional epoxide hydrolase 2;Cytosolic epoxide hydrolase 2;Lipid-phosphate phosphatase
P13707	Gpd1	Glycerol-3-phosphate dehydrogenase [NAD(+)], cytoplasmic
P54869	Hmgcs2	Hydroxymethylglutaryl-CoA synthase, mitochondrial
P51660	Hsd17b4	Peroxisomal multifunctional enzyme type 2;(3R)-hydroxyacyl-CoA hydratase 2
Q99PI5	Lpin2	Phosphatidate phosphatase LPIN2
Q9D0K1	Pex13	Peroxisomal membrane protein PEX13
Q00724	Rbp4	Retinol-binding protein 4
Q64FW2	Retsat	All-trans-retinol 13,14-reductase
**Microbody [GO:0042579] (CC)**		
Q921H8	Acaa1a	3-ketoacyl-CoA thiolase A, peroxisomal
Q8VCH0	Acaa1b	3-ketoacyl-CoA thiolase B, peroxisomal
Q80XL6	Acad11	Acyl-CoA dehydrogenase family member 11
P45952	Acadm	Medium-chain specific acyl-CoA dehydrogenase, mitochondrial
Q8BWN8	Acot4	Acyl-coenzyme A thioesterase 4
Q9R0H0	Acox1	Peroxisomal acyl-coenzyme A oxidase 1
P41216	Acsl1	Long-chain-fatty-acid--CoA ligase 1
P47934	Crat	Carnitine O-acetyltransferase
Q99LB2	Dhrs4	Dehydrogenase/reductase SDR family member 4
O35459	Ech1	Delta(3,5)-Delta(2,4)-dienoyl-CoA isomerase, mitochondrial
Q9WUR2	Eci2	Enoyl-CoA delta isomerase 2, mitochondrial
Q9DBM2	Ehhadh	Peroxisomal bifunctional enzyme; Enoyl-CoA hydratase/3,2-trans-enoyl-CoA isomerase;3-hydroxyacyl-CoA dehydrogenase
P34914	Ephx2	Bifunctional epoxide hydrolase 2;Cytosolic epoxide hydrolase 2;Lipid-phosphate phosphatase
P46412	Gpx3	Glutathione peroxidase 3
P51660	Hsd17b4	Peroxisomal multifunctional enzyme type 2;(3R)-hydroxyacyl-CoA dehydrogenase; Enoyl-CoA hydratase 2
Q9Z211	Pex11a	Peroxisomal membrane protein 11A
Q9D0K1	Pex13	Peroxisomal membrane protein PEX13
Q91XC9	Pex16	Peroxisomal membrane protein PEX16
Q8VCI5	Pex19	Peroxisomal biogenesis factor 19

Supplementary Table. 2. Overview of the proteins identified for each GO term for proteins with decreased expression post-PH in Epac1/2$^{-/-}$ mice compared to wt mice.

Table 3.

ID	Gene symbol	Protein name	prePH FC	postPH FC
Q99K67	Aass	Alpha-aminoadipic semialdehyde synthase, mitochondrial;Lysine ketoglutarate reductase;Saccharopine dehydrogenase	-0.80	-0.96, ns
P55096	Abcd3	ATP-binding cassette sub-family D member 3	-1.29	-1.19, ns
Q7TMS5	Abcg2	ATP-binding cassette sub-family G member 2	-1.40	-1.27,ns
Q8R2Y0	Abhd6	Monoacylglycerol lipase ABHD6	-1.28, ns	-1.22
Q80XL6	Acad11	Acyl-CoA dehydrogenase family member 11	-1.23	-1.41
P51174	Acadl	Long-chain specific acyl-CoA dehydrogenase, mitochondrial	-1.11, ns	-1.24
P45952	Acadm	Medium-chain specific acyl-CoA dehydrogenase, mitochondrial	-1.08, ns	-1.25
Q8QZT1	Acat1	Acetyl-CoA acetyltransferase, mitochondrial	-1.21	-1.19, ns
Q9QYR9	Acot2	Acyl-coenzyme A thioesterase 2, mitochondrial	-1.61	-4.18
Q8BWN8	Acot4	Acyl-coenzyme A thioesterase 4	-1.78, ns	-1.95
P41216	Acsl1	Long-chain-fatty-acid--CoA ligase 1	-1.13, ns	-1.24
Q14DH7	Acss3	Acyl-CoA synthetase short-chain family member 3, mitochondrial	-1.63	-1.83
Q9CZS1	Aldh1b1	Aldehyde dehydrogenase X, mitochondrial	-0.60	-0.61,ns
P47740	Aldh3a2	Fatty aldehyde dehydrogenase	-1.48	-2.75
Q80XN0	Bdh1	D-beta-hydroxybutyrate dehydrogenase, mitochondrial	-1.28	-1.28
P16330	Cnp	2,3-cyclic-nucleotide 3-phosphodiesterase	0.72	0.84, ns
P56392	Cox7a1	Cytochrome c oxidase subunit 7A1, mitochondrial	-1.44	-1.32
P52825	Cpt2	Carnitine O-palmitoyltransferase 2, mitochondrial	-1.02, ns	-1.24
P47934	Crat	Carnitine O-acetyltransferase	-1.59, ns	-1.92
Q9CWS0	Ddah1	N(G),N(G)-dimethylarginine dimethylaminohydrolase 1	-1.58, ns	-1.55
Q9CQ62	Decr1	2,4-dienoyl-CoA reductase, mitochondrial	-1.19, ns	-1.31
O35459	Ech1	Delta(3,5)-Delta(2,4)-dienoyl-CoA isomerase, mitochondrial	-1.31	-1.35
P42125	Eci1	Enoyl-CoA delta isomerase 1, mitochondrial	-1.16, ns	-1,36
Q9WUR2	Eci2	Enoyl-CoA delta isomerase 2, mitochondrial	-1.23, ns	-1.34
Q5FW57	Gm4952	Glycine N-acyltransferase-like protein	-1.33, ns	1.40
Q99L13	Hibadh	3-hydroxyisobutyrate dehydrogenase, mitochondrial	-1.00, ns	-1.27
P54869	Hmgcs2	Hydroxymethylglutaryl-CoA synthase, mitochondrial	-1.29	-1.41
Q61941	Nnt	NAD(P) transhydrogenase, mitochondrial	-21.01,ns	-23.72,ns
Q64133	Maoa	Amine oxidase [flavin-containing] A	-1.32,ns	-1.36
Q9CQL4	Mrpl20	39S ribosomal protein L20, mitochondrial	-1.08,ns	-1.33

Q9R1Z7	Pts	6-pyruvoyl tetrahydrobiopterin synthase	1.21	1.32
Q9QZD8	Slc25a10	Mitochondrial dicarboxylate carrier	-1.28	-1.37
Q8BH59	Slc25a12	Calcium-binding mitochondrial carrier protein Aralar1	-0.81,ns	-0.89
Q9Z2Z6	Slc25a20	Mitochondrial carnitine/acylcarnitine carrier protein	-1.01, ns	-1.33
Q9CQ85	Timm22	Mitochondrial import inner membrane translocase subunit Tim22	-1.26	-1.19, ns
Q9CYG7	Tomm34	Mitochondrial import receptor subunit TOM34	-1.02, ns	-1.24
P17717	Ugt2b17	UDP-glucuronosyltransferase 2B17	-1.28	-1.24,ns

Supplementary Table. 3. List of mitochondrial genes that are less expressed in Epac1/2$^{-/-}$ mice. PrePH (WTprePH *vs.* Epac1/2$^{-/-}$prePH), postPH (WTpostPH *vs.* Epac1/2$^{-/-}$postPH), negative value: decreased protein expression in Epac1/2$^{-/-}$ mice relative to wt mice. ns: not significant. FC: fold change. Please see the Supplementary M&M section for details regarding statistical analysis.

References

1 Cardiff, R. D., Miller, C. H. & Munn, R. J. Manual hematoxylin and eosin staining of mouse tissue sections. *Cold Spring Harbor protocols* **2014**, 655-658, doi:10.1101/pdb.prot073411 (2014).

2 Mehlem, A., Hagberg, C. E., Muhl, L., Eriksson, U. & Falkevall, A. Imaging of neutral lipids by oil red O for analyzing the metabolic status in health and disease. *Nature protocols* **8**, 1149-1154, doi:10.1038/nprot.2013.055 (2013).

3 Bligh, E. G. & Dyer, W. J. A rapid method of total lipid extraction and purification. *Canadian journal of biochemistry and physiology* **37**, 911-917, doi:10.1139/o59-099 (1959).

4 Greenhalgh, S. N. *et al.* Loss of Integrin alphavbeta8 in Murine Hepatocytes Accelerates Liver Regeneration. *Am J Pathol* **189**, 258-271, doi:10.1016/j.ajpath.2018.10.007 (2019).

5 Wisniewski, J. R., Zougman, A., Nagaraj, N. & Mann, M. Universal sample preparation method for proteome analysis. *Nature methods* **6**, 359-362, doi:10.1038/nmeth.1322 (2009).

6 Hernandez-Valladares, M. *et al.* Reliable FASP-based procedures for optimal quantitative proteomic and phosphoproteomic analysis on samples from acute myeloid leukemia patients. *Biological procedures online* **18**, 13, doi:10.1186/s12575-016-0043-0 (2016).

7 Cox, J. *et al.* Andromeda: a peptide search engine integrated into the MaxQuant environment. *Journal of proteome research* **10**, 1794-1805, doi:10.1021/pr101065j (2011).

8 Aasebo, E. *et al.* Freezing effects on the acute myeloid leukemia cell proteome and phosphoproteome revealed using optimal quantitative workflows. *Journal of proteomics* **145**, 214-225, doi:10.1016/j.jprot.2016.03.049 (2016).

9 Cox, J. *et al.* Accurate proteome-wide label-free quantification by delayed normalization and maximal peptide ratio extraction, termed MaxLFQ. *Molecular & cellular proteomics : MCP* **13**, 2513-2526, doi:10.1074/mcp.M113.031591 (2014).

10 Tyanova, S. *et al.* The Perseus computational platform for comprehensive analysis of (prote)omics data. *Nature methods* **13**, 731-740, doi:10.1038/nmeth.3901 (2016).

11 Arntzen, M. O. *et al.* IsobariQ: software for isobaric quantitative proteomics using IPTL, iTRAQ, and TMT. *Journal of proteome research* **10**, 913-920, doi:10.1021/pr1009977 (2011).

12 Scholz, C. *et al.* Avoiding abundance bias in the functional annotation of post-translationally modified proteins. *Nature methods* **12**, 1003-1004, doi:10.1038/nmeth.3621 (2015).

Author contributions: KSA, LP: Planning and execution of experiments, preparation of manuscript; RA, HM, EA, IKNP, LH, RD, SJ, RKB, NH, FS: Planning and execution of experiments; SOD: Planning and financing study, preparing manuscript; MB: Coordinating, planning and financing study, preparing manuscript. All authors read and approved the final manuscript.

Competing interests: The authors declare no competing interests.
